# Development and Validation of an Ability Measure of Emotion Understanding: The Core Relational Themes of Emotion (CORE) Test

**DOI:** 10.3390/jintelligence11100195

**Published:** 2023-10-09

**Authors:** James L. Floman, Marc A. Brackett, Matthew L. LaPalme, Annette R. Ponnock, Sigal G. Barsade, Aidan Doyle

**Affiliations:** 1 Yale Center for Emotional Intelligence, Yale University, New Haven, CT 06511, USA; 2 Wharton School, University of Pennsylvania, Philadelphia, PA 19104, USA

**Keywords:** emotion understanding ability, emotional intelligence test, emotion knowledge, emotion appraisals, core relational themes, emotional granularity, Semantic Space Theory

## Abstract

Emotion understanding (EU) ability is associated with healthy social functioning and psychological well-being. Across three studies, we develop and present validity evidence for the Core Relational Themes of Emotions (CORE) Test. The test measures people’s ability to identify relational themes underlying 19 positive and negative emotions. Relational themes are consistencies in the meaning people assign to emotional experiences. In Study 1, we developed and refined the test items employing a literature review, expert panel, and confusion matrix with a demographically diverse sample. Correctness criteria were determined using theory and prior research, and a progressive (degrees of correctness) paradigm was utilized to score the test. In Study 2, the CORE demonstrated high internal consistency and a confirmatory factor analysis supported the unidimensional factor structure. The CORE showed evidence of convergence with established EU ability measures and divergent relationships with verbal intelligence and demographic characteristics, supporting its construct validity. Also, the CORE was associated with less relational conflict. In Study 3, the CORE was associated with more adaptive and less maladaptive coping and higher well-being on multiple indicators. A set of effects remained, accounting for variance from a widely used EU test, supporting the CORE’s incremental validity. Theoretical and methodological contributions are discussed.

“To feel these feelings at the right time, on the right occasion, towards the right people, for the right purpose and in the right manner…is the mark of virtue.”

—Aristotle (2009, 353 BC)

## 1. Introduction

Knowing how to utilize emotions to guide skillful action has been a pillar of human wisdom for millennia. Over the past few decades, theoretical and methodological advances in research on emotional expertise has grown rapidly in affective science (Hoemann et al. 2021a). There is a particular interest in people’s emotion understanding (EU) ability. EU ability is an umbrella term that incorporates a suite of interrelated emotion skills. These skills include recognizing patterns in the causes and consequences of emotions, knowing the various ways that emotions are akin to and distinguished from one another, and representing emotional experiences with precision and granularity using language (Castro et al. 2016; Kashdan et al. 2015; Mayer et al. 2016; Tugade et al. 2004). 

The Cascading Model of Emotional Intelligence (EI; Joseph and Newman 2010) suggests that EU ability is central to how all emotional abilities operate. The model proposes that emotional abilities build upon and facilitate each other. The ability to accurately perceive emotions in faces, voices, and body movements provides rich information that one interprets and makes sense of using EU ability. After gathering emotion-laden information and parsing its meaning, people can then employ these data to direct behavior and regulate emotions in ways that suit personal goals or situational demands. Given that goals and environments are dynamic, the capacity to continuously update behavior based on new emotional information promotes flexibility and adaptability. EU ability, therefore, serves as a key link between perceiving emotions and knowing how to best manage them.

Furthermore, research on emotion granularity suggests that EU ability is associated with beneficial social and well-being outcomes by supporting targeted, adaptive emotion regulation (Kashdan et al. 2015; Tugade et al. 2004). The granular emotion knowledge gleaned from EU informs judgment and decision-making, driving specific regulatory behaviors that impact many life outcomes (e.g., Hu et al. 2014; Webb et al. 2012). In support of these models, meta-analyses and recent studies show that higher EU is associated with healthier emotion regulation, and more adaptive and less maladaptive coping, as well as other desirable outcomes, including supportive relationships, job performance, psychological well-being, and even physical health (Fernández-Berrocal and Extremera 2016; Hoemann et al. 2021b; Kotsou et al. 2019; Sánchez-Álvarez et al. 2016). Although more research is needed both on cascading and granularity accounts of emotional abilities, EU ability plays a central role in skillfully navigating emotions to meet a variety of demands. 

Given the value of EU ability, the quality of the measurement tools that assess this construct is of scientific and real-world significance. By quality, we mean the psychometric standards of reliability and validity (AERA et al. 2014). Scholars have noted limitations in the existing EU ability tests (e.g., Castro et al. 2016). These limitations include: (1) measuring multiple facets of EU ability and then aggregating them into a single mean score, preventing the study of specific facets of EU ability; (2) relying on situation-based vignettes, rather than tapping emotion knowledge directly; (3) employing dichotomous scoring, despite the complex interconnected structure of emotion concepts (Cowen and Keltner 2017, 2021); (4) having low reliability, reducing their statistical power; and (5) being primarily validated with White, college-attending or college-educated samples, not representing the diversity of respondents in many populations. These issues may limit the scope, precision, and generalizability of research on the nature of EU ability, including how it relates to other EI abilities and primary outcomes of interest (AERA et al. 2014). 

Across three studies, we develop and present validity evidence for a novel performance measure of EU ability, the Core Relational Themes of Emotion (CORE) Test.^1^ The CORE assesses people’s ability to identify *core relational themes* (Campos et al. 2013; Lazarus 1991; Smith and Lazarus 1993), which are primary meanings or semantic features underlying 19 different positive and negative emotions. The specific core relational themes were drawn from an in-depth review of the emotion science literature. We aimed to address some of the noted limitations of current measures to advance research in the field. In the following section, we review appraisal theories of emotion and the literature on core relational themes to establish the theoretical basis for the development of the CORE.

### 1.1. Appraisal Theories of Emotion and Core Relational Themes

Cognitive appraisals are evaluations of the proximity and nature of threats and opportunities in the environment that activate particular emotions (Moors 2020). From an appraisal perspective, the subjective evaluation of situations elicits emotions, not the characteristics of the situations themselves. This affords a great degree of flexibility in responding to a dynamically changing environment with updated and personalized information. Appraisal theories of emotion are well-supported empirically, though the exact features, boundary conditions, and number of appraisals remain areas of study (Moors 2020). Example appraisals in response to emotion-eliciting situations include: self-relevance (i.e., how much an event matters for the self), motivational congruence (i.e., how consistent is the event with one’s motivation or goals), coping potential (i.e., how matched are one’s resources to those needed for the event), and predictability (i.e., how expected is the event) (Roseman 2013; Scherer 2019). Appraisal theories of emotion contend that different combinations of people’s appraisals reliably converge in response to situations, giving rise to specific emotions (Moors 2014). For example, when an event is seen as highly unpredictable, and other appraisals are less salient, one is likely to feel surprised. Or, when an event is appraised as unexpected, counter to one’s motives, and coping potential is low, one may be likely to experience fear, among other emotions (Roseman 2013; Scherer 2019). 

Although at times people may consciously reflect on appraisals, such as predictability and coping potential, the interaction between appraisals that leads to an emotion is typically automatic and unconscious (Moors 2020). As such, most EU tests require individuals to evaluate social situations—as the situations are thought to be more accessible—assuming the vignettes will tap knowledge of emotion concepts. However, this approach measures emotion knowledge indirectly, and also measures knowledge of situated sociocultural norms that might confound test performance. That said, research suggests emotion appraisals take on a more consciously accessible and identifiable form—which is central to how people think about their emotions—called core relational themes (Campos et al. 2013; Lazarus 1991; Smith and Lazarus 1993; see also Cowen et al. 2019; Shaver et al. 1987).^2^ Core relational themes combine “the individual appraisal components into summaries”, and thus represent “gestalts of relational meaning”, signifying “the central harm or benefit that underlies each of the negative and positive emotions” (Smith and Lazarus 1993, p. 236). Examples of emotions and their core relational themes are: experiencing pride from perceptions of earned achievement, gratitude from perceived generosity, sadness from the perceived loss of something valued, and anger from perceived injustice one experiences or witnesses (see Table 1; Campos et al. 2013; Lazarus 1991; Smith and Lazarus 1993). As with appraisals, relational themes reflect people’s subjective evaluations of situations rather than properties of the situations themselves, and they help people to differentiate among and make sense of their emotions.^3^ Relational themes are reflected on and communicated in daily life as primary meanings of emotions and carry notable psychological and cultural value (Campos et al. 2013; Cordaro et al. 2016a; Lazarus 1991). 

Although there are different perspectives on the relational themes of emotions, many themes arise repeatedly across the literature as central to the meaning of emotions (e.g., achievement, loss; see Table 1; Campos et al. 2013; Cordaro et al. 2016a; Cowen et al. 2019; Lazarus 1991; Smith and Lazarus 1993). Based on these consistencies, relational themes are features of emotions people might reliably identify, and thus the ability to do so could be measured as a facet of EU ability (see Castro et al. 2016; Mayer et al. 2016). Here, we develop and offer initial reliability and validity evidence for the CORE, a test of the ability to categorize and distinguish among core relational themes for 19 different emotions. 

### 1.2. Existing Emotion Understanding Ability Measures and Their Limitations

Currently, there are two primary approaches to assessing EU ability, including understanding emotion appraisals and relational themes: situation-judgment tests (SJTs; measuring knowledge) and performance-based assessments (measuring ability) (Castro et al. 2016). Six EU ability tools have been developed and validated in English for adults that tap the ability to understand emotions, including (in part) emotion appraisals (not core relational themes) (for a review of the EU ability tools see Table 2; see also Castro et al. 2016).^4^ The two most widely used EU ability tests are the Mayer–Salovey–Caruso Emotional Intelligence Test (MSCEIT)-Understanding subtest (Mayer et al. 2002, 2003), and the Situational Test of Emotion Understanding (STEU; MacCann and Roberts 2008). Two more recent measures assess multiple EI abilities in the workplace and contain a subtest tapping EU ability, namely, the Geneva Emotional Competence Test (GECO; Schlegel and Mortillaro 2019) and the North Dakota Emotional Abilities Test (NEAT; Krishnakumar et al. 2016). The group that developed the GECO also published the Geneva Emotion Knowledge Test (GEMOK; Schlegel and Scherer 2018). As the GECO, NEAT, and GEMOK are newer, their validity evidence is limited (see Table 2). We thus benchmark the validity of the CORE with the more established tests (i.e., the MSCEIT-Understanding and STEU).^5^

The MSCEIT helped found the field of ability EI assessment (Fernández-Berrocal and Extremera 2006; Mayer et al. 2008b) and it remains the most cited EI ability measure to date (see Table 2). In the MSCEIT, the Understanding subtest contains two tasks: one assesses how emotions co-occur or blend into each other (Blends Task), and one assesses how emotions may intensify or change over time (Changes Task). To answer the test questions, knowledge of emotion appraisals or relational themes may be helpful, but neither task explicitly measures people’s ability to identify emotion appraisals or core relational themes. Also, the validity evidence for the MSCEIT-Understanding subtest typically combines scores on both tasks, so one cannot discern what facets of EU ability are related to which outcomes (Mayer et al. 2002, 2012; Maul 2012). Another challenge is that the MSCEIT (including the EU subtest) derives its correctness criteria from consensus ratings by emotion experts^6^ and a general population sample (*N* = 5000). There are questions about whether these scoring criteria are optimal for a maximal performance test of EI ability (Fiori et al. 2014; Maul 2012; Miners et al. 2018). Additionally, the MSCEIT validation studies (*N* = 5000), though international, oversampled people under 30 years old with some college education or higher, and race was not representatively sampled (Mayer et al. 2002), potentially limiting test validity only to certain groups (AERA et al. 2014). 

The STEU draws on Roseman’s (2001) emotion appraisal theory and evidence in support of the theory to guide the correctness criteria of the test (MacCann and Roberts 2008). It also assesses emotion appraisals directly (in a subset of items), tests different response formats, and is free of charge. These are noteworthy advances in EU ability testing. That said, the STEU combines scores across different item types, including SJT items set in work and personal life contexts and items meant to tap emotion appraisals directly. This may increase content validity, but the reliance on SJTs with social contexts for most items may add construct-irrelevant variance (AERA et al. 2014) to the test (e.g., measuring of social norms knowledge or cultural rules; e.g., van Rijn and Larrouy-Maestri 2023).^7^ Additionally, the STEU employs binary (correct/incorrect) scoring that may not reflect the extent to which appraisals or relational themes of different emotions meaningfully overlap (see below On the Dimensionality of Emotion), possibly leading to construct underrepresentation (AERA et al. 2014). Finally, as with the MSCEIT, the STEU was validated with primarily White college students or college graduate samples (see Table 2), potentially limiting its validity generalization to those specific samples (AERA et al. 2014).

Existing tests of EU ability signify notable strides in the scientific study of EU. However, the field is still in development, and leading EU tools have limitations, including measurement imprecision, the overreliance on social vignette-based methods, the lack of generalizable validity evidence, and scoring criteria that oversimplify the layered complexity in emotion concepts. Also, no existing tests provide a score quantifying people’s knowledge of core relational themes. Importantly, these limitations are tractable, and we aim to address them (to an extent) with the development and validation of the CORE.

### 1.3. On the Dimensionality of Emotion Space

For decades, researchers have debated the number of distinct emotions, what separates one emotion from another, and how emotion categories vary across different components of emotions (e.g., phenomenology, appraisals, expressive behaviors; Barrett 2017; Barrett and Russell 2014; Ekman 1992; Roseman 2013; Scherer 2019; Smith and Lazarus 1993). Recently, a new research program has utilized massive-scale data collection and machine learning to test the existing theories (Cowen and Keltner 2017, 2021; Keltner et al. 2023). Findings from this work suggest that emotions cluster into “emotion families” based on shared characteristics, including appraisals and relational themes, and that emotion families are related to each other across multidimensional gradients of emotion space (see also Toivonen et al. 2012). Moreover, this perspective supports the notion that though there are primary kinds or clusters of emotions, there is reliable differentiation within emotion clusters, and there appear to be many specific (20+) emotions people distinguish among via facial expressions, vocal tone, music, and language/concepts, where some characteristics are shared and not others. A new, computational theory of emotions was developed based on these findings, called Semantic Space Theory (Cowen and Keltner 2021). 

Semantic Space Theory informed our test development, as it suggests that knowledge about emotion appraisals and relational themes likely exists on a gradient, reflecting *degrees* of semantic relatedness. As such, we adopt a progressive versus dichotomous scoring paradigm (accuracy is determined by degrees of correctness; e.g., Castro et al. 2015) to score our EU ability test. This builds the natural relatedness of emotion themes into the test as signal rather than discarding it as noise. To our knowledge, no other quantitative measures of EU ability adopt this approach and root the correctness criteria in theory and prior research.^8^ Using this approach, we aim to better capture the complexity of EU ability. 

### 1.4. The Present Research

Across three studies, we develop and provide validity evidence for a new performance test of EU ability, called the CORE. We followed best practices in developing and validating new EI ability measures, including clearly defining the theoretical construct and rooting its criterion for correctness in testable theory and prior findings (Maul 2012; Miners et al. 2018). Additionally, we consulted the Standards for Educational and Psychological Testing (AERA et al. 2014) in the process of evaluating the reliability (i.e., internal consistency) and validity evidence of the test, along with our consideration of its guidance regarding test fairness principles and the use of recommended language and terminology. 

Test validity is evaluated based on integrating different kinds of validity evidence guided by the intended test use (AERA et al. 2014). In building the test, we took an expansive view regarding how many emotions exist to increase construct representation, based on recent advances in studying specific emotions (e.g., Cordaro et al. 2016b) and insights into the multidimensionality of emotion space (e.g., Cowen and Keltner 2021) (see Table 1). Regarding the test structure, we examined the test’s unidimensionality with factor analysis. We also gathered convergent and discriminant evidence of validity and examined test-criterion relationships (AERA et al. 2014). Specifically, we studied the CORE’s association with widely used EU tests (i.e., the MSCEIT-Understanding and STEU) (convergent evidence), and the CORE’s relation to more construct-irrelevant variables associated with EU ability, including age, gender, race, education, and to an extent, verbal intelligence (discriminant evidence). For test-criterion relationships, we examined the association between the CORE and three constructs centrally related to EU ability: (1) relational conflict, given the significant role of understanding one’s own and others’ emotions in preventing and navigating social challenges (Brackett et al. 2011); (2) coping, based on the Cascading Model of EI that suggests EU predicts psychosocial and performance outcomes via targeted emotion regulation (Joseph and Newman 2010; Kashdan et al. 2015; see also Castro et al. 2016); and (3) well-being, based on recent data showing the link between EI abilities and indicators of emotional and subjective well-being (Fernández-Berrocal and Extremera 2016; Sánchez-Álvarez et al. 2016). Finally, we examined whether the CORE was associated with outcomes beyond variance accounted for by other EU ability tests (incremental validity evidence). These steps help to clarify measurement precision, measurement versus construct variance, and test-criterion relationships (Maul 2012).

To promote test fairness (AERA et al. 2014), we took three primary steps. First, the CORE items were developed with a demographically diverse sample (Study 1), and the validity data from Studies 2 and 3 also included greater participant diversity than is typical in EU test validation studies (see Table 2). Second, the CORE items were written using brief and simple language, and then we tested whether the items were readable by those with a high school education. Third, in all models examining the CORE’s test-criterion relationships, we included demographic variables to assess the CORE’s validity accounting for the contributions of these factors. We also make recommendations for future research that will help to further examine whether the CORE meets key fairness principles.

## 2. Study 1

The goals of Study 1 were to develop and refine the CORE item pool using a multi-stage process, achieve measurement economy, and assess the initial test reliability (internal consistency). Additionally, to understand participants’ test experiences, we measured their perceptions of test instruction clarity and how engaging they found the test.

### The Development of the Core Relational Themes of Emotion (CORE) Test

To begin, we drew on cognitive appraisal theories of emotions to guide the correctness criteria of the test (Moors 2014, 2020), focusing on core relational themes, which are thought to emerge from primary appraisals (Lazarus 1991; Smith and Lazarus 1993). We chose relational themes to increase the ecological validity of the test. Relational themes are likely a closer approximation of how non-academics consciously think and speak about the meaning of emotions (Smith and Lazarus 1993). We hoped that test-takers would find the relational theme language easier and less confusing to interpret, reducing measurement error. This approach also allowed us to measure emotion knowledge more directly rather than inferring it from responses to widely used situation-based vignettes.

Next, we selected 24 emotions with empirically supported core relational themes from three literatures: (i) relational theme studies (e.g., Lazarus 1991) and appraisal theories (e.g., Roseman 2013); (ii) recent large-scale empirical studies on the dimensionality of emotion (e.g., Cowen and Keltner 2017, 2021); and (iii) studies of specific emotions (e.g., pride research by Tracy and Robins 2007). The emotions were: amusement, awe, compassion, contentment, gratitude, hope, inspiration, interest, joy, love, pride, relief, anger, anxiety, boredom, disgust, embarrassment, envy, fear, guilt, jealousy, sadness, shame, and surprise. We examined whether multiple themes were present per emotion and considered all core themes for each emotion to serve as the basis for test items. The relational themes we used to write the items and to determine response accuracy are in Table 1. 

We drew on the specific language describing the core relational themes from the literature to write the CORE items. In doing so, we included common phrases used by researchers and participants to describe relational themes. We adapted words and phrases as needed to ensure the use of simple and plain language. We wanted those with a high school education to understand the items (noting that other EU ability tools largely develop and validate their items with college-educated samples; see Table 2). We wrote and revised items in an iterative cycle to capture the relational themes as succinctly as possible. 

Then, an expert panel of five doctoral-level emotion scientists (the authors) with backgrounds in psychology, organizational behavior, and education reviewed the items and answers (derived from theory and prior work). Panelists possessed relevant research knowledge about emotion, cognition, and EI abilities and EI tests (AERA et al. 2014). The panel reviewed the items, assessing: (1) item accuracy—fidelity to the emotion science literature for each relational theme; (2) item diversity—coverage of emotions across the breadth of emotion space, including positively and negatively valenced and high and low arousal emotions; (3) item differentiation—a reasonable degree of exclusivity between relational themes within and between emotions to distinguish among them (noting full mutual exclusivity was not possible given our theoretical orientation toward Semantic Space Theory; Cowen and Keltner 2021); and (4) item readability—clarity and concision in item language. Panelists reviewed the items on their own, and then met as a group to discuss the extent to which items met the criteria. Item framing, word choices, and answer decisions, among other topics, were deliberated until the panel agreed on sufficient satisfaction of all criteria, including changing items and adding or dropping items. Following these steps, we developed 78 items for inclusion in the CORE to be tested in Study 1. 

## 3. Materials and Methods

### 3.1. Participants and Procedure

Psychological test development standards suggest that subgroups relevant to the intended test use should be employed in test construction (AERA et al. 2014). Accordingly, for the creation of the CORE in Study 1, we implemented disproportionate stratified sampling using equal allocation to obtain equal representation across major demographic groups in U.S. adults (our target population) (see Daniel 2012). This approach also allowed us to build a confusion matrix (see below). Specifically, we aimed to sample the following demographic characteristics equally reported by the U.S. Census Bureau (2020a, 2020b, 2020c): age, gender, race, and education level. We did not nest our sampling targets within each other (e.g., an equal number of men and women across racial categories), given practical constraints. Conducting such a study in future research would be useful, as it would permit an examination of the role intersectionality plays in EU ability and EU tests, as measured by the CORE and other EU ability tools (e.g., see Monroy et al. 2022). The sampling targets were as follows: (i) 33% ages 18–29, 33% ages 30–49, and 33% ages 50–65; (ii) 50% female and 50% male; (iii) 25% Asian, 25% Black, 25% Latinx, 25% White^9^; (iv) 33% high school education, 33% some college or associate degree, and 33% four-year college degree or higher. The obtained sample (*N* = 684) largely reflects these targets with a degree of under/oversampling (see Table S2). Regarding age, participants were 26.8% 18–29, 47.3% 30–49, and 25.9% 50–65. In terms of gender and race, participants were 55.7% female, and 38% White, 22.5% Latinx, 19.9% Asian, and 19.6% Black. Regarding education, 28.8% had a high school education, 31.4% reported some college or an associate degree, and had 39.8% four-year college degree or higher. Also, 100% of participants were primary English language speakers and 100% worked full-time (>30 hours a week) across sectors (e.g., education/research, construction/manufacturing, and business/finance).

Participants were recruited via Qualtrics panel services and they were financially compensated for their time. This study was administered online via the Qualtrics platform in July of 2020. Two attention checks were included in the study (one or more attention checks missed was considered grounds for response removal; Kung et al. 2018). Also, completion time was reviewed to ensure data quality (finishing the study in less than ¼ median time was considered speeding; Curran 2016). Screening was implemented proactively, so responses that did not meet our requirements were automatically screened out. Informed consent was obtained from all participants involved in this study. The research study was reviewed and approved by our university IRB (protocol #: 2000022943).

### 3.2. Analytic Plan

#### 3.2.1. Confusion Matrix, Item Pruning, and Progressive Scoring

We calculated the proportion of participants who chose the target answer for each of the 78 items (i.e., the raw “hit rate”). We then generated a confusion matrix (see Tables S3–S6). A confusion matrix indicates the proportion of participants who selected the target response on the diagonal of the matrix (i.e., how often they picked the target response), and the proportion of participants that selected any “distractor” responses on the off-diagonal (e.g., LaPalme et al. 2023; Laukka et al. 2016). To aid interpretability, we converted raw hit rates into a proportion index based on the total number of response options (i.e., 24 possible responses/emotions). This proportion index (*pi*; Hall et al. 2008; Rosenthal and Rubin 1989) represents hit rates as if the answers were made dichotomously (though they were not).^10^ Chance level of accuracy is .50 (see the Supplemental Materials).

Along with applying the same criteria from the expert panel (i.e., item accuracy, diversity, differentiation, and readability), we used the confusion matrix results to prune CORE items. The goal was to increase measurement economy while retaining key facets of test reliability and validity. Our plan was to remove items where the hit rate was below chance (.50) or nearly perfect (1.00), and then to retain items that covered as much emotion space as possible, trying to include at least two items per emotion. We also anticipated the removal of emotions and items where there was high semantic redundancy.

Full credit, half credit, and no credit (distractor) responses were based on: (i) relational theme (e.g., Lazarus 1991) and appraisal theory research (e.g., Roseman 2013); (ii) recent findings on the semantic relatedness of emotion concepts (e.g., Cowen et al. 2019; Toivonen et al. 2012); and (iii) research programs on specific emotions (e.g., Tracy and Robins 2007) (see Table 1). The confusion matrix provided additional information to consider when finalizing the scoring key and it was a direct empirical test of the overlap between relational themes identified from the literature for the 24 target emotions. Emotions within the same emotion family, but not the target answer, were assigned half credit, such as: gratitude for a love item (prosocial emotion family), jealousy for an envy item (self-conscious emotion family), and inspiration for an awe item (epistemological emotion family) (Sauter 2017; Shiota et al. 2014; Simon-Thomas et al. 2009). Emotion families are linked by the evolutionarily adaptive and primary psychosocial functions they serve (e.g., see Keltner et al. 2022). This scoring approach is aligned with studies that suggest emotions exist along multidimensional gradients connected by clusters that share core meanings and functions, noting that substantive distinctions between emotions within the same emotion family can be made (Cowen et al. 2019; Cowen and Keltner 2021). For distractors, we selected emotions with potential semantic overlap, higher hit rates than other incorrect answers, and similar valence and arousal levels to the target answer (e.g., interested, amused, content for an inspired item, or embarrassed, guilty, anxious for a jealousy item).

#### 3.2.2. Reliability

We used Cronbach’s alpha to test the internal consistency of the CORE and all other measures in the present research (Kalkbrenner 2023). We report this form of reliability as we expected that test scores are stable over time, given the consistency in EI and EU ability across test administrations (without EI training; see Mayer et al. 2003) (AERA et al. 2014). 

#### 3.2.3. Participant Ratings of Instruction Clarity and Test Engagement

Participants rated the CORE instructions as 1 (clear), 2 (confusing), or 3 (other; text entry). We also asked respondents to rate all test items on a scale of 1 (interesting/engaging) to 7 (dull/tedious).^11^ We calculated response percentages to assess ratings of instruction clarity and used mean scores to assess how engaging participants found the test.

## 4. Results

### 4.1. Confusion Matrix

The confusion matrix indicated that most participants selected the target answer on the test items above 0.50 (chance). Across all 78 items, the mean *pi* (i.e., chance-adjusted hit rate) was 0.94 (*SD* = 0.04). The item-level hit rate range was 0.73 to 0.98. At the emotion level, the lowest and highest chance-adjusted hit rates were for guilty (0.86) and surprise (0.98), respectively (see Tables S3–S6). High hit rates may reflect shared knowledge of relational themes and their intuitive nature (Smith and Lazarus 1993), and the simple item language and direct measurement of emotion knowledge. That said, the raw hit rates clearly indicate that many participants still found the items hard to answer correctly. Without adjusting for chance, the mean raw hit rate (percent correct) was 0.46 (*SD* = 0.11). The item-level raw hit rate range was 0.15 to 0.78, and at the emotion level, the lowest and highest raw hit rates were for guilty (0.23) and surprise (0.68), respectively (see Tables S3–S6). 

### 4.2. Item Pruning

As the hit rate for all items was above chance (0.50), we did not use the <0.50 cutoff to prune CORE items. We still employed high hit rates (close to 1.00) to aid item pruning to increase item-level difficulty across emotions and the test. We also explored how often a non-target emotion was selected with a comparatively high hit rate (>0.70) to identify emotion overlap. That said, we first removed emotions from the CORE that did not add unique information, while retaining as many emotions with distinct relational themes as possible. We also tried to keep emotions that were of positive and negative valence, and high and low arousal to increase content validity. From this process, we removed all items for five emotions: interest, relief, fear, guilt, and surprise. The interest items were pruned as the relational theme was broad (i.e., novelty; Silvia 2005), overlapped highly with other emotions (e.g., surprise; Lazarus 1991), and other high-energy positive emotions were represented (e.g., pride). Similarly, the items for guilt overlapped too heavily in semantic features with shame, as did the answers for fear with anxiety.^12^ Relief and surprise each only had one core relational theme (see Table 1) and they were easy to answer (emotion-level chance-adjusted hit rates = 0.96 for relief and 0.98 for surprise). We therefore retained items for 19 emotions. Next, we removed items within the 19 emotions that were different ways of capturing the same relational theme to offer coverage of multiple themes for each emotion (where possible). Finally, when multiple relational themes were present, we relied on the literature to select the two most empirically supported themes. After pruning, the CORE consisted of 38 items total, covering 19 different emotions with two items each. 

### 4.3. Progressive (Degrees of Correctness) Scoring

With the 38-item set, we implemented a progressive (degrees of correctness) test scoring paradigm (e.g., Castro et al. 2015). Rather than use a dichotomous approach, where answers are only correct or incorrect, participants can receive 0 points (no credit), 0.5 points (half credit), or 1 point (full credit) (the scoring key is in Table S7). A higher score is intended to reflect a greater understanding of the core relational themes of emotions. Other EI ability tests have utilized progressive approaches for scoring protocols (e.g., Castro et al. 2015). Yet, to our knowledge, no other tests of EU ability have used progressive scoring methods. Theory and past findings mainly converged with the confusion matrix results regarding the full and half credit answers (mean chance-adjusted hit rate for the half-credit answers = 0.70, *SD* = 0.17). Answers that were not the target response were not random. The confusion matrix indicated participants selected non-target answers (e.g., jealousy for an envy item; fear for an anxiety item) above chance for 33 of 38 items. These answers appear to reflect the continuous gradients of shared meaning that emotions vary along (Cowen and Keltner 2021). No credit distractors were selected based on their semantic proximity to the correct answers, hit rates, and valence and arousal properties.

### 4.4. Reliability 

Reliability (internal consistency) was high in the unpruned 78-item (α = 0.94) and pruned 38-item (α = 0.90) CORE. We retained the 38-item test for the sake of test economy.

### 4.5. Participant Experiences of the CORE 

We found that 94.2% of participants indicated the instructions were “clear”, 4% found them “confusing”, and 1.8% selected “other”. In terms of test engagement, participants gave the CORE a mean rating of 5.73 (*SD* = 1.91) out of 7. For the most part, participants understood the test instructions and found the test moderately interesting and engaging.

### 4.6. Readability Statistics of the CORE

We calculated the commonly used Flesch–Kincaid Test and Gunning Fog Index to determine the readability of the CORE items. The Flesch–Kincaid Test calculates reading difficulty using average sentence length and average word length. The Gunning Fog Index calculates average sentence length and percent of complex words (words with three or more syllables). The CORE had a Flesch–Kincaid score of 6.7 out of 18, indicating it is readable for people at a sixth to seventh grade reading level or higher. The CORE had a Gunning Fog score of 10.14 out of 20, indicating it is readable for people at a tenth to eleventh grade reading level or higher. Our goal was for the CORE items to be readable for individuals with a high school education, and the CORE meets this benchmark.

## 5. Discussion 

In Study 1, we reviewed multiple literatures in emotion science, selected core relational themes for 24 emotions, wrote 78 test items, and had an expert panel evaluate the items. We used emotion theory, prior findings, and the results from a confusion matrix to prune items and develop the progressive scoring key. The CORE showed high internal consistency, participants rated the test instructions as clear and the test as moderately engaging, and the items were readable by those with a high school education. The 38-item CORE (covering 19 different emotions) and answer key are in the Supplemental Materials. 

## 6. Study 2

In Study 2, we examined the factor structure of the CORE. We also studied its construct validity by testing for convergent relationships between the CORE and widely used measures of EU ability (i.e., MSCEIT and STEU), and discriminant relationships between the CORE and demographic characteristics (i.e., age, gender, race, education level) and (to some extent) verbal intelligence (AERA et al. 2014).^13^ Additionally, we included a preliminary measure of test-criterion relationships (i.e., relational conflict). 

Based on theories that contend EU ability is multi-faceted, we hypothesized understanding relational themes specifically to constitute one such facet of EU ability (Castro et al. 2016; Mayer et al. 2016). We thus predicted a single-factor structure would best fit the CORE. Also, based on research examining the construct and criterion-related correlates of other EU ability measures (Joseph and Newman 2010; MacCann and Roberts 2008; Mayer et al. 1999, 2003, 2008a, 2008b; Schlegel and Mortillaro 2019; Schlegel and Scherer 2018), we predicted: (i) the CORE to show moderate to large positive correlations with existing EU ability measures; (ii) small to moderate positive correlations with age, female gender, and education level; (iii) a moderate to large positive correlation with verbal intelligence; (iv) and a small to moderate negative correlation with relational conflict frequency. We did not predict how the CORE would relate to race, given the limited evidence on this topic. We selected relationship conflict as a preliminary criterion outcome based on prior research linking EI abilities to relationship quality and challenges (Brackett et al. 2005; Kotsou et al. 2019; Lopes et al. 2003, 2004), and research suggesting that how skillfully people process their emotions plays a central role in their relationship satisfaction and outcomes (e.g., see Sbarra and Coan 2018). For effect sizes, we used Cohen’s (1988, 1992) conventions: “small” *r* = 0.10–0.29, “medium” *r* = 0.30–0.49, and “large” *r* = 0.50 or greater. 

## 7. Materials and Methods

### 7.1. Participants and Procedure

We aimed to representatively sample the U.S. working population in Study 2 to generalize the results to this group (AERA et al. 2014). The sampling targets were: (i) 100% age 18 or older; (ii) 47% female and 53% male; (iii) 70% White, 15% Latinx, 10% Black, 5% Asian; and (iv) 33% high school education, 27% some college or associate degree, and 40% four-year college degree or higher (U.S. BLS 2020). The collected sample (*N* = 284) largely reflects this distribution (see Table S8). Participants were all above age 18 (*M* age = 41.2 years, *SD* = 14.2), 50.4% female, and 66.2% White, 14.8% Latinx, 12.3% Black, 6.7% Asian. The education breakdown was: 27.1% high school education, 26.7% some college or associate degree, and 46.1% four-year college degree or higher. Also, participants were 100% primary English language speakers, and 100% worked full-time (>30 h a week) across multiple industries (e.g., business or finance, construction or manufacturing, and the service sector). We marginally oversampled female (by 3%), Black (by 2.3%), Asian (by 1.7%), and four-year college graduate (or higher) participants (by 6%), and undersampled high school educated participants (by 6%), so we consider this sample “quasi-representative”.

Recruitment occurred utilizing Qualtrics panel services, and participants were paid for their study time. The measures were administered online using the Qualtrics website in July of 2020. We used the same attention and speeding check procedures to ensure data quality as Study 1. Responses were culled proactively as they came in, and so the full sample was retained. Informed consent was obtained from all participants involved in this study, and the study was approved by our university IRB (protocol #: 2000022943). 

### 7.2. Measures 

The CORE. We administered the 38-item CORE developed in Study 1. The CORE assesses people’s ability to identify the core relational themes of 19 different emotions. Participants select from five response options. The test showed high reliability (α = 0.94). 

MSCEIT-Understanding Subtest. The EU subtest of the Mayer–Salovey–Caruso Emotional Intelligence Test (MSCEIT; Mayer et al. 2002, 2003) is a measure of EU ability. It contains two parts: the Blends and the Changes Tasks. The Blends Task contains 12 items where participants either combine emotions into more complex ones or dissect a complex emotion into its component parts. The Changes Task is a 20-item task where participants analyze how emotions transition and change in intensity over time. Both tasks use a five-option multiple choice format. The EU subtest showed good reliability (α = 0.84). 

STEU. The Situational Test of Emotion Understanding (STEU; MacCann and Roberts 2008) is a 42-item EU ability test. Respondents read vignettes and select the emotion that best fits how a person may feel using multiple choice. STEU reliability was good (α = 0.84). 

Verbal Intelligence. Verbal intelligence was measured using the Wordsumplus Test (Cor et al. 2012). This is a 14-item test where participants indicate the word that is closest in meaning to the target word. The scale has six options to select from, including a “don’t know” option (marked as incorrect). The scale showed good reliability as well (α = 0.81).

Relational Conflict. Relational conflict was assessed with items from the Network of Relationships Inventory-Relationship Qualities Version (NRI-RQV; Buhrmester and Furman 2008). Respondents indicated their frequency of conflict with friends, family, and romantic partners from 1 (never) to 6 (constantly). Scale reliability was good (α = 0.88).

### 7.3. Analytic Plan 

#### 7.3.1. Confirmatory Factor Analyses

To assess the single-factor structure of the CORE, we used confirmatory factor analysis (CFA) with the weighted least squares mean values (WLSMV) estimator in Mplus 8.1. WLSMV is preferred for CFAs with categorical factor indicators (Li 2016). We tested model fit using the comparative fit index (CFI), the root mean square error of approximation (RMSEA), and standardized root mean squared residual (SRMR). Our benchmarks for “adequate fit” were: ≥0.90 for CFI and ≤0.08 for RMSEA and SRMR, and for “good fit” were: ≥0.95 for CFI and ≤0.05 for RMSEA and SRMR (Hooper et al. 2008; Hu and Bentler 1999). Standardized factor loadings exceeding 0.40 were considered acceptable.

We also conducted single-factor CFAs of the MSCEIT-Understanding, STEU, verbal intelligence measures, and relational conflict scale. We used the same analytic approach as we did for the CORE, except we used maximum likelihood estimation with robust standard errors (MLR) in Mplus 8.1 for the CFA of relational conflict (as the factor indicators were continuous; Li 2016). We saved the latent factor scores of each measure for use in all subsequent analyses. Factor scores incorporate item-level variance into latent variables that increases information in the model, as some items may (and frequently do) contribute more to the total score or carry more error than other items (Bollen 2002; McNeish and Wolf 2020). Employing factor scores versus manifest means thus more accurately estimates measurement error and increases power to detect effects (Rdz-Navarro 2019).^14^

#### 7.3.2. Convergent and Discriminant Evidence, and Test-Criterion Relationships

To assess convergent and discriminant validity, and test-criterion relationships, we entered the factor scores of the CORE and other measures into bivariate correlations in SPSS 28.0. Gender (male = 0, female = 1), race (White = 0, POC^15^ = 1), and education level (less than four-year college degree = 0, 1 = four-year college degree or higher) were dichotomized given their distributions, and we correlated these variables with the CORE. Also, we ran partial correlations between the CORE with other EU ability tests, adjusting for verbal intelligence. All EU ability tools share sizeable variance with verbal intelligence (Joseph and Newman 2010; Mayer et al. 2008a, 2008b), and so this helped to precisely evaluate evidence of the CORE’s convergence with other established EU ability tests.

#### 7.3.3. Incremental Validity

Lastly, we examined whether the CORE accounted for additional variance in the criterion-related outcome (i.e., relational conflict), while accounting for variance from demographic factors and widely used EU ability tools. To test incremental validity, we conducted multiple regression analyses. In the first block, we entered demographic variables (i.e., age, gender, race, and education). In the second block, we entered either the MSCEIT-Understanding subtest or the STEU.^16^ In the third block, we entered the CORE. Utilizing this stepwise process, we examined whether the *R*^2^ value significantly increased when adding the CORE to the model, compared to the model with only demographics and other EU ability tests. We also inspected whether the effect remained significant for the CORE and whether it became non-significant for the MSCEIT and STEU in the third block.

## 8. Results

### 8.1. Test Completion Time 

The mean completion time of the CORE was 6.97 (*SD* = 4.48) minutes. Though we screened out speeders, we did not remove participants for taking “too long” to complete the study. As such, the median may offer a more accurate estimate at 5.55 minutes. Either way, the test takes approximately 5.5 to 7 minutes to complete, supporting test economy.

### 8.2. Factor Structure: CFA

A one-factor CFA of the CORE showed a good model fit, supporting our prediction, *X*^2^(665) = 726.80, *p* = .05; RMSEA = 0.02; CFI = 0.99; SRMR = 0.06. Standardized factor loadings were moderate to high and ranged from 0.40 to 0.87 (all loadings are presented in Table S9).

### 8.3. Construct Validity Evidence: Latent Variable Correlations 

Using factor scores, the CORE showed large positive associations with the MSCEIT-Understanding subtest (*r* = 0.82, *p* < .001) and the STEU (*r* = 0.85, *p* < .001) (see Table 3). The CORE, MSCEIT-Understanding, and STEU all showed large, commensurate relations to verbal intelligence (*r*s = 0.66, 0.66, and 0.67, *p*s < .001, respectively). Though the 0.66 to 0.67 relationships between the CORE, MSCEIT-Understanding, and STEU with verbal intelligence are sizeable (about 45% of the variance in EU overlaps with verbal intelligence)—over half (55%) of the variance in EU ability is not accounted for by verbal intelligence. Adjusting for verbal intelligence, the partial correlations between the CORE and the MSCEIT-Understanding (*r* = 0.70, *p* < .001) and STEU (*r* = 0.73, *p* < .001) decreased but remained large.

The CORE showed a moderate positive correlation with age (*r* = 0.30, *p* < .001), a small positive correlation with female gender (*r* = 0.17, *p* < .01), no correlation with race (*r* = −0.04, *p* = .50), and a moderate negative correlation with education (*r* = −0.25, *p* < .001; see Table 3). The results are consistent with prior work (Mayer et al. 2008a, 2008b), though a negative link with education is atypical (the MSCEIT and STEU showed the same pattern).

### 8.4. Initial Evidence of Test-Criterion Relationships and Incremental Validity 

Regarding test-criterion relationships, the CORE latent factor score showed a moderate to large negative association with relational conflict (*r* = −0.42, *p* < .001; see Table 3).

Regarding incremental validity, adding the CORE to a multiple regression model containing demographic covariates and MSCEIT-Understanding, produced a significant increase in the *R*^2^, *R*^2^ = 0.18, *F*(6,132) = 4.67, *p* < .001. The *R*^2 change^ (132) = 0.05, *p* < .01, and total adjusted *R*^2^ = 0.14. Also, after adding the CORE, the MSCEIT-Understanding link with relational conflict became non-significant (from *β* = −0.24, *p* = .01 without to *β* = 0.06, *p* = .68 with the CORE), while the CORE relationship remained significant (*β* = −0.40, *p* = .01).

Adding the CORE to a multiple regression model containing demographics and the STEU produced an increase in the *R*^2^, *R*^2^ = 0.26, *F*(6,130) = 7.62, *p* < .001. The *R*^2 change^ (130) = 0.02, *p* = .04, and the total adjusted *R*^2^ = 0.23. Adding the CORE to the model, the STEU link with relational conflict became non-significant (from *β* = −0.35, *p* < .001 without to *β* = −0.09, *p* = .58 with the CORE), while the CORE association remained (*β* = −0.30, *p* = .04).^17^

Finally, we tested whether the CORE was associated with relational conflict, accounting demographic variables and verbal intelligence. Adding the CORE to a multiple regression model containing demographics and verbal intelligence scores produced an increased *R*^2^, *R*^2^ = 0.20, *F*(6,269) = 11.52 *p* < .001. The *R*^2 change^ (269) = 0.06, *p* < .001, and the total model adjusted *R*^2^ = 0.19. The CORE remained negatively associated with relational conflict (*β* = −0.35, *p* < .001), providing evidence of a test-criterion relationship between the CORE and relational conflict beyond shared variance with demographics and verbal intelligence.

## 9. Discussion

In Study 2, the CORE showed high reliability and the predicted unidimensional factor structure was well-supported. We also found evidence of convergence between the CORE with widely used EU ability tests, and evidence of divergence between the CORE with demographics and verbal intelligence, supporting its construct validity (AERA et al. 2014; Cronbach and Meehl 1955; Smith 2005). The CORE’s association with verbal intelligence is akin to other EU ability tests in prior studies (Joseph and Newman 2010; Mayer et al. 2008a, 2008b), and in our data, with the MSCEIT-Understanding and STEU. Notably, the CORE was associated with the MSCEIT-Understanding and STEU beyond shared variance with verbal intelligence, suggesting the CORE measures EU ability, independent of verbal ability. Finally, the CORE was associated with less relationship conflict, accounting for demographics and established EU ability tests, supporting its incremental validity.

## 10. Study 3

The goals of Study 3 were to further examine the test-criterion relationships and incremental validity of the CORE in comparison with a widely used EU ability measure (the STEU^18^). We sampled professionals working in education, as emotion abilities may be particularly useful for populations engaging in high emotional labor (Newman et al. 2010), including education professionals (Wang et al. 2019). We investigated coping and well-being as outcomes based on the Cascading Model of EI (Joseph and Newman 2010), which specifies that EU ability predicts psychosocial and performance outcomes via emotion regulation. Also, recent findings show a link between EI abilities, coping, and well-being, and support the Cascading Model (Fernández-Berrocal and Extremera 2016; Sánchez-Álvarez et al. 2016). Specific outcomes selected reflect the multidimensionality of coping (e.g., Carver 1997) and well-being (e.g., Diener 2009; Lyubomirsky 2008; Ryff and Singer 2008; Seligman 2011), and they tap the demands of working in education (Granziera et al. 2021; Travers 2017). Broadly, coping skills are ways people manage emotional challenges, and effective coping means engaging in typically helpful (“adaptive”) and disengaging from typically unhelpful (“maladaptive”) coping strategies, where helpful and unhelpful strategies are determined by which reliably support well-being (e.g., Webb et al. 2012).^19^ Well-being includes positive (e.g., job satisfaction) and negative emotional experiences at work (e.g., emotional exhaustion), social-emotional demands (e.g., emotional labor and compassion fatigue), along with eudaimonia (e.g., a sense of purpose) and mindsets about emotions (e.g., implicit theories about emotion malleability) (Madigan and Kim 2021; Page and Vella-Brodrick 2009). Based on prior research (Fernández-Berrocal and Extremera 2016; Kotsou et al. 2019; Sánchez-Álvarez et al. 2016), we predicted the CORE would be positively related to adaptive coping, job satisfaction, purpose, and a mindset where emotions are seen as malleable and can be changed (Tamir et al. 2007). We also predicted that the CORE would be negatively related to maladaptive coping, emotional exhaustion, emotional labor, and compassion fatigue. We expected all effects to be small (*β* > 0.20) to medium (*β* = 0.20–0.49) in size (Fey et al. 2023; Joseph and Newman 2010). Lastly, we predicted that these results would hold with participant demographics and STEU scores in the same model, suggesting that the CORE accounts for unique variance in these outcomes.

## 11. Materials and Methods

### 11.1. Participants and Procedure

The total sample was *N* = 491 (see Table S11). Noting that the largest single race represented was White participants (39.3%), POC-identifying individuals comprised 60.5% of the sample (see below). The mean age was 39.0 (*SD* = 8.3). The majority of participants (69.1%) were employed full-time at a preK-12 school with an average of 11.0 years working in education (*SD* = 7.2). Modal income ranged from $50,000 to $59,999 a year, and the modal education level was a master’s degree (45.0%).^20^ Many participants (52.2%) reported working both remotely and in-person. The remaining participants reported only remote/virtual work (37.6%), only in-person (8.9%), or “other” work modality (1.3%). 

We collaborated with seven national and regional organizations that represent Black and Latinx educators in the U.S. who supported study recruitment and outreach. We oversampled Black (28.9%) and Latinx (28.0%) educators to more equitably represent educators of color in research in the field. We disseminated the study via educational newsletters, listservs, talks and events, and educators’ social media for a study on educator well-being. Participants were also able to share the study link with colleagues. The study took place online using Qualtrics and lasted about 20–25 minutes. The data reported for this study are a substudy conducted within a larger national study on educator coping and well-being. Participants were paid for their time. Informed consent was obtained from all participants. This research was approved by our university IRB (protocol #: 2000029065). 

### 11.2. Data Screening

We used the same screening criteria as Studies 1 and 2, though they were not applied proactively. After inspection, *n* = 30 participants missed at least one attention check and/or were categorized as speeding. All results reported use the screened sample (*n* = 461).

### 11.3. Measures

The CORE. We used the same CORE as Study 2, and it was highly reliable (α = 0.96). 

STEU-B. The Situation Test of Emotion Understanding-Brief (STEU-B; Allen et al. 2014) is a 19-item version of the 42-item STEU. STEU-B reliability was good: α = 0.83.^21^

Adaptive and Maladaptive Coping. To measure coping economically, we selected single items from the 14 coping strategies on the Brief-COPE (Carver 1997). The extent to which coping strategies are considered adaptive or maladaptive may vary by person, context, and culture (e.g., Bonanno and Burton 2013; Matsumoto et al. 2008). That said, based on research examining which coping strategies tend to correlate with beneficial outcomes (e.g., Carver and Vargas 2011; Webb et al. 2012), we considered the following strategies adaptive: acceptance, problem solving, positive reappraisal, planning, emotional support, instrumental support, humor, and religion. From past studies on which strategies tend to correlate with undesirable outcomes (e.g., Carver and Vargas 2011; Webb et al. 2012), we considered the following strategies maladaptive: distraction, denial, behavioral disengagement, venting, self-blame, and substance use. The response scale was 1 (didn’t do this at all) to 5 (did this almost all of the time). The reliability of the adaptive (α = 0.74) and maladaptive (α = 0.68) coping measures was acceptable. A two-factor CFA of our coping model was supported adequately by the data (see the Supplemental Materials).^22^

Emotional Exhaustion. We used the seven-item Emotional Exhaustion subscale of the Maslach Burnout Inventory for Educators (MBI-ES; Maslach et al. 1996). The response scale ranged from 1 (never) to 7 (every day). Scale reliability was high: α = 0.92. 

Job Satisfaction. Job satisfaction was measured using three items from the Teaching Empowering Leading Learning (TELL) Survey (New Teacher Center 2017). The response scale was 1 (completely disagree) to 6 (completely agree). The scale was reliable: α = 0.84. 

Emotional Labor. The authors developed a brief, face-valid scale to assess emotional labor for educators rather than use a general scale to enhance ecological validity. Three items were generated by an educational researcher and an emotion scientist. Items focused on up-regulating positive emotions while experiencing negative emotions, as this is a common emotional labor demand (see Grandey and Gabriel 2015; Wang et al. 2019). The items were “At work… I feel I have to seem happy to students, coworkers, and others, even when I’m feeling depleted; Show enthusiasm to students, coworkers, and others, even when I’m feeling down; Look calm to students, coworkers, and others, even when I’m feeling anxious”. The response scale ranged from 1 (strongly disagree) to 5 (strongly agree). A CFA supported a single-factor structure for this measure (see the Supplemental Materials). The scale reliability was acceptable: α = 0.76. 

Compassion Fatigue. Compassion fatigue was measured using five items from the Compassion Satisfaction and Fatigue scale (CSF; Figley 1995; Stamm 2002). We selected items that fit the education work environment and emotional demands. The response scale ranged from 1 (never) to 6 (very often). A single-factor CFA of this measure was supported by the data (see the Supplemental Materials). Scale reliability was good: α = 0.87.

Meaning and Purpose. We used the PROMIS Meaning and Purpose Short-Form measure (Salsman et al. 2020) to assess sense of purpose. The response scale ranged from 1 (strongly disagree) to 5 (strongly agree). The scale reliability was acceptable: α = 0.79. 

Malleable Emotion Mindset. We measured implicit theories of emotion—which we call “malleable emotion mindset” for clarity—using a version of Tamir et al.’s (2007) four-item scale. Participants rated statements regarding their beliefs about the malleable versus fixed nature of emotions. The version we used changed items to “I” statements, rather than rating people in general, to increase predictive validity (Castella et al. 2013). Responses ranged from 1 (strongly disagree) to 5 (strongly agree). An example item is “If I want to, I can change the emotions that I have”. Scale reliability was acceptable: α = 0.68. 

### 11.4. Analytic Plan 

#### 11.4.1. CFAs

We conducted CFAs of the CORE, STEU-B, and all eight outcomes. We saved those factor scores, and then used them in all analyses reported below to reduce measurement error and to increase power in our statistical models (Rdz-Navarro 2019).^23^ We used the WLSMV estimator for CFAs of the CORE and STEU-B, and the MLR estimator for all outcome variables in Mplus (Li 2016), utilizing the same model fit criteria as Study 2. 

#### 11.4.2. Evidence of Test-Criterion Relationships and Incremental Validity

We first ran bivariate correlations between the CORE, STEU-B, eight outcomes, and the covariates (i.e., age, gender, race, income, education) in SPSS 28.0. Gender (male = 0, female = 1), race (White = 0, POC = 1), and education (less than four-year college degree = 0, 1 = four-year college degree or higher) were dichotomized to reduce model parameters.

Next, we conducted multiple regression analyses, where demographic covariates were entered in the first block, and the CORE was entered in the second block with the coping and well-being variables entered as outcomes. We ran separate regressions for each outcome given the intercorrelations between the outcomes (see Table 4). 

To test incremental validity, we conducted multiple regression models where demographic factors were entered in the first block, the STEU-B was in the second block, and the CORE was in the third block, with coping and well-being as outcomes. This allowed us to test whether the CORE was associated with outcomes accounting for variance from the demographics and the STEU-B. We looked for changes in the *R*^2^ from the second to third block, and whether the CORE and STEU-B effects were significant in the third block.

## 12. Results 

### 12.1. Demographic Correlations

Like Study 2, age (*r* = 0.34, *p* < .001) and female gender (*r* = 0.34, *p* < .001) were positively associated with CORE performance (see Table 4). Unlike Study 2, an inverse correlation between POC identity and CORE performance was found (*r* = −0.64, *p* < .001), and education level also was positively associated with CORE scores (*r* = 0.45, *p* < .001). The income–CORE association was small (*r* = 0.10, *p* < .05). Overall, these effects are similar to those found with other EI ability tests, noting there is limited research on the role race plays in EI abilities and EU ability specifically (Joseph and Newman 2010; Mayer et al. 2008a, 2008b).

### 12.2. Evidence of Test-Criterion Relationships

The multiple regression analyses, including demographic covariates in the model, indicated that the CORE was positively associated with adaptive coping (*β* = 0.18, *p* < .01), job satisfaction (*β* = 0.24, *p* < .001), meaning and purpose (*β* = 0.40, *p* < .001), and a malleable emotion mindset (*β* = 0.43, *p* < .001) (see Table 4 for zero-order correlations). Also, the CORE was negatively associated with maladaptive coping (*β* = −0.46, *p* < .001) and compassion fatigue (*β* = −0.38, *p* < .001). Counter to prediction, the CORE was unrelated to emotional exhaustion (*β* = −0.04), and it was positively related to emotional labor (*β* = 0.27, *p* < .001).

### 12.3. Incremental Validity 

Adding the CORE to a multiple regression model with demographics and the STEU-B produced a significant *R*^2^ increase for five of eight outcomes (see Table 5 and Table 6). Supporting incremental validity, the CORE remained associated in expected directions with meaning and purpose (*β* = 0.41, *p* < .001), a malleable emotion mindset (*β* = 0.54, *p* < .001), maladaptive coping (*β* = −0.39, *p* < .001), and compassion fatigue (*β* = −0.37, *p* < .001), with demographics and the STEU-B in the same model.^24^ The effect for emotional labor remained significant (in the inverse direction of prediction) for the CORE (*β* = 0.22, *p* = .04) and the STEU-B (*β* = 0.33, *p* < .01) (reasons for this are offered in the General Discussion). In contrast, with the CORE included in the model, five significant STEU-B associations with the outcomes became non-significant and one association decreased (see Table 5 and Table 6).

## 13. Discussion

In Study 3, demographic factors (age, gender, race, education) were associated with the CORE, largely in expected directions based on prior research (Joseph and Newman 2010; Mayer et al. 2008a, 2008b), noting POC identity showed an inverse relationship. The CORE also was moderately associated with theoretically relevant outcomes, including healthy coping and multiple indicators of well-being, accounting for demographic factors. These results are consistent with our predictions that the ability to identify core relational themes would be associated with effective emotion regulation and psychological well-being, supporting the Cascading Model of EI (Joseph and Newman 2010) and work on the protective effects of EU ability (Kashdan et al. 2015; Tugade et al. 2004). Notably, the CORE was related to certain criterion outcomes, even with demographic factors and the STEU-B in the model, providing some evidence in support of the CORE’s incremental validity. 

## 14. General Discussion

Understanding the causes and consequences of emotions, the differences between emotions, and the rich granularity inherent in emotion language is a valuable human ability (Castro et al. 2016; Mayer et al. 2016). We developed and presented validity evidence for a new test of EU ability—the CORE—which taps knowledge of primary meanings underlying a variety of emotions (i.e., core relational themes; Lazarus 1991). In Study 1, we developed the CORE items using the emotion literature to identify emotions with empirically supported themes (see Table 1), an expert panel, and a confusion matrix. In Study 2, the CORE showed high reliability (internal consistency) and a unidimensional factor structure. We also found evidence that the CORE converged with existing EU ability tests (i.e., the MSCEIT and STEU), and to an extent diverged from verbal intelligence and demographic variables, supporting its initial construct validity (AERA et al. 2014; Cronbach and Meehl 1955; Smith 2005). Further, we found a moderate to large negative link between the CORE and relational conflict, suggesting that people who better understand key semantic themes underlying emotions may experience less conflict, perhaps because they better understand why they and others feel the way they do (e.g., Sbarra and Coan 2018). This effect held with demographics and the MSCEIT or STEU in the model, indicating that the CORE may offer incremental validity, which few EU ability tests show. The CORE was found to be economical as well, taking between five to seven minutes to complete.

In Study 3, the CORE was positively related to adaptive coping, job satisfaction, meaning and purpose, and a mindset that emotions are malleable. The CORE also was negatively related to maladaptive coping and compassion fatigue, though it was unrelated to emotional exhaustion. Emotional exhaustion perhaps was driven more by factors outside of educators’ control during the pandemic, such as an increased workload and decreased boundaries between work and home (Steiner and Woo 2021). Prior studies suggest that structural demands and personal resources play a role in educator burnout and well-being (Granziera et al. 2021). Future studies could examine whether the CORE is associated with burnout when there is not a pandemic. Additionally, the CORE and STEU-B were positively related to emotional labor, counter to prediction. Those who better understand emotions may be more likely to identify aspects of work as emotional labor, and approach that labor, as they have skills to navigate it. Supporting this idea, adaptive coping—which is largely characterized by strategies to engage with emotional challenges—was positively related to emotional labor, and maladaptive coping—which is largely characterized by not processing emotional challenges—was negatively related to emotional labor (see Table 4; Carver 1997; Webb et al. 2012). Finally, the CORE remained associated with a set of outcomes in predicted directions, accounting for variance from demographic covariates and the STEU-B, offering further support of the CORE’s incremental validity. 

### 14.1. Theoretical Contributions 

#### 14.1.1. Core Relational Themes for 24 Emotions and Support for Semantic Space Theory 

Recent research supports the existence of 20 or more human emotions (e.g., Keltner et al. 2023). Yet, researchers have not examined whether people can reliably distinguish between core relational themes for this many emotions. In Study 1, respondents were given 24 different emotions to match to specific relational themes. Participants performed consistently above chance in matching the 24 emotions to the target theme. This included making distinctions within valence (e.g., pride from hope or anger from anxiety), and even among emotions from the same emotion family (e.g., shame from guilt or gratitude from love). Not only did people reliably identify the best answer, but their other responses were not picked at random. Participants’ non-target answers also were selected above chance on most items. Upon inspection, these answers appear to reflect the degrees of semantic overlap found in recent studies on emotion concepts (e.g., jealousy was a common answer for envy items). In this way, the results support Semantic Space Theory (Cowen and Keltner 2021; Keltner et al. 2023) and notions about emotion families (e.g., Sauter 2017), which propose that emotions are structured in a complex semantic network. Emotions similar in meaning are closer together in the network—without fully overlapping—and those different in meaning are farther apart. Importantly, this network structure of emotion appears to be more organized by substantive links between specific emotions than by shared valence and arousal levels (Cowen et al. 2019; Toivonen et al. 2012; cf. Jackson et al. 2019). 

#### 14.1.2. Support for The Cascading Model of EI and Emotion Granularity Theories

The Cascading Model of EI (Joseph and Newman 2010) and recent theorizing by emotional granularity researchers (Kashdan et al. 2015; Tugade et al. 2004) propose that the ability to differentiate between positive and negative emotion experiences should facilitate more targeted and thus successful emotion regulation. Further, the Cascading Model and emotion granularity theories hold that EU should be associated with higher performance and greater well-being to the extent that it enables more effective emotion regulation (Joseph and Newman 2010; Kashdan et al. 2015; Tugade et al. 2004). We found some support for these ideas. Individuals who more accurately identified relational themes on the CORE, reported engaging in coping strategies thought to support emotional health more often (e.g., acceptance and reappraisal), and they reported engaging in coping strategies considered deleterious less often (e.g., denial and substance use). CORE performance also was associated with a range of social-emotional (e.g., less conflict with friends, family, and romantic partners) and well-being outcomes (e.g., more meaning and purpose and lower compassion fatigue). Future research should test whether emotion regulation mediates the link between EU ability and key outcomes employing the CORE.

#### 14.1.3. The Generalizability of EU as an Ability and Its Predictive Value 

Most tests of EU ability were developed and validated with White, college-attending or college-graduate populations (see Table 2). This could limit the generalizability of the evidence supporting EU test validity (AERA et al. 2014). The CORE results suggest that EU ability can be reliably measured in demographically diverse U.S. adults, and that it is associated with healthier coping and social–emotional functioning across groups. These results help to generalize findings on EU ability, at least regarding the skill of identifying core relational themes underlying emotions. This is important as scholars propose that some features of emotion knowledge are universal; however, many tools to test these ideas have been validated only with select subpopulations, making it hard to substantiate such claims. Our research adds to the accumulation of data on central features of emotion concepts, including relational themes, suggesting that certain aspects of emotion knowledge may be shared by a wide variety of people (Jackson et al. 2019; Keltner et al. 2023). That said, more EU research with diverse participants is needed to confirm this is the case. 

### 14.2. Methodological Contributions 

#### 14.2.1. Increasing Measurement Precision in Assessing EU Ability 

Numerous dimensions of EU ability have been proposed, while only a few have been measured (Castro et al. 2016; Mayer et al. 2016). Among those that have been measured, other than the STEU, most EU tests combine scores across a few facets of EU (or offer scores for specific facets but do not validate tests for this purpose; see Table 2). Although providing general EU ability scores is useful for offering initial evidence of construct validity and test-criterion relationships, it limits measurement precision (Maul 2012). Testing theories of EU ability requires measurement approaches that permit examinations of EU’s component parts and their interrelations (Castro et al. 2016; Mayer et al. 2016). Also, measures that differentiate between specific facets of EU ability will help to unpack which EU skills are linked to other emotion abilities and outcomes. We developed and validated a new performance measure of EU ability that assesses *one* dimension of EU in depth. We hope that the CORE will help to isolate the associations unique to knowledge about relational themes and their value in predicting criterion-related outcomes. This level of construct representation may support next stage theory-testing in EU ability research. 

#### 14.2.2. Emotion Knowledge Can Be Measured Directly

Current EU ability tests rely primarily on context-based vignettes to tap EU (see Table 2). Such tests provide useful information about one’s knowledge relevant to a specific situation or domain (Hoemann et al. 2021a; Libbrecht and Lievens 2012). Yet, these tests measure emotion knowledge indirectly by asking people to infer how others might feel or react in certain situations. They also assume that people will interpret the situations similarly, and so if one understands emotions, they can report how others would feel. Given the wide variability in social norms and cultural standards influencing how people appraise the same situation or emotional stimulus (e.g., Cordaro et al. 2016a; Keltner et al. 2023; Moors 2020; van Rijn and Larrouy-Maestri 2023), using contextualized methods exclusively may limit knowledge in the field, and partially confound EU test performance with knowledge of sociocultural rules. The CORE was developed based on core relational themes that were identified across the literature and thought to represent shared meanings of emotions that are largely context-independent (see Table 1). These themes reflect how people make sense of emotional events and, we contend, are not as reliant on specific features of socially or culturally bound settings (noting cross-cultural studies on the CORE are needed). This approach affords the chance to study emotion knowledge directly. 

#### 14.2.3. The Value of Capturing Complexity with Progressive Scoring Approaches 

Recent massive-scale efforts supported by machine learning provide accumulating evidence that many features of emotions vary along multiple continuous dimensions, including emotion concepts (Cowen and Keltner 2017, 2021; Keltner et al. 2023). These findings diverge from notions that emotions are only “basic” (e.g., Ekman 1992), “discrete” (e.g., Roseman 2013), or “cultural constructions” of primal arousal and valence categories (Barrett 2017). They indicate that emotions have unique features which distinguish them, but they also share overlap, suggesting there are emotion families that are connected by *degrees of semantic relatedness* (Keltner and Cowen 2021; Sauter 2017). We developed and validated the CORE using a progressive scoring approach (e.g., Castro et al. 2015) to reflect this graded, meaning-based network structure of emotion concepts. To our knowledge, the CORE is the first EU ability test for adults in English that assesses degrees of correctness with answers rooted in theory and prior work. The CORE shows evidence of incremental validity over the most widely used measures of EU ability (MSCEIT and STEU), supporting the value of this method. In the development of new EU tests, this approach may help to better capture the complexity of emotional expertise (Hoemann et al. 2021a).

### 14.3. Research Limitations and Future Directions

The present research has limitations. In Studies 2 and 3, most participants performed above the mid-point on the CORE, suggesting the test may not capture the full range of ability. EI ability tests have faced challenges establishing defensible correctness criteria for test items that are easy, moderate, and difficult to answer (Fiori et al. 2014; Maul 2012; Miners et al. 2018). Ways to make the test more difficult may add construct-irrelevant variance (see AERA et al. 2014). We afford half credit for responses that are not the target response but are theoretically and empirically close to the target response, rather than oversimplifying emotion knowledge into dichotomies of correctness. We also used simple language, did not include complex social scenarios, and only measured one facet of EU ability versus measuring multiple facets. These steps may have reduced test difficulty, but perhaps did so (in part) by removing construct-irrelevant factors that influence test performance. We found evidence of test-criterion relationships between the CORE and multiple outcomes, accounting for other explanatory variables and measures of EU ability, so the test appears to capture variance that is psychologically meaningful. More research is needed on the semantic features of emotions to identify ways to validly capture EU ability among people with low, medium, and high emotional skill (Hoemann et al. 2021a). 

We took steps to minimize the role of demographic factors in test performance, including recruiting diverse samples for test construction and construct validation, and adding demographics to our analytic models (AERA et al. 2014). Associations between age, gender, and education with the CORE are akin to those found in other EU and EI ability studies. However, though race was unrelated to the CORE in Study 2, there was an inverse correlation between POC identity and the CORE in Study 3. To probe this result, we ran additional analyses (see Supplemental Materials). Part of this association came from third variables shared by race and CORE performance (e.g., education, extra work hours). To test whether the CORE was uniquely related to race in Study 3, we ran a multiple regression including these third variables and the STEU-B in the model. Then, POC identity only showed a small link to the CORE (*β* = −0.12, *p* < .01). As such, the association appears not to be unique to the CORE. To some extent, EU ability tests may reflect systemic inequities in education (AERA et al. 2014; Mahoney et al. 2021), and perhaps POC underrepresentation in psychological science (Buchanan et al. 2021; Roberts et al. 2020). A review of general EI ability tests indicates that this could be the case (see Joseph and Newman 2010). Important next steps include testing the CORE’s measurement invariance across race, other demographic and cultural groups, and intersectional identities, along with convening a fairness panel with relevant expertise and backgrounds to evaluate the CORE and recommend ways to make it more equitable (AERA et al. 2014). More broadly, it is important for researchers to examine how structural and social marginalization may influence EI abilities.

Additionally, we validated the CORE only in the U.S. with English-speaking samples. Future research could translate the tool and test its psychometric properties internationally to permit cross-cultural work on emotion concepts (e.g., Keltner et al. 2023). Research is needed that tests the universality of core relational themes, and the role they play in EU abilities in different cultural contexts (e.g., Castro et al. 2016). This work could be paired with studies of demographically diverse participants who work in various settings in the U.S. and abroad to test the generalizability of our findings (AERA et al. 2014). It will be important to determine the link between CORE performance and personality as well, and whether the CORE is related to outcomes beyond personality measures. Likewise, it will be helpful to test whether the CORE is associated with key outcomes independently of other mental abilities and intelligences given the overlap in these constructs. We also used self-reported relational conflict, coping, and well-being outcomes to gather evidence of the CORE’s test-criterion relationships. It would be useful to determine whether the CORE is related to second-person (e.g., job performance ratings) and third-person outcomes (e.g., cooperative behavior or physiological markers of stress) that tap social–emotional functioning, and more theoretically distal outcomes, along with measures of coping that reflect cultural differences in emotion regulation. Finally, all studies were cross-sectional in design, so to formally test the Cascading Model of EI with the CORE, longitudinal studies are needed that temporally separate EU ability, coping, and well-being. 

### 14.4. Implications for Research and Practice 

The ability model of EI was published over 30 years ago (Salovey and Mayer 1990). For years, the only performance measure of EU ability was the MSCEIT-Understanding subtest (Mayer et al. 2002), followed by the STEU (MacCann and Roberts 2008). Although, more recently, the GECO, GEMOK, and NEAT were developed, most of what is known about EU is still from the MSCEIT and STEU. When there are few adopted measures of a phenomena, it limits progress. Distinctions between construct and measurement variance are hard to make, and findings that may reflect how EU is measured may be mistaken for properties of the construct, or vice versa. This is particularly the case when only certain facets of a construct are assessed but are used to represent the entire phenomena, or when multiple facets are assessed but are averaged across, reducing measurement precision. We hope that by adding a new test to the field which measures a single facet of EU ability in depth, with evidence of reliability and validity, we help to improve the study of EU ability by representing the complexity of the phenomenon with increased precision. 

To increase accessibility, the CORE is available free of charge to researchers. This may help to stimulate further research on EU ability. Although the CORE was related to healthy coping patterns and well-being outcomes in working professionals, more studies are needed to determine whether the CORE can validly operate as a formative assessment in the world, in addition to serving as a summative research tool (see AERA et al. 2014). If such evidence is found, organizations interested in supporting the development of EU might use the CORE. Either way, we hope that the development of the CORE adds momentum to efforts to better understand EU ability inside and outside of the laboratory. 

## 15. Conclusions

Across three studies, with demographically diverse participants, we developed and provided validity evidence on the CORE. The CORE is a new EU ability measure that tests whether people can identify core relational themes (primary meanings) of 19 positive and negative emotions. The CORE employs progressive (degrees of correctness) scoring that is rooted in theory and prior research, aligning the test with developments in understanding the complex, interrelated structure of emotion concepts. Performance on the CORE was associated with more adaptive and less maladaptive coping, less relationship conflict and lower compassion fatigue, a greater sense of meaning and purpose, and a mindset that people can change their emotions. The CORE also captured unique variance in EU ability not measured by current EU tests, and it was related to theoretically relevant outcomes beyond variance accounted for by other tests. The CORE advances the study of EU ability by expanding the repertoire of reliable and valid performance tests in the field.

## Figures and Tables

**Table 1:** Core Relational Themes for 24 Emotions: Basis of The CORE Test Item Generation and Correctness Criteria.

Emotion	Core Relational Themes	Primary Citations
Amusement	Benign incongruity in thinking, speech, or action; Playful social rule violation; Absurdity or seeming nonsensical	Campos et al. (2013); Cordaro et al. (2016a); Fredrickson (2013)
Awe	Experiencing something greater than oneself in size, beauty, or meaning; Being in the presence of power	Gordon et al. (2017); Keltner (2023); Shiota et al. (2007, 2014)
Compassion	Witnessing suffering; Wanting to help or support others in need	Cowen and Keltner (2017); Goetz et al. (2010); Lazarus (1991)
Contentment	Sense of completeness, acceptance, or fulfillment	Campos et al. (2013); Cordaro et al. (2016b, 2021); Fredrickson (2013)
Gratitude	Receipt of specific benefits, acts of kindness, or generosity; Experiencing favorable life conditions (in general)	Campos et al. (2013); Emmons et al. (2019); Emmons and McCullough (2004); Fredrickson (2013)
Hope	Possible goal attainment (something might go well or could go well); Pathway to solving a problem or potential problem alleviation	Fredrickson (2013); Smith and Lazarus (1990, 1993); Snyder (1995, 2002)
Inspiration	Witnessing extraordinary moral virtue; Seeing resilience through hardship	Haidt (2000, 2003); Shiota et al. (2014); Thrash and Elliot (2003, 2004)
Interest	Novelty and attention-worthiness	Campos et al. (2013); Fredrickson (2013); Silvia (2005)
Joy	Free and safe to engage in play or have fun; Favorable news or outcome(s)	Campos et al. (2013); Cowen and Keltner (2017); Fredrickson (2013); Lazarus (1991) (see “happiness”)
Love	Full acceptance by another person; Reliable support from another person; Sharing precious moments, attention, and/or positive emotions with another person	Campos et al. (2013); Cowen and Keltner (2017) (see “adoration”); Fredrickson (2013); Lazarus (1991); Shaver et al. (1996)
Pride	Earned achievement from effortful action (authentic pride); Inflated sense of self-worth compared with others regardless of behavior (hubristic pride)	Campos et al. (2013); Cowen and Keltner (2017) (see “triumph”); Fredrickson (2013); Lazarus (1991); Tracy and Robins (2007, 2014)
Relief	A negative or undesired event goes away, does not happen, or is not as bad as expected	Cowen et al. (2019); Cowen and Keltner (2017); Lazarus (1991); Roseman (2013)
Anger	Goals are blocked or intentional action thwarted in some way; Experienced harm, offense, injustice, or unfairness—or witnessing it happen to others	Cowen and Keltner (2017); Fischer and Roseman (2007); Lazarus (1991); Roseman (2013) (see “anger” and “frustration”); Rozin et al. (1999); Smith and Lazarus (1990, 1993)
Anxiety	Experience of uncertainty; Something might go wrong or could be wrong	Cowen and Keltner (2017); Harmon-Jones et al. (2016); Lazarus (1991); Smith and Lazarus (1990, 1993)
Boredom	Experiencing monotony or repetitiveness; Irrelevance to the self or lack of meaningfulness	Cowen and Keltner (2017); Fahlman et al. (2013); Goldberg et al. (2011); van Tilburg and Igou (2017)
Disgust	Perceiving a stimulus or person as physically toxic or gross; Viewing a person, group, or idea as socially or morally toxic or gross (objectionable)	Fischer and Roseman (2007) (see “contempt”); Lazarus (1991); Roseman (2013) (see “disgust” and “contempt”); Rozin and Fallon (1987); Rozin et al. (1999, 2008)
Embarrassment	Committing minor infractions of social rules (faux pas); Awkwardness; The self becoming exposed publicly in a way that feels vulnerable or uncomfortable	Cowen et al. (2019); Cowen and Keltner (2017) (see “awkwardness”); Tangney (1999); Tangney et al. (1996, 2007)
Envy	Desiring or coveting what someone else has (that is perceived as valuable)	Lazarus (1991); Parrott and Smith (1993); Smith et al. (1988); Smith and Kim (2007)
Fear	Perceiving a clear and present danger or threat in one’s vicinity	Cowen and Keltner (2017); Harmon-Jones et al. (2016); Lazarus (1991); Roseman (2013)
Guilt	Transgressing valued societal norms or moral standards; Loss of social standing or reputation	Lazarus (1991); Niedenthal et al. (1994); Roseman (2013); Tangney et al. (1996); Tangney and Fischer (1995); Tracy et al. (2007)
Jealousy	Worrying that someone is going to take, or has taken away, something of value from you (especially a close social partner’s attention, time, and affection, romantic, platonic, or otherwise)	Lazarus (1991); Parrott and Smith (1993); Smith et al. (1988); Smith and Kim (2007)
Sadness	Missing or permanently losing something or someone of value; A desired outcome does not materialize	Cowen and Keltner (2017); Lazarus (1991); Roseman (2013); Smith and Lazarus (1990, 1993)
Shame	Violating one’s own internal norms or moral standards; Loss of perceived self-worth or self-regard	Lazarus (1991); Niedenthal et al. (1994); Roseman (2013); Tangney et al. (1996); Tangney and Fischer (1995); Tracy et al. (2007)
Surprise	Unexpectedness	Cowen and Keltner (2017); Ekman and Cordaro (2011); Noordewier and Breugelmans (2013); Roseman (2013)

Note. Relational themes are based on how a person appraises a given event or stimulus, rather than necessarily reflecting features of the event or stimulus itself.

**Table 2:** Comparison of Situation-Judgment and Performance-Based Tests of Emotion Understanding Ability (in English-Speaking Adults).

EU Ability Measure	CORE Test	MSCEIT-Understanding	STEU	GECo-Understanding	GEMOK-Blends and Features	NEAT-Perception and Understanding	LEAS
Primary Citation	N/A	Mayer et al. (2003)	MacCann and Roberts (2008)	Schlegel and Mortillaro (2019)	Schlegel and Scherer (2018)	Krishnakumar et al. (2016)	Lane et al. (1990)
Google Scholar Citation Count (as of 26 August 2023)	N/A	3073	715	94	38	64	1281
Construct Measured	Core relational themes of emotion	Emotion blends/ changes over time/intensity	Emotion appraisals (in general, work, personal contexts)	Emotion appraisals (e.g., novelty, power, valence)	Blends: Emotion appraisals and 4 other components (feeling, action tendencies, expression, and physiology) Features: Semantic knowledge about feature-emotion relationships	Emotion appraisals, emotion blends/transitions	Emotional awareness, emotional complexity, emotional development
Cost/ Accessibility	Free, available in supplemental materials	Available on MHS website; Researcher discount—$9 per subject (scored data set reports), Full report—$48 to $67 per subject; Certification required for non-researchers ^a^ (as of 18 April 2023)	Free, available in supplemental materials	Free for research purposes only (professional, commercial, or personal use is prohibited); Available upon request with signed research agreement on the University of Geneva website	Free, available in supplemental materials	N/A	Available on eLEAS website; Online administration and scoring: Researcher—$10 per subject, Clinician—$50 per subject, Student—$5 per subject (Paper/Manual scoring available) (as of 18 April 2023)
Mode of Assessment	Sentence completion task	Situation judgment task/vignettes	Situation judgment task/vignettes	Situation judgment task/vignettes	Blends: Situation judgment task/vignettes; Features: Semantic matching task	Situation judgement task/vignettes	Situation judgement task/vignette (open-ended responses)
Typical or Maximal Performance	Maximal	Maximal	Maximal	Maximal	Maximal	Maximal	Typical
Number of Items	38	32 (Blends: 12, Changes: 20)	42	20	120 (Blends: 20, Features: 100)	80 ratings: (Perception: 10, Understanding: 10) × 4 ratings each	20
Completion Time	5–7 min	30–45 min (full test battery)	N/A	50 min (full test battery)	N/A	N/A	30 min
Number of Emotions Measured	19 (10 positive and 9 negative): Amusement, awe, compassion, contentment, gratitude, hope, inspiration, joy, love, pride; anger, anxiety, boredom, disgust, embarrassment, envy, jealousy, sadness, shame	18 (8 positive and 10 negative): Admiration, contentment, gratitude, happiness, love, nostalgia, optimism, relief; anger, anxiety, confusion, disgust, guilt, hate, helplessness, jealousy, sadness, shame	14 (6 positive and 8 negative): Gratitude, hope, joy, pride, relief, surprise; angry, contempt, dislike, distressed, frustrated, regret, sad, scared	14 (4 positive and 10 negative): Happiness, interest, pride, relief; anger, anxiety, boredom, contempt, disgust, fear, guilt, irritation, sadness, shame	Blends: 15 (6 positive and 9 negative): Happiness, interest, joy, pleasure, pride, surprise; anger, anxiety, disgust, fear, guilt, irritation, jealousy, sadness, shame Features: 12 (5 positive and 7 negative): Interest, joy, pleasure, pride, surprise; anger, contempt, disgust, fear, guilt, sadness, shame	N/A	Self-generated
Correctness Criteria (theory, prior research, expert/population censensus)	Based on theory and prior research across multiple emotion science literatures (see Table 1)	Expert or population (*N* = 5000) consensus	Based on theory: Roseman’s (2001) Appraisal Theory	Based on theory: Component Process Model of emotion (Scherer 1984; Scherer et al. 2001)	Based on theory: Component Process Model of emotion (Scherer 1984; Scherer et al. 2001), and data from the GRID study (Fontaine et al. 2013) Features: Closeness to mean ratings from the GRID	Expert (MBA student) average ratings (*N* = 30)	Based on Levels of Emotional Awareness Theory (Lane and Schwartz 1987)
Scoring	Progressive scoring (no credit, half credit, full credit), based on theory and prior findings, cross-validated with a confusion matrix	Weighted scoring based on expert or population consensus scores	Binary scoring based on theory	Binary scoring based on theory	Blends: Binary scoring based on theory and GRID data Features: Profile correlations of participant ratings with GRID mean ratings	Weighted scoring based on expert consensus scores	Computer or hand scoring corresponding to Levels of Emotional Awareness Theory; Can sum individual self and other scores, or combine them
Reliability	Study 1: *a* = 0.90 Study 2: *a* = 0.94 Study 3: *a* = 0.96	Understanding total: General: Split-half = 0.80 Expert: Split-half = 0.77 Changes: General: *a* = 0.70 Expert: *a* = 0.68 Blends: General: *a* = 0.66 Expert: *a* = 0.62 Study 2: *a* = 0.84 (Present research)	Study 1: *a* = 0.71 Study 2: *a* = 0.43 Study 3: *a* = 0.83 (STEU-B) Study 2: *a* = 0.84 (Present research)	Study 2: Total: ω_t_ = 0.86 Understanding subtest: ω_t_ = 0.78 Study 3: Total: ω_t_ = 0.89 Understanding subtest: ω_t_ = 0.75	Study 1: Features: *a* = 0.89 Blends: *a* = 0.80 Study 2: Features: *a* = 0.88 Blends: *a* = 0.74 Study 3: Blends: *a* = 0.79 Study 4: Blends (brief): *a* = 0.67	Study 1: Perception: *a* = 0.87 Understanding: *a* = 0.81 Study 3: Perception: *a* = 0.89 Understanding: *a* = 0.77 Total: *a* = 0.92 Study 4: Perception: *a* = 0.89 Understanding: *a* = 0.75 Total: *a* = 0.92 Study 5: Perception: *a* = 0.92 Understanding: *a* = 0.69 Total: *a* = 0.93	*a* = 0.81 (Lane et al. 1990) *a* = 0.80–0.88 ^b^ (computer version: *a* = 0.84, *a* = 9.88) (Lane and Smith 2021)
Primary Sample (equal allocation, representative, purposive, other sampling method) ^c^	Study 1: Disproportionate stratified sample (equal allocation; see Table S2) Study 2: Quasi-representative sample (see Table S8) Study 3: POC participants oversampled (purposive sample; see Table S11)	*N* = 5000: 52% female, 37.3% male, 10.7% unreported; 58.6% White, 26.4% Asian, 5.4% Black, 4.9% Hispanic, 4.6% other; 58% some college, 14.9% college graduate, 5.5% master’s degree or higher; 37% age 20–29, 35% age 17–19, 12.7% unreported, 6.1% 30–39, 5.5% 40–49, 3.7% 50+ (Mayer et al. 2002) *N* = 2112: 58.6% female; *M* age = 26.25; 39.2% some college, 33.7% college graduate, 16.1% holding master’s level or higher; 34.0% Asian, 3.4% Black, 2.0% Hispanic, 57.9% White, and 2.3% other or mixed ethnicity; Majority U.S., but other countries sampled (Mayer et al. 2003)	Study 1: *N* = 207: 67.6% female; *M* age = 21.2; 53.1% Australian/Anglo-Celtic; 100% undergraduate students Study 2: *N* = 149: 71.8% female; *M* age = 35.33; 73.8% Australian/Anglo-Celtic; 68% postsecondary school	Item generation: 38, German- and French-speaking managers, HR, team leaders Item pre-test: 10 emotion researchers, 40 English speakers Study 1: *N* = 149: French speaking sample Study 2: *N* = 187: *M* age = 22.3; 55% female; 63% Asian, 33% Caucasian; 100% undergraduate/graduate students/staff members Study 3: *N* = 211: *M* age = 36.5; 53% female; 70% Caucasian; 56.4% college degree, 12.4% postgraduate degree Study 4: *N* = 206: Only 12% of responses administered in English Study 5: *N* = 113: German-speaking sample	Study 1: *N* = 443: *M* age = 45.4; 52% female; 65% Caucasian Study 2: *N* = 180: *M* age = 35.7; 50% female; 76% Caucasian Study 3: *N* = 87: *M* age = 33.5; 53% female; 75% Caucasian; Study 4: *N* = 103: *M* age = 32.7, 55% female, 74% Caucasian	All undergraduate samples Study 1: *N* = 290: *M* age = 19.8; 53% female Study 2: *N* = 578: *M* age = 19.6; 53% female Study 3: *N* = 96: *M* age = 19.2; 54% female Study 4: *N* = 85: *M* age = 19.2; 52.3% female Study 5: *N* = 91: *M* age = 21.1; 53% female; worked 20+ hours a week	N = 40: Yale undergraduates; 50% female; majority late teens, early 20s (all less than 30 years old)
Incremental Validity (over other EU ability measures)	Study 2: Associated with relational conflict above and beyond the MSCEIT or STEU Study 3: Associated with maladaptive coping, compassion fatigue, meaning and purpose, and a malleable emotion mindset above and beyond the STEU-B	N/A	N/A	Mean of recognition, understanding, and management subtests associated with average grade and exam points over the MSCEIT Total (in a subsample of German-speaking adults)	N/A	N/A	N/A

Note. N/A = Information was not available or could not be found. CORE = Core Relational Themes of Emotion Test; MSCEIT-Understanding = Mayer–Salovey–Caruso Emotional Intelligence Test-Understanding subtest; STEU = Situational Test of Emotion Understanding; GECo-Understanding = Geneva Emotional Competence Test-Understanding subtest; GEMOK-Blends and Features = Geneva EMOtion Knowledge test − Blends and Features; NEAT-Perception and Understanding = North Dakota Emotional Abilities Test − Perception and Understanding subtests; LEAS = Levels of Emotional Awareness Scale. ^a^ $2000–$2495 a person (based on posted prices of 2/5 U.S based companies qualified to run MSCEIT certification training as of 18 April 2023). ^b^ Some reported studies included non-English speaking samples or used very brief versions of the scale (e.g., 5 of 20 items). Those studies were removed from this range. ^c^ Sample category labels (e.g., Asian, White) were listed as they appear in the original studies and were not determined by the authors.

**Table 3:** Study 2: Zero-Order Correlations Among Latent Variables from CFAs and Covariates.

			Latent Study Variables				
Variable	*M*	SD	CORE	MSCEIT	STEU	V-IQ	Relational Conflict
Covariates							
Age	41.18	14.25	0.30 ***	0.33 ***	0.37 ***	0.40 ***	−0.24 ***
Gender (M/F)	0.50	0.50	0.17 **	0.18 *	0.22 **	−0.03	−0.12
Race (White/POC)	0.34	0.47	−0.04	−0.24 **	0.04	−0.09	0.11
Education (<4-year/>4-year degree)	0.46	0.50	−0.25 ***	−0.20 *	−0.23 **	−0.02	0.18 **
Latent Study Variables							
CORE	0.76	0.20	—				
MSCEIT	0.48	0.16	0.82 ***	—			
STEU	0.50	0.17	0.85 ***	—	—		
V-IQ	0.59	0.24	0.66 ***	0.66 ***	0.67 ***	—	
Relational Conflict	2.89	1.20	−0.39 ***	−0.30 ***	−0.42 ***	−0.27 ***	—

Note. *ns* = 140–284. CORE = Core Relational Themes of Emotion (CORE) Test. MSCEIT = Mayer–Salovey–Caruso Emotional Intelligence Test-Understanding subtest; STEU = Situational Test of Emotion Understanding; V-IQ = Verbal Intelligence. Participants were randomized to receive either the MSCEIT or the STEU. For the CORE, MSCEIT, STEU, and outcomes, we entered CFA-derived factor scores into the correlations. The reference group for binary variables is the last group in all cases. The mean and standard deviation values in the table reflect variable manifest means (not factor scores) for interpretability. * *p* < .05 ** *p* < .01 *** *p* < .001.

**Table 4:** Study 3: Zero-Order Correlations Among Latent Study Variables from CFAs and Covariates.

			EU Ability Measures		Coping		Well-Being Measures					
Variable	*M*	SD	CORE	STEU-B	Adaptive Coping	Maladaptive Coping	Emotional Exhaustion	Job Satisfaction	Emotional Labor	Compassion Fatigue	Meaning and Purpose	Emotion Mindset
Covariates												
Age	39.00	8.34	0.34 ***	0.34 ***	0.02	−0.31 ***	0.05	0.06	0.17 ***	−0.11 **	0.10 *	0.18 ***
Gender (M/F)	0.70	0.46	0.34 ***	0.38 ***	0.07	−0.34 ***	0.13 **	0.03	0.17 ***	−0.22 ***	0.06	0.16 ***
Race (White/POC)	0.61	0.49	−0.64 ***	−0.71 ***	−0.16 ***	0.49 ***	−0.23 ***	0.01	−0.32 ***	0.19 ***	−0.19 ***	−0.28 ***
Education (<Master’s Degree/ >Master’s)	0.53	0.50	0.45 ***	0.45 ***	0.15 ***	−0.34 ***	0.19 ***	−0.03	0.27 ***	−0.04	0.10 **	0.21 ***
Income (1 < $20K to 12 > $150 K)	6.57	2.40	0.10 *	0.07	−0.01	−0.08	0.19 ***	−0.02	0.12 *	0.07	0.06	0.03
EU Ability Measures												
CORE	0.73	0.25	—									
STEU-B	0.44	0.24	0.86 ***	—								
Coping												
Adaptive Coping	3.28	0.62	0.21 ***	0.22 ***	—							
Maladaptive Coping	2.45	0.84	−0.61 ***	−0.61 ***	−0.17 ***	—						
Well-Being Measures												
Emotional	3.57	1.48	0.14 **	0.22 ***	−0.04	0.05	—					
Exhaustion												
Job Satisfaction	4.60	1.04	0.12 **	0.07	0.23 ***	−0.09	−0.47 ***	—				
Emotional Labor	4.00	0.75	0.39 ***	0.48 ***	0.12 *	−0.16 ***	0.26 ***	0.12 *	—			
Compassion Fatigue	2.97	1.18	−0.34 ***	−0.32 ***	−0.02	0.48 ***	0.40 ***	−0.18 ***	0.10 *	—		
Meaning and Purpose	4.09	0.76	0.33 ***	0.33 ***	0.36 ***	−0.23 ***	−0.15 **	0.49 ***	0.30 ***	−0.18 ***	—	
Emotion Mindset	3.57	0.78	0.44 ***	0.34 ***	0.31 ***	−0.40 ***	−0.21 ***	0.24 ***	0.16 ***	−0.31 ***	0.42 ***	—

Note. *ns* = 306–460. EU = emotion understanding. CORE = Core Relational Themes of Emotion Test. STEU-B = Situational Test of Emotion Understanding-Brief. Emotion Mindset = malleable versus fixed emotion mindset. For the CORE, STEU-B, and outcome variables, we entered CFA-derived factor scores into the correlations. The reference group for binary variables is the last group in all cases. The mean and standard deviation values in the table reflect variable manifest means (not factor scores) for interpretability. * *p* < .05 ** *p* < .01 *** *p* < .001.

**Table 5:** Study 3: Multiple Regression Analyses Testing Incremental Validity of the CORE Above Demographic Factors and the STEU-B with Outcomes Measuring Adaptive Functioning (Using Latent Factor Scores).

	DV: Adaptive Coping			DV: Job Satisfaction			DV: Meaning and Purpose			DV: Emotion Mindset		
Step	*β*	*t*	SE	*β*	*t*	SE	*β*	*t*	SE	*Β*	*t*	SE
*Step 1*												
(Constant)		−0.26	0.28		−1.56	0.34		−0.01	0.32		0.24	0.24
Age	−0.00	−0.03	0.01	0.09	1.34	0.01	0.00	−0.00	0.01	0.01	0.19	0.01
Gender	0.07	1.09	0.11	0.04	0.60	0.13	−0.04	−0.69	0.13	0.02	0.33	0.10
Race	0.07	0.92	0.14	0.11	1.38	0.17	0.08	1.03	0.16	−0.07	−0.91	0.12
Education	0.14 *	2.10	0.11	−0.12	−1.73	0.13	−0.01	−0.20	0.13	0.02	0.31	0.10
Income	−0.13 *	−2.24	0.02	−.03	−0.43	0.03	−0.00	−0.08	0.03	−0.07	−1.20	0.02
STEU-B	0.20 *	2.37	0.08	0.16	1.87	0.10	0.42 ***	5.20	0.09	0.27 ***	3.33	0.07
*R*^2^		0.08			0.03			0.12			0.12	
*Step 2*												
(Constant)		−0.24	0.28		−1.45	0.34		0.24	0.31		0.59	0.24
Age	0.00	−0.07	0.01	0.07	1.16	0.01	−0.02	−0.39	0.01	−0.02	−0.32	0.01
Gender	0.07	1.09	0.11	0.04	0.60	0.13	−0.04	−0.69	0.12	0.02	0.35	0.09
Race	0.08	0.97	0.14	0.14	1.70	0.17	0.14	1.76	0.16	0.00	0.05	0.12
Education	0.14 *	2.04	0.11	−0.13	−1.93	0.13	−0.04	−0.63	0.12	−0.02	−0.24	0.09
Income	−0.13 *	−2.21	0.02	−0.02	−0.33	0.03	0.01	0.13	0.02	−0.06	−0.97	0.02
STEU-B	0.17	1.45	0.11	0.01	0.12	0.14	0.13	1.12	0.13	−0.11	−1.04	0.10
CORE	0.04	0.34	0.11	0.20	1.73	0.13	0.41 ***	3.72	0.12	0.54 ***	4.97	0.09
*R*^2^/*R*^2^ change	0.08/0.00			0.04/0.01			0.16/0.04 ***			0.19/0.07 ***		
	*F* (7, 293) = 3.69 **			*F* (7, 293) = 1.67			*F* (7, 293) = 8.16 ***			*F* (7, 293) = 9.78 ***		

Note. STEU-B = Situational Test of Emotional Understanding-Brief; CORE = Core Relational Themes of Emotion Test. Emotion Mindset = malleable versus fixed emotion mindset. For the CORE, STEU-B, and outcome variables, we entered CFA-derived factor scores into the regression models. A separate regression model was conducted for each outcome given the intercorrelations between variables. Gender (male = 0, female = 1); race (White = 0, POC = 1); and education (less than four-year college degree = 0, 1 = four-year college degree or higher). The reference group for binary variables is the last group in all cases. * *p* < .05 ** *p* < .01 *** *p* < .001.

**Table 6:** Study 3: Multiple Regression Analyses Testing Incremental Validity of the CORE Above Demographic Factors and the STEU-B with Outcomes Measuring Maladaptive Functioning (Using Latent Factor Scores).

	DV: Maladaptive Coping			DV: Emotional Exhaustion			DV: Emotional Labor			DV: Compassion Fatigue		
Step	*β*	*t*	SE	*β*	*t*	SE	*β*	*t*	SE	*β*	*t*	SE
*Step 1*												
(Constant)		1.95	0.22		0.22	0.31		−0.31	0.27		0.07	0.31
Age	−0.04	−0.89	0.01	−0.13 *	−2.15	0.01	−0.02	−0.35	0.01	−0.04	−0.71	0.01
Gender	−0.20 ***	−4.18	0.09	0.05	0.86	0.12	0.00	0.04	0.11	−0.13 *	−2.24	0.12
Race	0.10	1.50	0.11	−0.05	−0.62	0.15	0.05	0.73	0.14	0.11	1.38	0.15
Education	−0.08	−1.56	0.09	0.07	1.08	0.12	0.07	1.17	0.11	0.10	1.63	0.12
Income	−0.02	−0.49	0.02	0.22 ***	3.74	0.02	0.05	0.86	0.02	0.08	1.32	0.02
STEU-B	−0.42 ***	−6.36	0.06	0.16	1.95	0.09	0.49 ***	6.44	0.08	−0.23 **	−2.86	0.09
*R*^2^		0.43			0.11			0.24			0.14	
*Step 2*												
(Constant)		1.71	0.21		0.19	0.31		−0.17	0.27		−0.16	0.30
Age	−0.02	−0.45	0.01	−0.13 *	−2.09	0.01	−0.03	−0.58	0.01	−0.02	−0.36	0.01
Gender	−0.20 ***	−4.32	0.08	0.05	0.85	0.12	0.00	0.04	0.10	−0.13 *	−2.29	0.12
Race	0.04	0.64	0.11	−0.06	−0.71	0.16	0.08	1.13	0.14	0.06	0.71	0.15
Education	−0.06	−1.09	0.09	0.07	1.13	0.12	0.06	0.93	0.11	0.13 *	2.03	0.12
Income	−0.03	−0.75	0.02	0.22 ***	3.70	0.02	0.05	0.98	0.02	0.07	1.16	0.02
STEU-B	−0.14	−1.55	0.09	0.20	1.75	0.13	0.33 **	3.11	0.11	0.03	0.31	0.12
CORE	−0.39 ***	−4.43	0.08	−0.06	−0.52	0.12	0.22 *	2.12	0.10	−0.37 ***	−3.38	0.11
*R*^2^/*R*^2^ change	0.47/0.04 ***			0.11/0.00			0.25/0.01 *			0.17/0.03 **		
	*F* (7, 293) = 36.66 ***			*F* (7, 293) = 4.97 ***			*F* (7, 293) = 13.87 ***			*F* (7, 293) = 8.65 ***		

Note. STEU-B = Situational Test of Emotional Understanding-Brief; CORE = Core Relational Themes of Emotion Test. For the CORE, STEU-B, and outcome variables, we entered CFA-derived factor scores into the regression models. A separate regression model was conducted for each outcome given the intercorrelations between the variables. Gender (male = 0, female = 1); race (White = 0, POC = 1); and education (less than four-year college degree = 0, 1 = four-year college degree or higher). The reference group for binary variables is the last group in all cases. * *p* < .05 ** *p* < .01 *** *p* < .001.

## Data Availability

Our study data may be available upon request for research purposes.

## Notes

1 We refer to the test as “the CORE” throughout the paper.

2 There is necessary overlap between emotion appraisals and relational themes. There are different levels of analysis of emotional meaning (Smith and Lazarus 1993). For some emotions, multiple levels of analysis do not exist, and so the appraisal is the same as the relational theme, such as “unexpectedness” for surprise (Roseman 2013). For many emotions, however, appraisals and relational themes can be distinguished.

3 We note that semantic relations among emotions are likely structured in networks determined by multidimensional gradients of meaning, and appear not to be categorical entities with rigid boundaries (Cowen and Keltner 2017, 2021; Keltner et al. 2023).

4 A list of other EU ability tests validated in languages other than English is in the Supplemental Materials (see Table S1).

5 The Levels of Emotional Awareness Scale (LEAS; Lane et al. 1990) also may tap emotion knowledge to some extent, but it is thought to measure emotional awareness more than emotion understanding from the ability EI view (see Lane and Smith 2021).

6 Researchers, professors, and doctoral student members of the International Society for Research on Emotion.

7 There also are benefits to context-specific measures, as they may tap meaningful, socioculturally-embedded knowledge, which by some accounts, may be an inherent part of emotion concepts (e.g., see Hoemann et al. 2021a).

8 The MSCEIT-Understanding subtest and NEAT use weighted scoring that is non-binary, but it is based on expert ratings.

9 In accordance with the APA (2021) Inclusive Langauge Guidelines, these terms are intended to represent the following racial/ethnic categories, but are referred to with shorter labels for economy: Asian/Asian American, Black/African American, Latinx/Hispanic, and White/European American. We also use the word “race” to convey race and ethnicity.

10 We use the term “hit rate” hereafter for *pi* to make the results easier to interpret.

11 We reverse-scored this measure, so that higher scores indicate greater degrees of test interest/engagement.

12 Removing an emotion as a basis for an item did not preclude it from inclusion in the response set.

13 As EU ability is part of emotional intelligence, and intelligences correlate, we expected the CORE to overlap with cognitive intelligence, especially with verbal ability, as EU is particularly language-based (Mayer et al. 2016). However, we did not expect performance on the CORE to be redundant with verbal intelligence (Joseph and Newman 2010; Mayer et al. 2008a, 2008b).

14 For the sake of thoroughness, we also ran convergent, discriminant, and test-criterion relationship analyses using mean values (see the Supplemental Materials). The results did not substantively change, nor did our study conclusions.

15 People or Persons of Color (POC) (APA 2021).

16 Participants were randomized to complete either the MSCEIT-Understanding subtest or the STEU.

17 Given the high CORE-MSCEIT and STEU correlations, we checked for multicollinearity. Collinearity diagnostics for a model with all demographics, the CORE, and the MSCEIT were: VIF = 3.34 and tolerance = 0.30. Results for the same model with the STEU were: VIF = 3.85 and tolerance = 0.26. VIFs above 5 to 10 and tolerances below 0.1 to 0.2 indicate multicollinearity (Kim 2019; Kock and Lynn 2012). This did not appear to be an issue.

18 The STEU was selected as it is an established EU ability measure, and it measures facets of emotion appraisals that may overlap to an extent with core relational themes (on some items; see Table 2). It thus offers a more rigorous test of incremental validity.

19 The coping and emotion regulation literatures suggest that the extent to which a strategy is “adaptive” or “maladaptive” may be person, situation, and culture-specific (e.g., Bonanno and Burton 2013; Matsumoto et al. 2008). Yet, overall, meta-analytic studies suggest certain strategies are more versus less related to beneficial outcomes, including well-being and job performance (e.g., Webb et al. 2012). We thus use the terms adaptive and maladaptive coping based on these findings, keeping this caveat in mind, and noting that this study sample is from a Western individualistic culture.

20 A higher percentage of U.S. teachers hold master’s degrees than the general population (NCES 2023; U.S. Census Bureau 2020b).

21 The STEU-B was administered separately (two months prior) from the other Study 3 variables, noting ability EI is stable (e.g., Mayer et al. 2003).

22 Religion was dropped from the adaptive coping factor, and the distraction and venting items were dropped from the maladaptive coping factor, as their standardized loadings on their respective factors were ≤0.20. The reliability coefficients reflect these changes.

23 As with Study 2, we also report the results of all analyses in Study 3 using mean values in the Supplemental Materials. These analyses show the same general pattern of results as the factor score-based analyses, and do not change the study conclusions.

24 Given the high correlation of the CORE with the STEU-B, we checked for multicollinearity. Collinearity diagnostics for a model with all demographics, the CORE, and STEU-B were: VIF = 2.23 and tolerance = 0.45. Multicollinearity did not seem to be present (Kim 2019).

## Supplementary Materials

The following supporting information can be downloaded at: https://www.mdpi.com/article/10.3390/jintelligence11100195/s1, Table S1: Study 1: Additional EU Ability Measures for Adults (not Developed and Validated in English); Table S2: Study 1: Participant Demographic Characteristics; Table S3: Study 1: Item-Level Raw Hit Rate (Item Difficulty) on the CORE Test (Confusion Matrix); Table S4: Study 1: Emotion-Level Raw Hit Rate (Item Difficulty) on the CORE Test (Confusion Matrix); Table S5: Study 1: Item-Level Chance-Adjusted Hit Rate (Item Difficulty) on the CORE Test (Confusion Matrix); Table S6: Study 1: Emotion-Level Chance-Adjusted Hit Rate (Item Difficulty) on the CORE Test (Confusion Matrix); Table S7: Full Item Set and Scoring Key for the Core Relational Themes of Emotion (CORE) Test; Figure S1: Screenshots of the Core Relational Themes of Emotion (CORE) Test Instructions; Table S8: Study 2: Participant Demographic Characteristics; Table S9: Study 2: Factor Loadings from a One-Factor Confirmatory Factor Analysis (CFA) of the CORE; Table S10: Study 2: Zero-Order Correlations Among Key Study Variables and Covariates (Mean Values); Table S11: Study 3: Participant Demographic Characteristics; Table S12: Study 3: Zero-Order Correlations Among Key Study Variables and Covariates (Mean Values); Table S13: Study 3: Multiple Regression Analyses Testing Incremental Validity of the CORE Above Demographics and the STEU-B with Outcomes Measuring Adaptive Functioning (Mean Values); Table S14: Study 3: Multiple Regression Testing Incremental Validity of the CORE Above Demographics and the STEU-B with Outcomes Measuring Maladaptive Functioning (Mean Values).

## References

[B1-jintelligence-11-00195] American Educational Research Association (AERA), American Psychological Association (APA), National Council on Measurement in Education (NCME) (2014). Standards for Educational and Psychological Testing.

[B2-jintelligence-11-00195] Allen Veleka D., Weissman Alexander, Hellwig Susan, MacCann Carolyn, Roberts Richard D. (2014). Development of the situational test of emotional understanding—brief (STEU-B) using item response theory. Personality and Individual Differences.

[B3-jintelligence-11-00195] American Psychological Association (APA) (2021). Inclusive Language Guidelines. https://www.apa.org/about/apa/equity-diversity-inclusion/language-guidelines.pdf.

[B4-jintelligence-11-00195] Aristotle (2009). The Nicomachean Ethics.

[B5-jintelligence-11-00195] Barrett Lisa F. (2017). How Emotions Are Made: The Secret Life of the Brain and What It Means for Your health, the Law, and Human Nature.

[B6-jintelligence-11-00195] Barrett Lisa F., Russell James A. (2014). The Psychological Construction of Emotion.

[B7-jintelligence-11-00195] Bollen Kenneth A. (2002). Latent variables in psychology and the social sciences. Annual Review of Psychology.

[B8-jintelligence-11-00195] Bonanno George A., Burton Charles L. (2013). Regulatory flexibility: An individual differences perspective on coping and emotion regulation. Perspectives on Psychological Science.

[B9-jintelligence-11-00195] Brackett Marc A., Warner Rebecca M., Bosco Jennifer S. (2005). Emotional intelligence and relationship quality among couples. Personal Relationships.

[B10-jintelligence-11-00195] Brackett Marc A., Rivers Susan E., Salovey Peter (2011). Emotional intelligence: Implications for personal, social, academic, and workplace success. Social and Personality Psychology Compass.

[B11-jintelligence-11-00195] Buchanan NiCole T., Perez Marisol, Prinstein Mitchell J., Thurston Idia B. (2021). Upending racism in psychological science: Strategies to change how science is conducted, reported, reviewed, and disseminated. American Psychologist.

[B12-jintelligence-11-00195] Buhrmester Duane, Furman Wyndol (2008). The Network of Relationships Inventory: Relationship Qualities Version.

[B13-jintelligence-11-00195] Campos Belinda, Shiota Michelle N., Keltner Dacher, Gonzaga Gian C., Goetz Jennifer L. (2013). What is shared, what is different? Core relational themes and expressive displays of eight positive emotions. Cognition and Emotion.

[B14-jintelligence-11-00195] Carver Charles S. (1997). You want to measure coping but your protocol’s too long: Consider the brief cope. International Journal of Behavioral Medicine.

[B15-jintelligence-11-00195] Carver Charles S., Vargas Sara, Friedman Howard S. (2011). Stress, coping, and health. The Oxford Handbook of Health Psychology.

[B16-jintelligence-11-00195] Castella Krista D., Goldin Philippe, Jazaieri Hooria, Ziv Michal, Dweck Carol S., Gross James J. (2013). Beliefs about emotion: Links to emotion regulation, well-being, and psychological distress. Basic and Applied Social Psychology.

[B17-jintelligence-11-00195] Castro Vanessa L., Halberstadt Amy G., Lozada Fantasy T., Craig Ashley B. (2015). Parents’ emotion-related beliefs, behaviours, and skills predict children’s recognition of emotion. Infant and Child Development.

[B18-jintelligence-11-00195] Castro Vanessa L., Cheng Yanhua, Halberstadt Amy G., Grühn Daniel (2016). EUReKA! A conceptual model of emotion understanding. Emotion Review.

[B19-jintelligence-11-00195] Cohen Jacob (1988). Statistical Power Analysis for the Behavioral Sciences.

[B20-jintelligence-11-00195] Cohen Jacob (1992). A power primer. Psychological Bulletin.

[B21-jintelligence-11-00195] Cor M. Ken, Haertel Edward, Krosnick Jon A., Malhotra Neil (2012). Improving ability measurement in surveys by following the principles of IRT: The Wordsum vocabulary test in the General Social Survey. Social Science Research.

[B22-jintelligence-11-00195] Cordaro Daniel T., Bradley Christina, Zhang Jia W., Zhu Franklyn, Han Rachel (2021). The development of the positive emotion assessment of contentment experience (peace) scale. Journal of Happiness Studies.

[B23-jintelligence-11-00195] Cordaro Daniel T., Keltner Dacher, Tshering Sumjay, Wangchuk Dorji, Flynn Lisa M. (2016a). The voice conveys emotion in ten globalized cultures and one remote village in Bhutan. Emotion.

[B24-jintelligence-11-00195] Cordaro Daniel T., Brackett Marc, Glass Lauren, Anderson Craig L. (2016b). Contentment: Perceived completeness across cultures and traditions. Review of General Psychology.

[B25-jintelligence-11-00195] Cowen Alan, Sauter Disa, Tracy Jessica L., Keltner Dacher (2019). Mapping the passions: Toward a high-dimensional taxonomy of emotional experience and expression. Psychological Science in the Public Interest.

[B26-jintelligence-11-00195] Cowen Alan S., Keltner Dacher (2017). Self-report captures 27 distinct categories of emotion bridged by continuous gradients. Proceedings of the National Academy of Sciences USA.

[B27-jintelligence-11-00195] Cowen Alan S., Keltner Dacher (2021). Semantic space theory: A computational approach to emotion. Trends in Cognitive Sciences.

[B28-jintelligence-11-00195] Cronbach Lee J., Meehl Paul E. (1955). Construct validity in psychological tests. Psychological Bulletin.

[B29-jintelligence-11-00195] Curran Paul G. (2016). Methods for the detection of carelessly invalid responses in survey data. Journal of Experimental Social Psychology.

[B30-jintelligence-11-00195] Daniel Johnnie, Daniel Johnnie (2012). Choosing the type of probability sampling. Sampling Essentials: Practical Guidelines for Making Sampling Choices.

[B31-jintelligence-11-00195] Diener Ed (2009). The Science of Well Being: The Collected Works of Ed Diener.

[B32-jintelligence-11-00195] Ekman Paul (1992). Are there basic emotions?. Psychological Review.

[B33-jintelligence-11-00195] Ekman Paul, Cordaro Daniel (2011). What is meant by calling emotions basic. Emotion Review.

[B34-jintelligence-11-00195] Emmons Robert A., McCullough Michael E. (2004). The Psychology of Gratitude.

[B35-jintelligence-11-00195] Emmons Robert A., Froh Jeffrey, Rose Rachel, Gallagher Matthew W., Lopez Shane J. (2019). Gratitude. Positive Psychological Assessment: A Handbook of Models and Measures.

[B36-jintelligence-11-00195] Fahlman Shelley A., Mercer-Lynn Kimberley B., Flora David B., Eastwood John D. (2013). Development and validation of the Multidimensional State Boredom Scale. Assessment.

[B37-jintelligence-11-00195] Fernández-Berrocal Pablo, Extremera Natalio (2006). Emotional intelligence: A theoretical and empirical review of its first 15 years of history. Psicothema.

[B38-jintelligence-11-00195] Fernández-Berrocal Pablo, Extremera Natalio (2016). Ability emotional intelligence, depression, and well-being. Emotion Review.

[B39-jintelligence-11-00195] Fey Carl F., Hu Tianyou, Delios Andrew (2023). The measurement and communication of effect sizes in management research. Management and Organization Review.

[B40-jintelligence-11-00195] Figley Charles R., Stamm Beth H. (1995). Compassion fatigue: Toward a new understanding of the costs of caring. Secondary Traumatic Stress: Self-Care Issues for Clinicians, Researchers, and Educators.

[B41-jintelligence-11-00195] Fiori Marina, Antonietti Jean-Philippe, Mikolajczak Moïra, Luminet Olivier, Hansenne Michel, Rossier Jérôme (2014). What is the ability emotional intelligence test (MSCEIT) good for? An evaluation using item response theory. PLoS ONE.

[B42-jintelligence-11-00195] Fischer Agneta H., Roseman Ira J. (2007). Beat them or ban them: The characteristics and social functions of anger and contempt. Journal of Personality and Social Psychology.

[B43-jintelligence-11-00195] Fontaine Johnny J. R., Scherer Klaus R., Soriano Cristina, Fontaine Johnny J. R., Scherer Klaus R., Soriano Cristina (2013). The why, the what, and the how of the GRID instrument. Components of Emotional Meaning: A Sourcebook.

[B44-jintelligence-11-00195] Fredrickson Barbara L., Devine Patricia, Plant Ashby (2013). Positive emotions broaden and build. Advances in Experimental Social Psychology.

[B45-jintelligence-11-00195] Goetz Jennifer L., Keltner Dacher, Simon-Thomas Emiliana (2010). Compassion: An evolutionary analysis and empirical review. Psychological Bulletin.

[B46-jintelligence-11-00195] Goldberg Yael K., Eastwood John D., LaGuardia Jennifer, Danckert James (2011). Boredom: An emotional experience distinct from apathy, anhedonia, or depression. Journal of Social and Clinical Psychology.

[B47-jintelligence-11-00195] Gordon Amie M., Stellar Jennifer E., Anderson Craig L., McNeil Galen D., Loew Daniel, Keltner Dacher (2017). The dark side of the sublime: Distinguishing a threat-based variant of awe. Journal of Personality and Social Psychology.

[B48-jintelligence-11-00195] Grandey Alicia A., Gabriel Allison S. (2015). Emotional labor at a crossroads: Where do we go from here?. Annual Review of Organizational Psychology.

[B49-jintelligence-11-00195] Granziera Helena, Collie Rebecca, Martin Andrew, Mansfield Caroline F. (2021). Understanding Teacher Wellbeing Through Job Demands-Resources Theory. Cultivating Teacher Resilience: International Approaches, Applications, and Impact.

[B50-jintelligence-11-00195] Haidt Jonathan (2000). The positive emotion of elevation. Prevention & Treatment.

[B51-jintelligence-11-00195] Haidt Jonathan, Keyes Corey L. M., Haidt Jonathan (2003). Elevation and the positive psychology of morality. Flourishing: Positive Psychology and the Life Well-Lived.

[B52-jintelligence-11-00195] Hall Judith A., Andrzejewski Susan A., Murphy Nora A., Mast Marianne S., Feinstein Brian A. (2008). Accuracy of judging others’ traits and states: Comparing mean levels across tests. Journal of Research in Personality.

[B53-jintelligence-11-00195] Harmon-Jones Cindy, Bastian Brock, Harmon-Jones Eddie (2016). The discrete emotions questionnaire: A new tool for measuring state self-reported emotions. PLoS ONE.

[B54-jintelligence-11-00195] Hoemann Katie, Nielson Catie, Yuen Ashley, Gurera J. W., Quigley Karen S., Barrett Lisa F. (2021a). Expertise in emotion: A scoping review and unifying framework for individual differences in the mental representation of emotional experience. Psychological Bulletin.

[B55-jintelligence-11-00195] Hoemann Katie, Khan Zulqarnain, Kamona Nada, Dy Jennifer, Barrett Lisa F., Quigley Karen S. (2021b). Investigating the relationship between emotional granularity and cardiorespiratory physiological activity in daily life. Psychophysiology.

[B56-jintelligence-11-00195] Hooper Daire, Coughlan Joseph, Mullen Michael R., Brown Ann (2008). Evaluating model fit: A synthesis of the structural equation modelling literature. Paper presented at the 7th European Conference on Research Methodology for Business and Management Studies.

[B57-jintelligence-11-00195] Hu Li-tze, Bentler Peter M. (1999). Cutoff criteria for fit indexes in covariance structure analysis: Conventional criteria versus new alternatives. Structural Equation Modeling: A Multidisciplinary Journal.

[B58-jintelligence-11-00195] Hu Tianqiang, Zhang Dajun, Wang Jinliang, Mistry Ritesh, Ran Guangming, Wang Xinqiang (2014). Relation between emotion regulation and mental health: A meta-analysis review. Psychological Reports: Measures & Statistics.

[B59-jintelligence-11-00195] Jackson Joshua C., Watts Joseph, Henry Teague R., List Johann-Mattis, Forkel Robert, Mucha Peter J., Greenhill Simon J., Gray Russell D., Lindquist Kristen A. (2019). Emotion semantics show both cultural variation and universal structure. Science.

[B60-jintelligence-11-00195] Joseph Dana L., Newman Daniel A. (2010). Emotional intelligence: An integrative meta-analysis and cascading model. Journal of Applied Psychology.

[B61-jintelligence-11-00195] Kalkbrenner Michael T. (2023). Alpha, Omega, and H internal consistency reliability estimates: Reviewing these options and when to use them. Counseling Outcome Research and Evaluation.

[B62-jintelligence-11-00195] Kashdan Todd B., Barrett Lisa F., McKnight Patrick E. (2015). Unpacking emotion differentiation: Transforming unpleasant experience by perceiving distinctions in negativity. Current Directions in Psychological Science.

[B63-jintelligence-11-00195] Keltner Dacher (2023). AWE: The New Science of Everyday Wonder and How It Can Transform Your Life.

[B64-jintelligence-11-00195] Keltner Dacher, Cowen Alan (2021). A taxonomy of positive emotions. Current Opinion in Behavioral Sciences.

[B65-jintelligence-11-00195] Keltner Dacher, Sauter Disa, Tracy Jessica L., Wetchler Everett, Cowen Alan S. (2022). How emotions, relationships, and culture constitute each other: Advances in social functionalist theory. Cognition and Emotion.

[B66-jintelligence-11-00195] Keltner Dacher, Brooks Jeffrey A., Cowen Alan (2023). Semantic space theory: Data-driven insights into basic emotions. Current Directions in Psychological Science.

[B67-jintelligence-11-00195] Kim Jong H. (2019). Multicollinearity and misleading statistical results. Korean Journal of Anesthesiology.

[B68-jintelligence-11-00195] Kock Ned, Lynn Gary S. (2012). Lateral collinearity and misleading results in variance-based SEM: An illustration and recommendations. Journal of the Association for Information Systems.

[B69-jintelligence-11-00195] Kotsou Ilios, Mikolajczak Moïra, Heeren Alexandre, Grégoire Jacques, Leys Christophe (2019). Improving emotional intelligence: A systematic review of existing work and future challenges. Emotion Review.

[B70-jintelligence-11-00195] Krishnakumar Sukumarakurup, Hopkins Kay, Szmerekovsky Joseph G., Robinson Michael D. (2016). Assessing workplace emotional intelligence: Development and validation of an ability-based measure. The Journal of Psychology: Interdisciplinary and Applied.

[B71-jintelligence-11-00195] Kung Franki Y. H., Kwok Navio, Brown Douglas J. (2018). Are attention check questions a threat to scale validity?. Applied Psychology.

[B72-jintelligence-11-00195] Lane Richard D., Schwartz Gary E. (1987). Levels of emotional awareness: A cognitive-developmental theory and its application to psychopathology. The American Journal of Psychiatry.

[B73-jintelligence-11-00195] Lane Richard D., Smith Ryan (2021). Levels of emotional awareness: Theory and measurement of a socio-emotional skill. Journal of Intelligence.

[B74-jintelligence-11-00195] Lane Richard D., Quinlan Donald M., Schwartz Gary E., Walker Pamela A., Zeitlin Sharon B. (1990). The Levels of Emotional Awareness Scale: A cognitive-developmental measure of emotion. Journal of Personality Assessment.

[B75-jintelligence-11-00195] LaPalme Matthew L., Barsade Sigal G., Brackett Marc A., Floman James L. (2023). The Meso-Expression Test (MET): A novel assessment of emotion perception. Journal of Intelligence.

[B76-jintelligence-11-00195] Laukka Petri, Elfenbein Hillary A., Thingujam Nutankumar S., Rockstuhl Thomas, Iraki Frederick K., Chui Wanda, Althoff Jean (2016). The expression and recognition of emotions in the voice across five nations: A lens model analysis based on acoustic features. Journal of Personality and Social Psychology.

[B77-jintelligence-11-00195] Lazarus Richard S. (1991). Emotion and Adaptation.

[B78-jintelligence-11-00195] Li Cheng-Hsien (2016). Confirmatory factor analysis with ordinal data: Comparing robust maximum likelihood and diagonally weighted least squares. Behavior Research Methods.

[B79-jintelligence-11-00195] Libbrecht Nele, Lievens Filip (2012). Validity evidence for the situational judgement test paradigm in emotional intelligence measurement. International Journal of Psychology.

[B80-jintelligence-11-00195] Lopes Paulo N., Brackett Marc A., Nezlek John B., Schütz Astrid, Sellin Ina, Salovey Peter (2004). Emotional intelligence and social interaction. Personality and Social Psychology Bulletin.

[B81-jintelligence-11-00195] Lopes Paulo N., Salovey Peter, Straus Rebecca (2003). Emotional intelligence, personality, and the perceived quality of social relationships. Personality and Individual Differences.

[B82-jintelligence-11-00195] Lyubomirsky Sonja (2008). The How of Happiness: A Scientific Approach to Getting the Life You Want.

[B83-jintelligence-11-00195] MacCann Carolyn, Roberts Richard D. (2008). New paradigms for assessing emotional intelligence: Theory and data. Emotion.

[B84-jintelligence-11-00195] Madigan Daniel J., Kim Lisa E. (2021). Towards an understanding of teacher attrition: A meta-analysis of burnout, job satisfaction, and teachers’ intentions to quit. Teaching and Teacher Education.

[B85-jintelligence-11-00195] Mahoney Joseph L., Weissberg Roger P., Greenberg Mark T., Dusenbury Linda, Jagers Robert J., Niemi Karen, Schlinger Melissa, Schlund Justina, Shriver Timothy P., VanAusdal Karen (2021). Systemic social and emotional learning: Promoting educational success for all preschool to high school students. American Psychologist.

[B86-jintelligence-11-00195] Maslach Christina, Jackson Susan E., Leiter Michael P. (1996). MBI: Maslach Burnout Inventory.

[B87-jintelligence-11-00195] Matsumoto David, Yoo Seung H., Nakagawa Sanae (2008). Culture, emotion regulation, and adjustment. Journal of Personality and Social Psychology.

[B88-jintelligence-11-00195] Maul Andrew (2012). The validity of the Mayer-Salovey-Caruso Emotional Intelligence Test (MSCEIT) as a measure of emotional intelligence. Emotion Review.

[B89-jintelligence-11-00195] Mayer John D., Caruso David R., Salovey Peter (1999). Emotional intelligence meets traditional standards for an intelligence. Intelligence.

[B90-jintelligence-11-00195] Mayer John D., Caruso David R., Salovey Peter (2016). The ability model of emotional intelligence: Principles and updates. Emotion Review.

[B91-jintelligence-11-00195] Mayer John D., Salovey Peter, Caruso David R. (2002). Mayer-Salovey-Caruso Emotional Intelligence Test (MSCEIT) User’s Manual.

[B92-jintelligence-11-00195] Mayer John D., Salovey Peter, Caruso David R. (2008a). Emotional intelligence: New ability or eclectic traits?. American Psychologist.

[B93-jintelligence-11-00195] Mayer John D., Salovey Peter, Caruso David R. (2012). The validity of the MSCEIT: Additional analyses and evidence. Emotion Review.

[B94-jintelligence-11-00195] Mayer John D., Salovey Peter, Caruso David R., Sitarenios Gill (2003). Measuring emotional intelligence with the MSCEIT V2.0. Emotion.

[B95-jintelligence-11-00195] Mayer John D., Roberts Richard D., Barsade Sigal G. (2008b). Human abilities: Emotional intelligence. Annual Review of Psychology.

[B96-jintelligence-11-00195] McNeish Daniel, Wolf Melissa G. (2020). Thinking twice about sum scores. Behavior Research Methods.

[B97-jintelligence-11-00195] Miners Christopher T. H., Côté Stéphane, Lievens Filip (2018). Assessing the validity of emotional intelligence measures. Emotion Review.

[B98-jintelligence-11-00195] Monroy Maria, Cowen Alan S., Keltner Dacher (2022). Intersectionality in emotion signaling and recognition: The influence of gender, ethnicity, and social class. Emotion.

[B99-jintelligence-11-00195] Moors Agnes (2014). Flavors of appraisal theories of emotion. Emotion Review.

[B100-jintelligence-11-00195] Moors Agnes, Zeigler-Hill Virgil, Shackelford Todd K. (2020). Appraisal theory of emotion. Encyclopedia of Personality and Individual Differences.

[B101-jintelligence-11-00195] National Center for Education Statistics (NCES) (2023). Characteristics of Public School Teachers.

[B102-jintelligence-11-00195] New Teacher Center (2017). Teaching, Empowering, Leading, and Learning (TELL) Survey. https://newteachercenter.org/approach-old/teaching-empowering-leading-and-learning-tell/.

[B103-jintelligence-11-00195] Newman Daniel A., Joseph Dana L., MacCann Carolyn (2010). Emotional intelligence and job performance: The importance of emotion regulation and emotional labor context. Industrial and Organizational Psychology.

[B104-jintelligence-11-00195] Niedenthal Paula M., Tangney June P., Gavanski Igor (1994). “If only I weren’t” versus “If only I hadn’t”: Distinguishing shame and guilt in counterfactual thinking. Journal of Personality and Social Psychology.

[B105-jintelligence-11-00195] Noordewier Marret K., Breugelmans Seger M. (2013). On the valence of surprise. Cognition & Emotion.

[B106-jintelligence-11-00195] Page Kathryn M., Vella-Brodrick Dianne A. (2009). The ‘what’, ‘why’ and ‘how’ of employee well-being: A new model. Social Indicators Research.

[B107-jintelligence-11-00195] Parrott W. Gerrod, Smith Richard H. (1993). Distinguishing the experiences of envy and jealousy. Journal of Personality and Social Psychology.

[B108-jintelligence-11-00195] Rdz-Navarro Karina (2019). Latent variables should remain as such: Evidence from a Monte Carlo study. The Journal of General Psychology.

[B109-jintelligence-11-00195] Roberts Steven O., Bareket-Shavit Carmelle, Dollins Forrest A., Goldie Peter D., Mortenson Elizabeth (2020). Racial inequality in psychological research: Trends of the past and recommendations for the future. Perspectives on Psychological Science.

[B110-jintelligence-11-00195] Roseman Ira J., Scherer Klaus R., Schorr Angela, Johnstone Tom (2001). A model of appraisal in the emotion system: Integrating theory, research, and applications. Appraisal Processes in Emotion.

[B111-jintelligence-11-00195] Roseman Ira J. (2013). Appraisal in the emotion system: Coherence in strategies for coping. Emotion Review.

[B112-jintelligence-11-00195] Rosenthal Robert, Rubin Donald B. (1989). Effect size estimation for one-sample multiple-choice-type data: Design, analysis, and meta-analysis. Psychological Bulletin.

[B113-jintelligence-11-00195] Rozin Paul, Fallon April E. (1987). A perspective on disgust. Psychological Review.

[B114-jintelligence-11-00195] Rozin Paul, Haidt Jonathan, McCauley Clark R., Lewis Michael, Haviland-Jones Jeannette M., Barrett Lisa F. (2008). Disgust. Handbook of Emotions.

[B115-jintelligence-11-00195] Rozin Paul, Lowery Laura, Imada Sumio, Haidt Jonathan (1999). The CAD triad hypothesis: A mapping between three moral emotions (contempt, anger, disgust) and three moral codes (community, autonomy, divinity). Journal of Personality and Social Psychology.

[B116-jintelligence-11-00195] Ryff Carol D., Singer Burton H. (2008). Know thyself and become what you are: A eudaimonic approach to psychological well-being. Journal of Happiness Studies.

[B117-jintelligence-11-00195] Salovey Peter, Mayer John D. (1990). Emotional Intelligence. Imagination, Cognition, and Personality.

[B118-jintelligence-11-00195] Salsman John M., Schalet Benjamin D., Park Crystal L., George Login, Steger Michael F., Hahn Elizabeth A., Snyder Mallory A., Cella David (2020). Assessing meaning & purpose in life: Development and validation of an item bank and short forms for the NIH PROMIS. Qualify of Life Research.

[B119-jintelligence-11-00195] Sánchez-Álvarez Nicolás, Extremera Natalio, Fernández-Berrocal Pablo (2016). The relation between emotional intelligence and subjective well-being: A meta-analytic investigation. The Journal of Positive Psychology.

[B120-jintelligence-11-00195] Sauter Disa A. (2017). The nonverbal communication of positive emotions: An emotion family approach. Emotion Review.

[B121-jintelligence-11-00195] Sbarra David A., Coan James A. (2018). Relationships and health: The critical role of affective science. Emotion Review.

[B122-jintelligence-11-00195] Scherer Klaus R., Scherer Klaus R., Ekman Paul (1984). On the nature and function of emotion: A component process approach. Approaches to Emotion.

[B123-jintelligence-11-00195] Scherer Klaus R. (2019). Studying appraisal-driven emotion processes: Taking stock and moving to the future. Cognition and Emotion.

[B124-jintelligence-11-00195] Scherer Klaus R., Schorr Angela, Johnstone Tom (2001). Appraisal Processes in Emotion: Theory, Methods, Research.

[B125-jintelligence-11-00195] Schlegel Katja, Scherer Klaus R. (2018). The nomological network of emotion knowledge and emotion understanding in adults: Evidence from two new performance-based tests. Cognition and Emotion.

[B126-jintelligence-11-00195] Schlegel Katja, Mortillaro Marcello (2019). The Geneva Emotional Competence Test (GECo): An ability measure of workplace emotional intelligence. Journal of Applied Psychology.

[B127-jintelligence-11-00195] Seligman Martin E. P. (2011). Flourish: A Visionary New Understanding of Happiness and Well-Being.

[B128-jintelligence-11-00195] Shaver Phillip, Schwartz Judith, Kirson Donald, O’Connor Cary (1987). Emotion knowledge: Further exploration of a prototype approach. Journal of Personality and Social Psychology.

[B129-jintelligence-11-00195] Shaver Phillip R., Morgan Hillary J., Wu Shelley (1996). Is love a “basic” emotion?. Personal Relationships.

[B130-jintelligence-11-00195] Shiota Michelle N., Keltner Dacher, Mossman Amanda (2007). The nature of awe: Elicitors, appraisals, and effects on self-concept. Cognition and Emotion.

[B131-jintelligence-11-00195] Shiota Michelle N., Thrash Todd M., Danvers Alexander F., Dombrowski John T., Tugade Michele M., Shiota Michelle N., Kirby Leslie D. (2014). Transcending the self: Awe, elevation, and inspiration. Handbook of Positive Emotions.

[B132-jintelligence-11-00195] Silvia Paul J. (2005). What is interesting? Exploring the appraisal structure of interest. Emotion.

[B133-jintelligence-11-00195] Simon-Thomas Emiliana R., Keltner Dacher J., Sauter Disa, Sinicropi-Yao Lara, Abramson Anna (2009). The voice conveys specific emotions: Evidence from vocal burst displays. Emotion.

[B134-jintelligence-11-00195] Smith Craig A., Lazarus Richard S., Pervin Lawrence A. (1990). Emotion and adaptation. Handbook of Personality: Theory and Research.

[B135-jintelligence-11-00195] Smith Craig A., Lazarus Richard S. (1993). Appraisal components, core relational themes, and the emotions. Cognition and Emotion.

[B136-jintelligence-11-00195] Smith Gregory T. (2005). On construct validity: Issues of method and measurement. Psychological Assessment.

[B137-jintelligence-11-00195] Smith Richard H., Kim Sung H. (2007). Comprehending envy. Psychological Bulletin.

[B138-jintelligence-11-00195] Smith Richard H., Kim Sung H., Parrott W. Gerrod (1988). Envy and jealousy: Semantic problems and experiential distinctions. Personality and Social Psychology Bulletin.

[B139-jintelligence-11-00195] Snyder C. Rick (1995). Conceptualizing, measuring, and nurturing hope. Journal of Counseling Development.

[B140-jintelligence-11-00195] Snyder C. Rick (2002). Hope theory: Rainbows in the mind. Psychological Inquiry.

[B141-jintelligence-11-00195] Stamm B. Hudnall, Figley Charles R. (2002). Measuring compassion satisfaction as well as fatigue: Developmental history of the Compassion Satisfaction and Fatigue Test. Treating Compassion Fatigue.

[B142-jintelligence-11-00195] Steiner Elizabeth D., Woo Ashley (2021). Job-Related Stress Threatens the Teacher Supply: Key Findings from the 2021 State of the U.S Teacher Survey (RR-A1108-1).

[B143-jintelligence-11-00195] Tamir Maya, John Oliver P., Srivastava Sanjay, Gross James J. (2007). Implicit theories of emotion: Affective and social outcomes across a major life transition. Journal of Personality and Social Psychology.

[B144-jintelligence-11-00195] Tangney June P., Dalgleish Tim, Power Mick J. (1999). The self-conscious emotions: Shame, guilt, embarrassment and pride. Handbook of Cognition and Emotion.

[B145-jintelligence-11-00195] Tangney June P., Fischer Kurt W. (1995). Self-Conscious Emotions: The Psychology of Shame, Guilt, Embarrassment, and Pride.

[B146-jintelligence-11-00195] Tangney June P., Stuewig Jeff, Mashek Debra J. (2007). Moral emotions and moral behavior. Annual Review of Psycholology.

[B147-jintelligence-11-00195] Tangney June P., Miller Rowland S., Flicker Laura, Barlow Deborah H. (1996). Are shame, guilt, and embarrassment distinct emotions?. Journal of Personality and Social Psychology.

[B148-jintelligence-11-00195] Thrash Todd M., Elliot Andrew J. (2003). Inspiration as a psychological construct. Journal of Personality and Social Psychology.

[B149-jintelligence-11-00195] Thrash Todd M., Elliot Andrew J. (2004). Inspiration: Core characteristics, component processes, antecedents, and function. Journal of Personality and Social Psychology.

[B150-jintelligence-11-00195] Toivonen Riitta, Kivelä Mikko, Saramäki Jari, Viinikainen Mikko, Vanhatalo Maija, Sams Mikko (2012). Networks of emotion concepts. PLoS ONE.

[B151-jintelligence-11-00195] Tracy Jessica L., Robins Richard W. (2007). The psychological structure of pride: A tale of two facets. Journal of Personality and Social Psychology.

[B152-jintelligence-11-00195] Tracy Jessica L., Robins Richard W. (2014). Conceptual and empirical strengths of the authentic/hubristic model of pride. Emotion.

[B153-jintelligence-11-00195] Tracy Jessica L., Robins Richard W., Tangney June P. (2007). The Self-Conscious Emotions: Theory and Research.

[B154-jintelligence-11-00195] Travers Cheryl, McIntyre Teresa M., McIntyre Scott E., Francis David J. (2017). Current knowledge on the nature, prevalence, sources and potential impact of teacher stress. Educator Stress An Occupational Health Perspective.

[B155-jintelligence-11-00195] Tugade Michele M., Frederickson Barbara L., Barrett Lisa F. (2004). Psychological resilience and positive emotional granularity: Examining the benefits of positive emotions on coping and health. Journal of Personality.

[B156-jintelligence-11-00195] U.S. Bureau of Labor Statistics (U.S. BLS) (2020). Labor Force Statistics from the Current Population Survey (7. Employment Status of the Civilian Noninstitutional Population 25 Years and Over by Educational Attainment, Sex, Race, and Hispanic or Latino Ethnicity). https://www.bls.gov/cps/aa2020/cpsaat07.htm.

[B157-jintelligence-11-00195] U.S. Census Bureau (2020a). Age and Sex Composition in the United States: 2020 (Table 1. Population by Age and Sex: 2020). https://www.census.gov/data/tables/2020/demo/age-and-sex/2020-age-sex-composition.html.

[B158-jintelligence-11-00195] U.S. Census Bureau (2020b). Educational Attainment in the United States: 2020 (Table 1. Educational Attainment of the Population 18 Years and Over, by Age, Sex, Race, and Hispanic Origin: 2020). https://www.census.gov/data/tables/2020/demo/educational-attainment/cps-detailed-tables.html.

[B159-jintelligence-11-00195] U.S. Census Bureau (2020c). Race and Ethnicity in the United States: 2010 Census and 2020 Census. https://www.census.gov/library/visualizations/interactive/race-and-ethnicity-in-the-united-state-2010-and-2020-census.html.

[B160-jintelligence-11-00195] van Rijn Pol, Larrouy-Maestri Pauline (2023). Modeling individual and cross-cultural variation in the mapping of emotions to speech prosody. Nature Human Behavior.

[B161-jintelligence-11-00195] van Tilburg Wijnand A. P., Igou Eric R. (2017). Boredom begs to differ: Differentiation from other negative emotions. Emotion.

[B162-jintelligence-11-00195] Wang Hui, Hall Nathan C., Taxer Jamie L. (2019). Antecedents and consequences of teachers’ emotional labor: A systematic review and meta-analytic investigation. Educational Psychology Review.

[B163-jintelligence-11-00195] Webb Thomas L., Miles Eleanor, Sheeran Paschal (2012). Dealing with feeling: A meta-analysis of the effectiveness of strategies derived from the process model of emotion regulation. Psychological Bulletin.

