# Supplementary material for: Development and Validation of an Ability Measure of Emotion Understanding: The Core Relational Themes of Emotion (CORE) Test
Source: J Intell. 2023 Oct 9;11(10):195. doi: 10.3390/jintelligence11100195 (PMC10607998; doi:10.3390/jintelligence11100195)
Supplement: Supplementary file 1 [file jintelligence-11-00195-s001.zip › jintelligence-2410181-supplementary.pdf]

Supplementary Materials

Table S1

Study 1: Additional EU Ability Measures for Adults (not Developed and Validated in English)

Measure	Citation
Components of Emotion Understanding Test-24 (CEUT-24)	Huyghe, V. E., Hovasapian, A., & Fontaine, J. R. (2022). The scoring challenge of Emotional Intelligence ability tests: A Confirmatory Factor Analysis approach to model substantive and method effects using raw item scores. *Frontiers in Psychology*, *13*, 812525. https://doi.org/10.3389/fpsyg.2022.812525
Empathic Agent Paradigm Test (EAPT)	Hellwig, S., Roberts, R. D., & Schulze, R. (2020). A new approach to assessing emotional understanding. *Psychological Assessment, 32*(7), 649–662. https://doi.org/10.1037/pas0000822
Mobile Emotional Intelligence Test (MEIT)	Sanchez-Gomez, M., & Breso, E. (2019). The Mobile Emotional Intelligence Test (MEIT): An ability test to assess emotional intelligence at work. *Sustainability*, *11*(3), 827. https://doi.org/10.3390/su11030827
QEPro	Haag, C., Bellinghausen, L., & Jilinskaya-Pandey, M. (2023). QEPro: An ability measure of emotional intelligence for managers in a French cultural environment. *Current Psychology*, *42*(5), 4080-4102. https://doi.org/10.1007/s12144-021-01715-6
Theory-Based Test of Emotional Understanding (TBEU)	Hellwig, S., & Schulze, R. (2021). Emotion theories as a scoring rationale for tests of emotional understanding. *Personality and Individual Differences*, *181*, 111034. https://doi.org/10.1016/j.paid.2021.111034
Test of Emotional Intelligence (TEMINT)	Blickle, G., Momm, T., Liu, Y., Witzki, A., & Steinmayr, R. (2011). Construct validation of the Test of Emotional Intelligence (TEMINT): A two-study investigation. *European Journal of Psychological Assessment, 27*(4), 282–289. https://doi.org/10.1027/1015-5759/a000075 <br>Schmidt-Atzert, L., & Bühner, M. (2002). Development of a performance measure of emotional intelligence. In *43rd congress of the German Psychological Society. Humboldt-University, Berlin, Germany.*
Test of Emotional Intelligence (TIE)	Śmieja, M., Orzechowski, J., & Stolarski, M. S. (2014). TIE: An ability test of emotional intelligence. *PLoS One*, *9*(7), e103484. https://doi.org/10.1371/journal.pone.0103484

Study 1: The Confusion Matrix Proportion Index ('Hit Rate')

The proportion index is calculated as follows:

$$pi = \frac{P(k-1)}{1 + P(k-2)}$$

where *P* is the observed hit rate and *k* is the number of response options (i.e., 24). We used *pi* because it made our hit rates comparable across responses to tests with a different number of options (and so the CORE can be compared with other EU ability tests). It also renders judgments about whether the correct answers were selected above chance easier to understand (i.e., any answer selected more than *pi* =.50, or more than 50% of the time, is considered above chance).

Table S2

Study 1: Participant Demographic Characteristics

Demographic Characteristic	% or Mean *(SD)*
**Age**	38.8 *(12.1)*
**Gender**	
Female	55.7
Male	44.3
Non-Binary Identity	0.0
**Race/Ethnicity**	
White	38.0
Latinx	22.5
Asian	19.9
Black	19.6
**Primary Language Spoken**	
English	100
**Education Level**	
High School	28.8
Some College	20.6
Associate Degree	10.8
Bachelor's Degree	24.0
Master's Degree	12.0
Doctoral Degree	3.8
**Job Status**	
Full-time (>30 hours a week)	100
**Job Type**	
Other	29.2
Education or Research	12.4
Construction or Manufacturing	12.3
Business or Finance	11.0
Managerial	9.9
Tech Industry	8.5
Service Sector	7.6
Law or Medicine	6.9
Not currently employed	2.2

Note. $N = 684$.

Table S3

Study 1: Item-Level Raw Hit Rate (Item Difficulty) on the CORE Test (Confusion Matrix)

Item	amu	awe	con	gra	hop	ins	int	joy	lov	pro	rel	ang	anx	ash	bor	dis	emb	env	fea	gui	jea	sad	com	sur
amused1	49.3%	0.6%	2.9%	1.2%	0.9%	1.3%	2.6%	29.2%	0.7%	0.7%	0.3%	0.6%	1.3%	0.3%	0.7%	0.3%	0.6%	0.6%	0.4%	0.1%	1.0%	0.9%	1.8%	1.6%
amused2	25.7%	3.2%	3.8%	0.9%	0.9%	0.6%	4.7%	0.3%	0.0%	0.1%	6.0%	3.8%	6.4%	3.2%	3.1%	13.9%	3.5%	0.9%	3.1%	0.6%	0.0%	1.9%	1.8%	11.8%
amused3	15.4%	2.2%	1.6%	0.4%	0.3%	0.3%	1.0%	0.7%	0.4%	0.6%	2.3%	9.6%	3.2%	9.1%	1.6%	20.8%	14.8%	0.9%	1.5%	1.8%	0.7%	3.7%	1.5%	5.7%
amused4	16.5%	1.8%	2.6%	0.3%	0.6%	1.2%	2.2%	2.3%	0.7%	0.7%	0.6%	18.1%	3.4%	6.9%	1.2%	16.7%	7.3%	1.5%	1.3%	1.8%	0.6%	3.9%	1.8%	6.1%
amused5	26.3%	1.3%	0.9%	0.3%	0.3%	0.1%	0.4%	1.3%	0.3%	0.4%	1.8%	2.3%	1.9%	7.2%	0.6%	3.5%	34.4%	0.9%	1.3%	0.7%	0.6%	1.8%	8.2%	3.2%
awe1	2.8%	40.8%	1.3%	2.2%	2.0%	15.1%	2.3%	1.5%	0.6%	2.8%	0.7%	0.6%	5.0%	2.2%	0.7%	0.7%	2.2%	4.7%	5.1%	0.4%	2.5%	0.4%	1.9%	1.3%
awe2	2.5%	35.5%	1.3%	2.3%	3.4%	13.6%	5.0%	1.3%	0.1%	3.4%	1.0%	0.3%	6.4%	1.2%	0.9%	0.4%	0.4%	2.3%	11.5%	0.3%	1.0%	0.1%	2.3%	3.2%
awe3	4.1%	39.0%	0.6%	1.0%	0.7%	2.3%	1.3%	2.2%	0.7%	1.3%	1.0%	0.6%	3.9%	2.0%	1.6%	1.2%	2.6%	0.7%	1.3%	0.4%	0.3%	0.1%	1.2%	29.5%
content1	1.2%	1.5%	64.6%	6.9%	1.5%	0.6%	0.4%	3.1%	0.9%	1.8%	10.1%	0.1%	0.6%	0.4%	2.9%	0.0%	0.1%	0.7%	0.4%	0.0%	0.3%	0.3%	0.7%	0.9%
content2	0.9%	0.4%	60.1%	5.0%	3.7%	1.5%	0.7%	2.2%	1.5%	2.3%	10.5%	0.4%	0.6%	1.2%	1.3%	0.7%	0.9%	0.3%	0.7%	0.6%	0.3%	0.4%	2.8%	1.0%
content3	0.9%	1.0%	50.0%	9.5%	1.3%	1.5%	0.4%	9.1%	1.2%	9.8%	8.2%	0.1%	1.0%	0.3%	0.7%	0.4%	1.2%	0.3%	0.3%	0.3%	0.1%	0.6%	1.5%	0.3%
grateful1	0.6%	3.8%	2.3%	51.2%	1.2%	1.2%	0.6%	5.8%	13.6%	1.3%	1.3%	0.6%	0.3%	0.6%	0.7%	0.7%	1.6%	0.3%	1.2%	0.6%	0.7%	0.0%	5.3%	4.5%
grateful2	2.0%	1.5%	9.2%	40.6%	3.8%	1.9%	2.0%	16.1%	2.0%	6.0%	3.2%	0.1%	1.2%	0.7%	1.2%	0.7%	0.6%	1.0%	0.7%	0.3%	0.3%	0.3%	1.3%	3.1%
grateful3	1.0%	1.9%	9.8%	48.0%	3.9%	2.6%	1.3%	7.5%	1.0%	10.2%	3.4%	0.7%	1.0%	1.0%	0.6%	0.6%	1.2%	0.0%	0.9%	0.4%	0.3%	0.3%	1.6%	0.7%
grateful4	1.8%	1.3%	9.9%	30.8%	1.0%	1.5%	0.3%	3.8%	0.6%	21.5%	1.8%	0.4%	1.0%	2.5%	0.9%	1.2%	2.3%	4.1%	0.1%	7.0%	2.3%	0.9%	2.0%	0.9%
hopeful1	1.6%	2.5%	5.3%	5.3%	20.3%	32.7%	1.6%	6.3%	0.4%	7.6%	5.3%	0.7%	2.9%	0.4%	0.4%	0.4%	0.4%	0.7%	0.6%	0.3%	0.1%	0.0%	2.3%	1.6%
hopeful2	0.7%	1.2%	2.2%	4.5%	39.3%	26.9%	5.3%	2.8%	0.3%	2.5%	3.1%	0.1%	3.1%	0.6%	0.9%	0.6%	0.4%	0.9%	0.3%	0.1%	0.6%	0.3%	2.6%	0.7%
hopeful3	1.9%	1.5%	6.6%	3.5%	52.9%	4.2%	1.0%	5.3%	0.4%	1.8%	4.7%	0.6%	7.7%	0.4%	0.7%	0.6%	0.4%	0.7%	1.2%	0.3%	0.6%	0.0%	1.2%	1.8%
hopeful4	0.9%	1.5%	3.1%	1.9%	61.7%	8.0%	1.2%	1.3%	0.3%	0.6%	2.3%	0.4%	4.7%	1.2%	0.6%	1.2%	0.9%	1.5%	1.6%	0.9%	0.1%	0.6%	2.5%	1.2%
inspired1	1.8%	14.6%	2.5%	3.8%	3.7%	36.1%	1.0%	2.2%	1.0%	14.5%	1.0%	0.3%	1.3%	1.3%	0.7%	1.0%	0.3%	2.6%	0.4%	0.4%	0.9%	0.4%	5.8%	2.2%

Item	amu	awe	con	gra	hop	ins	int	joy	lov	pro	rel	ang	anx	ash	bor	dis	emb	env	fea	gui	jea	sad	com	sur
inspired2	1.5%	5.7%	2.2%	4.7%	5.1%	32.9%	1.5%	4.7%	0.3%	15.1%	4.5%	1.2%	1.6%	1.3%	1.0%	0.7%	0.7%	2.0%	1.8%	0.4%	1.8%	2.0%	5.3%	2.0%
interested1	6.9%	6.0%	4.5%	2.3%	2.9%	7.6%	51.5%	2.0%	0.9%	1.2%	1.0%	0.4%	2.6%	0.7%	1.3%	0.4%	0.6%	0.9%	0.9%	0.1%	0.3%	0.6%	2.0%	2.2%
interested2	9.4%	8.0%	1.5%	1.5%	2.8%	4.8%	35.5%	2.6%	0.3%	2.2%	0.6%	0.4%	6.0%	1.0%	0.7%	1.3%	0.6%	0.9%	1.2%	0.1%	0.7%	0.3%	0.7%	16.8%
joy1	3.2%	1.3%	14.9%	13.5%	9.6%	3.8%	0.6%	27.3%	1.3%	7.2%	7.9%	0.3%	0.7%	0.6%	0.7%	0.7%	0.4%	0.4%	0.7%	0.0%	0.6%	0.7%	1.8%	1.6%
joy2	3.4%	1.8%	5.6%	13.2%	3.1%	2.2%	0.6%	46.3%	1.2%	4.1%	6.3%	0.1%	1.3%	0.9%	0.4%	0.4%	0.4%	0.3%	0.4%	0.1%	0.1%	0.0%	2.2%	5.6%
joy3	13.9%	1.3%	8.9%	5.4%	1.9%	3.9%	8.0%	37.1%	1.8%	0.9%	4.8%	0.1%	2.2%	0.7%	1.3%	0.9%	0.4%	1.0%	0.7%	0.3%	0.9%	0.4%	1.9%	1.0%
joy4	17.4%	1.3%	6.7%	1.8%	1.5%	1.0%	4.4%	53.7%	0.3%	2.3%	1.0%	0.1%	0.6%	0.6%	0.4%	0.9%	0.6%	0.6%	0.3%	0.7%	0.7%	0.0%	1.8%	1.3%
loved1	1.0%	1.9%	11.8%	15.4%	1.5%	2.0%	0.9%	5.4%	38.9%	2.6%	8.0%	0.1%	0.6%	0.4%	1.0%	0.9%	0.4%	0.6%	0.1%	0.3%	0.0%	0.3%	4.8%	0.9%
loved2	1.5%	4.7%	2.2%	13.6%	0.9%	6.4%	9.1%	8.0%	26.2%	5.0%	0.6%	0.9%	1.8%	0.9%	1.2%	0.3%	1.8%	1.0%	0.6%	0.3%	0.3%	0.4%	10.7%	1.9%
loved3	0.9%	2.2%	4.0%	33.5%	1.9%	1.0%	0.0%	1.9%	37.9%	1.3%	4.1%	0.1%	0.1%	1.8%	0.7%	1.0%	0.7%	0.4%	0.4%	0.3%	0.1%	0.4%	4.5%	0.4%
loved4	2.8%	5.0%	6.4%	13.2%	0.6%	4.4%	5.6%	4.2%	32.2%	4.2%	1.9%	0.3%	1.5%	1.8%	1.2%	0.7%	3.2%	2.0%	0.3%	0.3%	1.6%	0.4%	3.4%	2.9%
loved5	1.8%	3.1%	2.9%	22.5%	8.0%	6.0%	2.2%	2.0%	30.6%	3.7%	1.2%	0.7%	1.3%	1.0%	0.4%	0.7%	1.0%	0.4%	1.2%	0.6%	0.9%	0.4%	6.0%	1.3%
proud1	0.6%	1.0%	7.6%	7.7%	3.9%	6.3%	0.3%	2.6%	0.1%	57.7%	2.9%	0.9%	0.4%	0.9%	1.3%	0.7%	0.3%	0.3%	0.3%	0.6%	0.0%	0.4%	2.5%	0.4%
proud2	1.5%	1.3%	8.0%	6.9%	1.2%	5.8%	1.0%	11.3%	0.3%	47.4%	6.9%	0.4%	1.3%	0.4%	0.6%	1.5%	0.1%	0.9%	0.3%	0.1%	0.1%	0.0%	1.2%	1.5%
proud3	5.1%	2.5%	5.3%	1.3%	0.3%	2.5%	0.3%	2.0%	1.3%	44.2%	1.8%	1.9%	1.3%	3.8%	0.9%	2.3%	1.5%	9.4%	0.0%	3.1%	6.4%	0.4%	1.9%	0.6%
proud4	2.2%	3.8%	8.9%	4.8%	2.9%	6.9%	2.9%	5.1%	6.9%	36.5%	1.5%	0.4%	2.2%	1.9%	0.9%	0.1%	1.5%	3.4%	0.9%	0.3%	0.6%	0.3%	3.5%	1.6%
relieved1	1.3%	2.0%	2.0%	10.7%	1.5%	0.7%	0.0%	2.8%	0.4%	0.0%	54.8%	2.6%	3.2%	1.0%	1.6%	2.5%	1.3%	0.1%	1.5%	1.2%	0.4%	4.2%	1.2%	2.8%
relieved2	1.6%	0.7%	3.1%	10.1%	2.2%	0.6%	0.7%	4.8%	0.3%	1.6%	56.3%	2.9%	1.9%	1.2%	1.0%	1.6%	1.2%	0.7%	0.7%	0.7%	0.7%	4.1%	0.7%	0.4%
relieved3	2.0%	2.0%	4.5%	11.0%	2.0%	0.3%	1.0%	1.9%	0.4%	1.0%	52.0%	0.7%	1.9%	1.0%	1.5%	1.6%	0.7%	0.7%	0.3%	0.1%	0.4%	0.9%	1.2%	10.5%
angry1	0.4%	1.0%	2.0%	1.5%	1.8%	1.6%	1.0%	0.7%	0.7%	1.2%	0.7%	50.3%	11.1%	1.3%	1.0%	5.7%	0.4%	4.2%	2.6%	0.6%	1.9%	6.0%	0.9%	1.2%
angry2	0.6%	0.9%	0.4%	0.1%	0.3%	0.6%	0.3%	0.3%	0.1%	0.6%	1.0%	55.4%	2.6%	2.6%	0.9%	10.2%	0.7%	3.2%	1.3%	1.2%	3.4%	10.8%	1.3%	1.0%
angry3	0.6%	0.7%	0.6%	0.7%	0.3%	0.1%	0.0%	0.6%	0.3%	0.3%	1.2%	43.6%	2.0%	1.8%	0.6%	4.5%	1.0%	0.9%	21.8%	1.0%	0.006	15.4%	0.4%	1.0%
angry4	0.9%	1.5%	1.2%	0.6%	0.1%	0.4%	0.0%	0.3%	0.7%	0.7%	0.4%	61.3%	1.6%	2.9%	0.9%	9.5%	4.4%	0.6%	1.0%	0.6%	0.3%	6.9%	1.2%	2.0%

Item	amu	awe	con	gra	hop	ins	int	joy	lov	pro	rel	ang	anx	ash	bor	dis	emb	env	fea	gui	jea	sad	com	sur
anxious1	0.6%	2.6%	2.3%	0.7%	3.9%	0.3%	1.5%	0.3%	0.1%	0.3%	0.4%	1.3%	61.5%	1.3%	2.0%	1.9%	0.4%	1.0%	11.3%	0.6%	0.4%	1.9%	0.4%	2.6%
anxious2	0.7%	0.9%	0.4%	0.1%	0.7%	0.3%	0.1%	0.0%	0.4%	0.3%	0.3%	3.7%	50.0%	2.5%	1.2%	1.9%	1.3%	0.3%	23.8%	1.9%	0.3%	6.6%	1.2%	1.0%
anxious3	0.4%	1.2%	0.9%	0.6%	0.6%	0.1%	1.5%	0.3%	0.4%	0.3%	0.7%	2.0%	52.6%	2.2%	0.7%	2.6%	2.5%	1.2%	23.0%	1.8%	0.4%	1.8%	1.6%	0.6%
ashamed1	1.0%	1.2%	1.8%	0.1%	0.4%	0.7%	0.6%	0.6%	0.7%	1.5%	0.9%	5.6%	5.6%	41.8%	0.7%	9.2%	8.9%	1.3%	1.3%	9.2%	0.6%	3.5%	1.3%	1.5%
ashamed2	1.0%	1.6%	1.8%	0.6%	0.7%	1.8%	0.3%	0.0%	0.6%	1.2%	0.4%	2.2%	2.8%	44.4%	1.5%	8.2%	11.8%	0.9%	0.9%	5.7%	0.3%	9.5%	1.0%	0.9%
ashamed3	0.9%	1.5%	2.3%	0.6%	0.9%	0.3%	0.3%	0.4%	0.7%	0.9%	1.2%	2.3%	1.6%	52.3%	0.9%	5.6%	8.6%	0.9%	0.9%	7.3%	0.7%	7.2%	1.3%	0.4%
bored1	0.6%	1.3%	6.6%	1.2%	1.5%	0.6%	0.3%	0.1%	0.4%	0.0%	2.3%	3.8%	10.2%	2.0%	48.4%	4.1%	1.8%	1.0%	2.0%	0.7%	0.4%	8.2%	1.5%	0.9%
bored2	2.2%	2.3%	9.2%	1.2%	1.2%	1.0%	2.9%	0.4%	0.3%	0.6%	3.5%	1.0%	3.7%	1.6%	58.2%	1.2%	0.7%	0.9%	0.9%	0.7%	0.4%	2.8%	6.0%	2.5%
bored3	2.6%	2.2%	4.1%	0.4%	0.3%	0.6%	1.3%	0.3%	0.1%	0.0%	2.3%	2.3%	4.1%	1.8%	60.8%	2.6%	1.6%	1.5%	0.9%	1.5%	0.9%	2.2%	1.3%	4.2%
bored4	2.9%	1.0%	4.8%	2.3%	0.6%	2.0%	2.3%	2.8%	0.3%	3.5%	9.6%	0.4%	2.2%	1.3%	52.2%	0.3%	1.3%	1.8%	0.3%	0.9%	0.7%	0.3%	1.0%	5.0%
disgusted1	0.6%	0.7%	0.4%	0.6%	0.1%	0.4%	0.4%	0.3%	0.3%	0.6%	0.4%	11.5%	10.7%	2.8%	1.6%	34.6%	0.9%	0.7%	25.7%	1.0%	0.4%	3.8%	0.3%	0.9%
disgusted2	1.3%	1.3%	0.7%	0.3%	1.0%	0.4%	0.1%	0.4%	0.0%	0.6%	0.7%	1.5%	0.9%	2.5%	0.9%	78.2%	1.8%	0.9%	1.5%	0.9%	0.1%	1.3%	0.7%	1.9%
disgusted3	1.5%	1.2%	0.9%	0.7%	0.4%	0.3%	0.1%	0.3%	0.1%	1.0%	0.9%	23.7%	1.9%	13.2%	1.2%	35.1%	3.8%	1.0%	2.5%	1.5%	0.4%	3.9%	1.0%	3.4%
disgusted4	1.5%	1.9%	1.2%	0.6%	0.1%	0.4%	0.7%	0.1%	0.6%	0.7%	0.6%	18.7%	2.0%	12.1%	0.4%	31.6%	18.0%	0.4%	0.7%	1.0%	0.3%	2.8%	0.9%	2.5%
disgusted5	1.6%	2.2%	2.9%	0.4%	0.7%	0.0%	0.4%	0.4%	0.7%	0.6%	0.9%	22.4%	4.5%	1.2%	2.9%	39.9%	2.6%	3.4%	1.0%	1.5%	2.5%	5.0%	2.6%	1.2%
embarrassed1	1.3%	1.2%	1.2%	0.4%	0.7%	0.4%	0.3%	0.7%	0.1%	0.6%	0.7%	0.3%	3.2%	16.7%	0.7%	1.3%	65.5%	0.4%	1.0%	0.1%	0.0%	0.6%	1.6%	0.7%
embarrassed2	0.6%	0.9%	0.9%	0.9%	0.7%	0.7%	0.0%	0.3%	0.4%	0.7%	0.9%	8.6%	4.8%	20.2%	1.5%	1.9%	48.2%	0.7%	1.8%	0.7%	0.6%	1.3%	0.9%	1.8%
embarrassed3	2.3%	4.5%	1.8%	6.4%	0.6%	3.2%	0.4%	3.1%	3.2%	6.6%	0.7%	2.5%	3.1%	4.5%	1.0%	3.4%	37.4%	1.5%	0.4%	1.6%	0.7%	1.8%	1.5%	7.7%
envy1	0.7%	1.0%	1.8%	0.4%	3.2%	1.9%	2.5%	0.1%	0.6%	1.0%	1.3%	4.7%	3.4%	0.9%	1.3%	1.3%	1.0%	38.5%	0.7%	0.7%	30.1%	0.7%	1.2%	0.9%
envy2	1.5%	2.9%	1.5%	2.2%	1.5%	3.4%	4.2%	1.5%	0.7%	1.3%	0.3%	0.6%	1.3%	0.7%	0.1%	0.9%	0.9%	41.3%	0.3%	0.3%	29.1%	0.1%	2.0%	1.3%
envy3	1.2%	1.5%	1.6%	0.4%	12.3%	2.5%	2.0%	0.3%	0.4%	0.6%	0.9%	0.6%	3.9%	1.3%	1.0%	1.0%	0.6%	41.4%	0.4%	1.8%	17.7%	5.0%	1.3%	0.3%
fear1	0.6%	0.7%	1.2%	0.4%	0.4%	0.6%	0.3%	0.3%	0.0%	0.7%	0.0%	8.8%	15.9%	1.6%	1.3%	1.3%	0.7%	1.2%	58.5%	0.1%	1.3%	1.6%	0.9%	1.5%
fear2	0.7%	0.6%	1.0%	0.6%	0.4%	0.4%	0.1%	0.6%	0.1%	1.2%	1.2%	5.0%	13.7%	1.0%	0.7%	1.0%	0.9%	0.7%	62.5%	0.4%	0.1%	3.1%	1.6%	2.0%

Item	amu	awe	con	gra	hop	ins	int	joy	lov	pro	rel	ang	anx	ash	bor	dis	emb	env	fea	gui	jea	sad	com	sur
guilty1	0.6%	1.2%	1.2%	0.3%	0.4%	0.3%	0.6%	0.6%	0.6%	0.7%	0.9%	4.2%	1.8%	31.9%	0.4%	1.9%	5.0%	0.3%	1.6%	34.2%	0.3%	8.3%	1.9%	0.9%
guilty2	0.3%	0.7%	1.5%	0.3%	0.1%	0.0%	0.3%	0.3%	0.3%	0.4%	0.1%	5.3%	2.5%	39.8%	1.0%	4.5%	25.1%	0.3%	0.9%	10.8%	0.7%	1.6%	0.9%	2.2%
jealous1	1.0%	1.8%	0.9%	0.9%	0.6%	1.0%	0.4%	0.6%	0.9%	0.6%	0.6%	3.9%	1.8%	1.0%	0.6%	1.6%	1.5%	16.5%	1.6%	0.4%	49.7%	9.1%	2.0%	1.0%
jealous2	0.3%	1.0%	1.8%	0.7%	0.4%	0.7%	0.1%	0.9%	1.0%	0.4%	0.4%	4.5%	1.0%	1.0%	3.4%	0.9%	1.5%	14.5%	1.2%	0.3%	56.6%	4.2%	1.5%	1.6%
jealous3	1.5%	1.0%	0.7%	1.8%	0.9%	0.6%	2.5%	0.9%	4.2%	0.9%	0.9%	2.2%	1.9%	2.2%	1.0%	1.2%	1.3%	18.0%	0.6%	0.1%	49.9%	1.9%	3.1%	0.9%
sad1	0.7%	1.5%	1.0%	0.0%	0.1%	0.6%	0.6%	0.3%	0.7%	0.3%	0.6%	23.0%	4.1%	5.6%	1.0%	5.8%	2.9%	1.0%	0.4%	1.8%	1.0%	42.3%	0.6%	4.1%
sad2	0.7%	0.6%	0.7%	0.7%	0.4%	0.3%	0.3%	0.3%	0.4%	0.4%	0.9%	7.7%	2.2%	0.9%	1.5%	2.0%	0.7%	0.6%	2.2%	1.3%	0.7%	71.8%	1.3%	1.2%
compassionate1	0.9%	1.5%	0.6%	0.0%	0.3%	0.7%	0.4%	0.0%	0.1%	0.6%	1.0%	1.5%	1.9%	0.9%	1.0%	1.5%	0.4%	0.7%	3.8%	3.8%	0.1%	30.0%	48.1%	0.1%
compassionate2	1.2%	0.9%	1.0%	3.9%	3.4%	4.1%	4.7%	0.3%	1.6%	2.8%	1.6%	1.2%	3.1%	1.5%	1.3%	1.0%	1.9%	0.7%	0.4%	0.9%	0.1%	2.3%	59.6%	0.4%
compassionate3	0.3%	1.5%	1.3%	3.0%	7.0%	3.0%	7.0%	7.0%	1.0%	3.0%	7.0%	17.1%	2.6%	3.2%	1.5%	5.4%	1.8%	1.2%	1.0%	4.2%	7.0%	22.4%	30.8%	1.0%
surprised1	1.0%	3.2%	0.4%	1.2%	1.2%	0.9%	1.2%	0.6%	0.4%	0.6%	1.2%	9.0%	10.8%	1.5%	0.9%	1.5%	0.7%	0.6%	2.2%	0.6%	0.0%	1.2%	0.9%	66.5%
surprised2	1.8%	2.8%	0.9%	1.3%	1.5%	0.9%	0.7%	0.6%	0.7%	0.6%	0.9%	0.7%	6.9%	0.9%	1.3%	1.2%	0.9%	0.3%	4.5%	0.3%	0.0%	0.1%	0.9%	69.0%

Note: Greyed cells indicate hit rates for target responses.

Table S4

Study 1: Emotion-Level Raw Hit Rate (Item Difficulty) on the CORE Test (Confusion Matrix)

Emotion	amu	awe	con	gra	hop	ins	int	joy	lov	pro	Rel	ang	anx	ash	bor	dis	emb	env	fea	gui	jea	sad	com	sur
amused	26.6%	1.8%	2.4%	0.6%	0.6%	0.7%	2.2%	6.8%	0.4%	0.5%	2.2%	6.9%	3.2%	5.3%	1.4%	11.0%	12.1%	1.0%	1.5%	1.0%	0.6%	2.4%	3.0%	5.7%
awed	3.1%	38.4%	1.1%	1.8%	2.0%	10.3%	2.9%	1.7%	0.5%	2.5%	0.9%	0.5%	5.1%	1.8%	1.1%	0.8%	1.7%	2.6%	6.0%	0.4%	1.3%	0.2%	1.8%	11.3%
content	1.0%	1.0%	58.2%	7.1%	2.2%	1.2%	0.5%	4.8%	1.2%	4.6%	9.6%	0.2%	0.7%	0.6%	1.6%	0.4%	0.7%	0.4%	0.5%	0.3%	0.2%	0.4%	1.7%	0.7%
grateful	1.4%	2.1%	7.8%	42.7%	2.5%	1.8%	1.1%	8.3%	4.3%	9.8%	2.4%	0.5%	0.9%	1.2%	0.9%	0.8%	1.4%	1.4%	0.7%	2.1%	0.9%	0.4%	2.6%	2.3%
hopeful	1.3%	1.7%	4.3%	3.8%	43.6%	18.0%	2.3%	3.9%	0.4%	3.1%	3.9%	0.5%	4.6%	0.7%	0.7%	0.7%	0.5%	1.0%	0.9%	0.4%	0.4%	0.2%	2.2%	1.3%
inspired	1.7%	10.2%	2.4%	4.3%	4.4%	34.5%	1.3%	3.5%	0.7%	14.8%	2.8%	0.8%	1.5%	1.3%	0.9%	0.9%	0.5%	2.3%	1.1%	0.4%	1.4%	1.2%	5.6%	2.1%
interested	8.2%	7.0%	3.0%	1.9%	2.9%	6.2%	43.5%	2.3%	0.6%	1.7%	0.8%	0.4%	4.3%	0.9%	1.0%	0.9%	0.6%	0.9%	1.1%	0.1%	0.5%	0.5%	1.4%	9.5%
joyful	9.5%	1.4%	9.0%	8.5%	4.0%	2.7%	3.4%	41.1%	1.2%	3.6%	5.0%	0.2%	1.2%	0.7%	0.7%	0.7%	0.5%	0.6%	0.5%	0.3%	0.6%	0.3%	1.9%	2.4%
loved	1.6%	3.4%	5.5%	19.6%	2.6%	4.0%	3.6%	4.3%	33.2%	3.4%	3.2%	0.4%	1.1%	1.2%	0.9%	0.7%	1.4%	0.9%	0.5%	0.4%	0.6%	0.4%	5.9%	1.5%
proud	2.4%	2.2%	7.5%	5.2%	2.1%	5.4%	1.1%	5.3%	2.2%	46.5%	3.3%	0.9%	1.3%	1.8%	0.9%	1.2%	0.9%	3.5%	0.4%	1.0%	1.8%	0.3%	2.3%	1.0%
relieved	1.6%	1.6%	3.2%	10.6%	1.9%	0.5%	0.6%	3.2%	0.4%	0.9%	54.4%	2.1%	2.3%	1.1%	1.4%	1.9%	1.1%	0.5%	0.8%	0.7%	0.5%	3.1%	1.0%	4.6%
angry	0.6%	1.0%	1.1%	0.7%	0.6%	0.7%	0.3%	0.5%	0.5%	0.7%	0.8%	52.7%	4.3%	2.2%	0.9%	7.5%	1.6%	2.2%	6.7%	0.9%	1.6%	9.8%	1.0%	1.3%
anxious	0.6%	1.6%	1.2%	0.5%	1.7%	0.2%	1.0%	0.2%	0.3%	0.3%	0.5%	2.3%	54.7%	2.0%	1.3%	2.1%	1.4%	0.8%	19.4%	1.4%	0.4%	3.4%	1.1%	1.4%
ashamed	1.0%	1.4%	2.0%	0.4%	0.7%	0.9%	0.4%	0.3%	0.7%	1.2%	0.8%	3.4%	3.3%	46.2%	1.0%	7.7%	9.8%	1.0%	1.0%	7.4%	0.5%	6.7%	1.2%	0.9%
bored	2.1%	1.7%	6.2%	1.3%	0.9%	1.1%	1.7%	0.9%	0.3%	1.0%	4.4%	1.9%	5.1%	1.7%	54.9%	2.1%	1.4%	1.3%	1.0%	1.0%	0.6%	3.4%	2.5%	3.2%
disgusted	1.3%	1.5%	1.2%	0.5%	0.5%	0.3%	0.3%	0.3%	0.3%	0.7%	0.7%	15.6%	4.0%	6.4%	1.4%	43.9%	5.4%	1.3%	6.3%	1.2%	0.7%	3.4%	1.1%	2.0%
embarrassed	1.4%	2.2%	1.3%	2.6%	0.7%	1.4%	0.2%	1.4%	1.2%	2.6%	0.8%	3.8%	3.7%	13.8%	1.1%	2.2%	50.4%	0.9%	1.1%	0.8%	0.4%	1.2%	1.3%	3.4%
envious	1.1%	1.8%	1.6%	1.0%	5.7%	2.6%	2.9%	0.6%	0.6%	1.0%	0.8%	2.0%	2.9%	1.0%	0.8%	1.1%	0.8%	40.4%	0.5%	0.9%	25.6%	1.9%	1.5%	0.8%
fearful	0.7%	0.7%	1.1%	0.5%	0.4%	0.5%	0.2%	0.5%	0.1%	1.0%	0.6%	6.9%	14.8%	1.3%	1.0%	1.2%	0.8%	1.0%	60.5%	0.3%	0.7%	2.4%	1.3%	1.8%
guilty	0.5%	1.0%	1.4%	0.3%	0.3%	0.2%	0.5%	0.5%	0.5%	0.6%	0.5%	4.8%	2.2%	35.9%	0.7%	3.2%	15.1%	0.3%	1.3%	22.5%	0.5%	5.0%	1.4%	1.6%

Emotion	amu	awe	con	gra	hop	ins	int	joy	lov	pro	Rel	ang	anx	ash	bor	dis	emb	env	fea	gui	jea	sad	com	sur
jealous	0.9%	1.3%	1.1%	1.1%	0.6%	0.8%	1.0%	0.8%	2.0%	0.6%	0.6%	3.5%	1.6%	1.4%	1.7%	1.2%	1.4%	16.3%	1.1%	0.3%	52.1%	5.1%	2.2%	1.2%
sad	0.7%	1.1%	0.9%	0.4%	0.3%	0.5%	0.5%	0.3%	0.6%	0.4%	0.8%	15.4%	3.2%	3.3%	1.3%	3.9%	1.8%	0.8%	1.3%	1.6%	0.9%	57.1%	1.0%	2.7%
compassionate	0.8%	1.3%	1.0%	2.3%	3.6%	2.6%	4.0%	2.4%	0.9%	2.1%	3.2%	6.6%	2.5%	1.9%	1.3%	2.6%	1.4%	0.9%	1.7%	3.0%	2.4%	18.2%	46.2%	0.5%
surprised	1.4%	3.0%	0.7%	1.3%	1.4%	0.9%	1.0%	0.6%	0.6%	0.6%	1.1%	4.9%	8.9%	1.2%	1.1%	1.4%	0.8%	0.5%	3.4%	0.5%	0.0%	0.7%	0.9%	67.8%

Note: Greyed cells indicate hit rates for target responses.

Table S5

Study 1: Item-Level Chance-Adjusted Hit Rate (Item Difficulty) on the CORE Test (Confusion Matrix)

Item	amu	awe	con	gra	hop	ins	int	joy	lov	pro	rel	ang	anx	ash	bor	dis	emb	env	fea	gui	jea	sad	com	sur
amused1	0.96	0.12	0.40	0.21	0.17	0.22	0.37	0.90	0.13	0.13	0.06	0.12	0.22	0.06	0.13	0.06	0.12	0.12	0.08	0.02	0.18	0.17	0.29	0.26
amused2	0.88	0.42	0.46	0.17	0.17	0.12	0.52	0.06	0.00	0.02	0.58	0.46	0.60	0.42	0.41	0.78	0.44	0.17	0.41	0.12	0.00	0.30	0.29	0.75
amused3	0.80	0.33	0.26	0.08	0.06	0.06	0.18	0.13	0.08	0.12	0.34	0.70	0.42	0.69	0.26	0.85	0.79	0.17	0.25	0.29	0.13	0.46	0.25	0.57
amused4	0.81	0.29	0.37	0.06	0.12	0.21	0.33	0.34	0.13	0.13	0.12	0.83	0.44	0.62	0.21	0.82	0.63	0.25	0.22	0.29	0.12	0.47	0.29	0.59
amused5	0.89	0.22	0.17	0.06	0.06	0.02	0.08	0.22	0.06	0.08	0.29	0.34	0.30	0.63	0.12	0.44	0.92	0.17	0.22	0.13	0.12	0.29	0.66	0.42
awe1	0.39	0.94	0.22	0.33	0.31	0.80	0.34	0.25	0.12	0.39	0.13	0.12	0.54	0.33	0.13	0.13	0.33	0.52	0.54	0.08	0.36	0.08	0.30	0.22
awe2	0.36	0.92	0.22	0.34	0.44	0.78	0.54	0.22	0.02	0.44	0.18	0.06	0.60	0.21	0.17	0.08	0.08	0.34	0.74	0.06	0.18	0.02	0.34	0.42
awe3	0.48	0.93	0.12	0.18	0.13	0.34	0.22	0.33	0.13	0.22	0.18	0.12	0.47	0.31	0.26	0.21	0.37	0.13	0.22	0.08	0.06	0.02	0.21	0.90
content1	0.21	0.25	0.98	0.62	0.25	0.12	0.08	0.41	0.17	0.29	0.71	0.02	0.12	0.08	0.40	0.00	0.02	0.13	0.08	0.00	0.06	0.06	0.13	0.17
content2	0.17	0.08	0.97	0.54	0.46	0.25	0.13	0.33	0.25	0.34	0.72	0.08	0.12	0.21	0.22	0.13	0.17	0.06	0.13	0.12	0.06	0.08	0.39	0.18
content3	0.17	0.18	0.96	0.70	0.22	0.25	0.08	0.69	0.21	0.71	0.66	0.02	0.18	0.06	0.13	0.08	0.21	0.06	0.06	0.06	0.02	0.12	0.25	0.06
grateful1	0.12	0.46	0.34	0.96	0.21	0.21	0.12	0.58	0.78	0.22	0.22	0.12	0.06	0.12	0.13	0.13	0.26	0.06	0.21	0.12	0.13	0.00	0.55	0.51
grateful2	0.31	0.25	0.69	0.94	0.46	0.30	0.31	0.81	0.31	0.58	0.42	0.02	0.21	0.13	0.21	0.13	0.12	0.18	0.13	0.06	0.06	0.06	0.22	0.41
grateful3	0.18	0.30	0.71	0.95	0.47	0.37	0.22	0.64	0.18	0.71	0.44	0.13	0.18	0.18	0.12	0.12	0.21	0.00	0.17	0.08	0.06	0.06	0.26	0.13
grateful4	0.29	0.22	0.71	0.91	0.18	0.25	0.06	0.46	0.12	0.86	0.29	0.08	0.18	0.36	0.17	0.21	0.34	0.48	0.02	0.62	0.34	0.17	0.31	0.17
hopeful1	0.26	0.36	0.55	0.55	0.85	0.91	0.26	0.60	0.08	0.64	0.55	0.13	0.40	0.08	0.08	0.08	0.08	0.13	0.12	0.06	0.02	0.00	0.34	0.26
hopeful2	0.13	0.21	0.33	0.51	0.93	0.89	0.55	0.39	0.06	0.36	0.41	0.02	0.41	0.12	0.17	0.12	0.08	0.17	0.06	0.02	0.12	0.06	0.37	0.13
hopeful3	0.30	0.25	0.61	0.44	0.96	0.49	0.18	0.55	0.08	0.29	0.52	0.12	0.65	0.08	0.13	0.12	0.08	0.13	0.21	0.06	0.12	0.00	0.21	0.29
hopeful4	0.17	0.25	0.41	0.30	0.97	0.66	0.21	0.22	0.06	0.12	0.34	0.08	0.52	0.21	0.12	0.21	0.17	0.25	0.26	0.17	0.02	0.12	0.36	0.21
inspired1	0.29	0.79	0.36	0.46	0.46	0.93	0.18	0.33	0.18	0.79	0.18	0.06	0.22	0.22	0.13	0.18	0.06	0.37	0.08	0.08	0.17	0.08	0.58	0.33

Item	amu	awe	con	gra	hop	ins	int	joy	lov	pro	rel	ang	anx	ash	bor	dis	emb	env	fea	gui	jea	sad	com	sur
inspired2	0.25	0.57	0.33	0.52	0.54	0.92	0.25	0.52	0.06	0.80	0.51	0.21	0.26	0.22	0.18	0.13	0.13	0.31	0.29	0.08	0.29	0.31	0.55	0.31
interested1	0.62	0.58	0.51	0.34	0.40	0.64	0.96	0.31	0.17	0.21	0.18	0.08	0.37	0.13	0.22	0.08	0.12	0.17	0.17	0.02	0.06	0.12	0.31	0.33
interested2	0.70	0.66	0.25	0.25	0.39	0.53	0.92	0.37	0.06	0.33	0.12	0.08	0.58	0.18	0.13	0.22	0.12	0.17	0.21	0.02	0.13	0.06	0.13	0.82
joy1	0.42	0.22	0.79	0.77	0.70	0.46	0.12	0.89	0.22	0.63	0.65	0.06	0.13	0.12	0.13	0.13	0.08	0.08	0.13	0.00	0.12	0.13	0.29	0.26
joy2	0.44	0.29	0.57	0.77	0.41	0.33	0.12	0.95	0.21	0.48	0.60	0.02	0.22	0.17	0.08	0.08	0.08	0.06	0.08	0.02	0.02	0.00	0.33	0.57
joy3	0.78	0.22	0.68	0.56	0.30	0.47	0.66	0.93	0.29	0.17	0.53	0.02	0.33	0.13	0.22	0.17	0.08	0.18	0.13	0.06	0.17	0.08	0.30	0.18
joy4	0.82	0.22	0.61	0.29	0.25	0.18	0.50	0.96	0.06	0.34	0.18	0.02	0.12	0.12	0.08	0.17	0.12	0.12	0.06	0.13	0.13	0.00	0.29	0.22
loved1	0.18	0.30	0.75	0.80	0.25	0.31	0.17	0.56	0.93	0.37	0.66	0.02	0.12	0.08	0.18	0.17	0.08	0.12	0.02	0.06	0.00	0.06	0.53	0.17
loved2	0.25	0.52	0.33	0.78	0.17	0.60	0.69	0.66	0.89	0.54	0.12	0.17	0.29	0.17	0.21	0.06	0.29	0.18	0.12	0.06	0.06	0.08	0.72	0.30
loved3	0.17	0.33	0.48	0.92	0.30	0.18	0.00	0.30	0.93	0.22	0.48	0.02	0.02	0.29	0.13	0.18	0.13	0.08	0.08	0.06	0.02	0.08	0.51	0.08
loved4	0.39	0.54	0.60	0.77	0.12	0.50	0.57	0.49	0.91	0.49	0.30	0.06	0.25	0.29	0.21	0.13	0.42	0.31	0.06	0.06	0.26	0.08	0.44	0.40
loved5	0.29	0.41	0.40	0.86	0.66	0.58	0.33	0.31	0.91	0.46	0.21	0.13	0.22	0.18	0.08	0.13	0.18	0.08	0.21	0.12	0.17	0.08	0.58	0.22
proud1	0.12	0.18	0.64	0.65	0.47	0.60	0.06	0.37	0.02	0.97	0.40	0.17	0.08	0.17	0.22	0.13	0.06	0.06	0.06	0.12	0.00	0.08	0.36	0.08
proud2	0.25	0.22	0.66	0.62	0.21	0.58	0.18	0.74	0.06	0.95	0.62	0.08	0.22	0.08	0.12	0.25	0.02	0.17	0.06	0.02	0.02	0.00	0.21	0.25
proud3	0.54	0.36	0.55	0.22	0.06	0.36	0.06	0.31	0.22	0.95	0.29	0.30	0.22	0.46	0.17	0.34	0.25	0.70	0.00	0.41	0.60	0.08	0.30	0.12
proud4	0.33	0.46	0.68	0.53	0.40	0.62	0.40	0.54	0.62	0.93	0.25	0.08	0.33	0.30	0.17	0.02	0.25	0.44	0.17	0.06	0.12	0.06	0.44	0.26
relieved1	0.22	0.31	0.31	0.72	0.25	0.13	0.00	0.39	0.08	0.00	0.96	0.37	0.42	0.18	0.26	0.36	0.22	0.02	0.25	0.21	0.08	0.49	0.21	0.39
relieved2	0.26	0.13	0.41	0.71	0.33	0.12	0.13	0.53	0.06	0.26	0.97	0.40	0.30	0.21	0.18	0.26	0.21	0.13	0.13	0.13	0.13	0.48	0.13	0.08
relieved3	0.31	0.31	0.51	0.73	0.31	0.06	0.18	0.30	0.08	0.18	0.96	0.13	0.30	0.18	0.25	0.26	0.13	0.13	0.06	0.02	0.08	0.17	0.21	0.72
angry1	0.08	0.18	0.31	0.25	0.29	0.26	0.18	0.13	0.13	0.21	0.13	0.96	0.73	0.22	0.18	0.57	0.08	0.49	0.37	0.12	0.30	0.58	0.17	0.21
angry2	0.12	0.17	0.08	0.02	0.06	0.12	0.06	0.06	0.02	0.12	0.18	0.96	0.37	0.37	0.17	0.71	0.13	0.42	0.22	0.21	0.44	0.73	0.22	0.18
angry3	0.12	0.13	0.12	0.13	0.06	0.02	0.00	0.12	0.06	0.06	0.21	0.94	0.31	0.29	0.12	0.51	0.18	0.17	0.86	0.18	0.12	0.80	0.08	0.18
angry4	0.17	0.25	0.21	0.12	0.02	0.08	0.00	0.06	0.13	0.13	0.08	0.97	0.26	0.40	0.17	0.70	0.50	0.12	0.18	0.12	0.06	0.62	0.21	0.31

Item	amu	awe	con	gra	hop	ins	int	joy	lov	pro	rel	ang	anx	ash	bor	dis	emb	env	fea	gui	jea	sad	com	sur
anxious1	0.12	0.37	0.34	0.13	0.47	0.06	0.25	0.06	0.02	0.06	0.08	0.22	0.97	0.22	0.31	0.30	0.08	0.18	0.74	0.12	0.08	0.30	0.08	0.37
anxious2	0.13	0.17	0.08	0.02	0.13	0.06	0.02	0.00	0.08	0.06	0.06	0.46	0.96	0.36	0.21	0.30	0.22	0.06	0.87	0.30	0.06	0.61	0.21	0.18
anxious3	0.08	0.21	0.17	0.12	0.12	0.02	0.25	0.06	0.08	0.06	0.13	0.31	0.96	0.33	0.13	0.37	0.36	0.21	0.87	0.29	0.08	0.29	0.26	0.12
ashamed1	0.18	0.21	0.29	0.02	0.08	0.13	0.12	0.12	0.13	0.25	0.17	0.57	0.57	0.94	0.13	0.69	0.68	0.22	0.22	0.69	0.12	0.44	0.22	0.25
ashamed2	0.18	0.26	0.29	0.12	0.13	0.29	0.06	0.00	0.12	0.21	0.08	0.33	0.39	0.95	0.25	0.66	0.75	0.17	0.17	0.57	0.06	0.70	0.18	0.17
ashamed3	0.17	0.25	0.34	0.12	0.17	0.06	0.06	0.08	0.13	0.17	0.21	0.34	0.26	0.96	0.17	0.57	0.67	0.17	0.17	0.63	0.13	0.63	0.22	0.08
bored1	0.12	0.22	0.61	0.21	0.25	0.12	0.06	0.02	0.08	0.00	0.34	0.46	0.71	0.31	0.95	0.48	0.29	0.18	0.31	0.13	0.08	0.66	0.25	0.17
bored2	0.33	0.34	0.69	0.21	0.21	0.18	0.40	0.08	0.06	0.12	0.44	0.18	0.46	0.26	0.97	0.21	0.13	0.17	0.17	0.13	0.08	0.39	0.58	0.36
bored3	0.37	0.33	0.48	0.08	0.06	0.12	0.22	0.06	0.02	0.00	0.34	0.34	0.48	0.29	0.97	0.37	0.26	0.25	0.17	0.25	0.17	0.33	0.22	0.49
bored4	0.40	0.18	0.53	0.34	0.12	0.31	0.34	0.39	0.06	0.44	0.70	0.08	0.33	0.22	0.96	0.06	0.22	0.29	0.06	0.17	0.13	0.06	0.18	0.54
disgusted1	0.12	0.13	0.08	0.12	0.02	0.08	0.08	0.06	0.06	0.12	0.08	0.74	0.72	0.39	0.26	0.92	0.17	0.13	0.88	0.18	0.08	0.46	0.06	0.17
disgusted2	0.22	0.22	0.13	0.06	0.18	0.08	0.02	0.08	0.00	0.12	0.13	0.25	0.17	0.36	0.17	0.99	0.29	0.17	0.25	0.17	0.02	0.22	0.13	0.30
disgusted3	0.25	0.21	0.17	0.13	0.08	0.06	0.02	0.06	0.02	0.18	0.17	0.87	0.30	0.77	0.21	0.92	0.46	0.18	0.36	0.25	0.08	0.47	0.18	0.44
disgusted4	0.25	0.30	0.21	0.12	0.02	0.08	0.13	0.02	0.12	0.13	0.12	0.83	0.31	0.75	0.08	0.91	0.83	0.08	0.13	0.18	0.06	0.39	0.17	0.36
disgusted5	0.26	0.33	0.40	0.08	0.13	0.00	0.08	0.08	0.13	0.12	0.17	0.86	0.51	0.21	0.40	0.94	0.37	0.44	0.18	0.25	0.36	0.54	0.37	0.21
embarrassed1	0.22	0.21	0.21	0.08	0.13	0.08	0.06	0.13	0.02	0.12	0.13	0.06	0.42	0.82	0.13	0.22	0.98	0.08	0.18	0.02	0.00	0.12	0.26	0.13
embarrassed2	0.12	0.17	0.17	0.17	0.13	0.13	0.00	0.06	0.08	0.13	0.17	0.67	0.53	0.85	0.25	0.30	0.95	0.13	0.29	0.13	0.12	0.22	0.17	0.29
embarrassed3	0.34	0.51	0.29	0.60	0.12	0.42	0.08	0.41	0.42	0.61	0.13	0.36	0.41	0.51	0.18	0.44	0.93	0.25	0.08	0.26	0.13	0.29	0.25	0.65
envy1	0.13	0.18	0.29	0.08	0.42	0.30	0.36	0.02	0.12	0.18	0.22	0.52	0.44	0.17	0.22	0.22	0.18	0.93	0.13	0.13	0.90	0.13	0.21	0.17
envy2	0.25	0.40	0.25	0.33	0.25	0.44	0.49	0.25	0.13	0.22	0.06	0.12	0.22	0.13	0.02	0.17	0.17	0.94	0.06	0.06	0.90	0.02	0.31	0.22
envy3	0.21	0.25	0.26	0.08	0.76	0.36	0.31	0.06	0.08	0.12	0.17	0.12	0.47	0.22	0.18	0.18	0.12	0.94	0.08	0.29	0.83	0.54	0.22	0.06
fear1	0.12	0.13	0.21	0.08	0.08	0.12	0.06	0.06	0.00	0.13	0.00	0.68	0.81	0.26	0.22	0.22	0.13	0.21	0.97	0.02	0.22	0.26	0.17	0.25
fear2	0.13	0.12	0.18	0.12	0.08	0.08	0.02	0.12	0.02	0.21	0.21	0.54	0.78	0.18	0.13	0.18	0.17	0.13	0.97	0.08	0.02	0.41	0.26	0.31

Item	amu	awe	con	gra	hop	ins	int	joy	lov	pro	rel	ang	anx	ash	bor	dis	emb	env	fea	gui	jea	sad	com	sur
guilty1	0.12	0.21	0.21	0.06	0.08	0.06	0.12	0.12	0.12	0.13	0.17	0.49	0.29	0.91	0.08	0.30	0.54	0.06	0.26	0.92	0.06	0.67	0.30	0.17
guilty2	0.06	0.13	0.25	0.06	0.02	0.00	0.06	0.06	0.06	0.08	0.02	0.55	0.36	0.94	0.18	0.51	0.88	0.06	0.17	0.73	0.13	0.26	0.17	0.33
jealous1	0.18	0.29	0.17	0.17	0.12	0.18	0.08	0.12	0.17	0.12	0.12	0.47	0.29	0.18	0.12	0.26	0.25	0.81	0.26	0.08	0.96	0.69	0.31	0.18
jealous2	0.06	0.18	0.29	0.13	0.08	0.13	0.02	0.17	0.18	0.08	0.08	0.51	0.18	0.18	0.44	0.17	0.25	0.79	0.21	0.06	0.97	0.49	0.25	0.26
jealous3	0.25	0.18	0.13	0.29	0.17	0.12	0.36	0.17	0.49	0.17	0.17	0.33	0.30	0.33	0.18	0.21	0.22	0.83	0.12	0.02	0.96	0.30	0.41	0.17
sad1	0.13	0.25	0.18	0.00	0.02	0.12	0.12	0.06	0.13	0.06	0.12	0.87	0.48	0.57	0.18	0.58	0.40	0.18	0.08	0.29	0.18	0.94	0.12	0.48
sad2	0.13	0.12	0.13	0.13	0.08	0.06	0.06	0.06	0.08	0.08	0.17	0.65	0.33	0.17	0.25	0.31	0.13	0.12	0.33	0.22	0.13	0.98	0.22	0.21
compassionate1	0.17	0.25	0.12	0.00	0.06	0.13	0.08	0.00	0.02	0.12	0.18	0.25	0.30	0.17	0.18	0.25	0.08	0.13	0.46	0.46	0.02	0.90	0.95	0.02
compassionate2	0.21	0.17	0.18	0.47	0.44	0.48	0.52	0.06	0.26	0.39	0.26	0.21	0.41	0.25	0.22	0.18	0.30	0.13	0.08	0.17	0.02	0.34	0.97	0.08
compassionate3	0.06	0.25	0.22	0.40	0.62	0.40	0.62	0.62	0.18	0.40	0.62	0.82	0.37	0.42	0.25	0.56	0.29	0.21	0.18	0.49	0.62	0.86	0.91	0.18
surprised1	0.18	0.42	0.08	0.21	0.21	0.17	0.21	0.12	0.08	0.12	0.21	0.69	0.73	0.25	0.17	0.25	0.13	0.12	0.33	0.12	0.00	0.21	0.17	0.98
surprised2	0.29	0.39	0.17	0.22	0.25	0.17	0.13	0.12	0.13	0.12	0.17	0.13	0.62	0.17	0.22	0.21	0.17	0.06	0.51	0.06	0.00	0.02	0.17	0.98

Note: Greyed cells indicate hit rates for target responses.

Table S6

Study 1: Emotion-Level Chance-Adjusted Hit Rate (Item Difficulty) on the CORE Test (Confusion Matrix)

Emotion	amu	awe	con	gra	hop	ins	int	joy	lov	pro	rel	ang	anx	ash	bor	dis	emb	env	fea	gui	jea	sad	com	sur
amused	0.89	0.29	0.35	0.12	0.12	0.13	0.33	0.61	0.08	0.10	0.33	0.62	0.42	0.55	0.24	0.73	0.75	0.18	0.25	0.18	0.11	0.35	0.41	0.57
awed	0.42	0.93	0.19	0.29	0.31	0.72	0.39	0.27	0.09	0.36	0.17	0.10	0.54	0.29	0.19	0.15	0.28	0.37	0.58	0.07	0.22	0.04	0.29	0.74
content	0.18	0.18	0.97	0.63	0.33	0.21	0.10	0.53	0.21	0.52	0.70	0.04	0.14	0.12	0.27	0.07	0.14	0.09	0.09	0.06	0.05	0.09	0.27	0.14
grateful	0.23	0.32	0.65	0.94	0.36	0.29	0.19	0.67	0.50	0.70	0.35	0.09	0.16	0.21	0.16	0.15	0.24	0.23	0.14	0.32	0.17	0.08	0.37	0.34
hopeful	0.22	0.27	0.50	0.46	0.94	0.83	0.34	0.47	0.07	0.42	0.47	0.09	0.51	0.13	0.13	0.13	0.10	0.17	0.17	0.08	0.07	0.05	0.33	0.23
inspired	0.27	0.71	0.35	0.49	0.50	0.92	0.22	0.44	0.13	0.79	0.38	0.14	0.24	0.22	0.16	0.16	0.10	0.34	0.20	0.08	0.23	0.21	0.56	0.32
interested	0.66	0.62	0.40	0.30	0.39	0.59	0.94	0.34	0.12	0.28	0.15	0.08	0.50	0.16	0.18	0.16	0.12	0.17	0.19	0.02	0.10	0.09	0.23	0.70
joyful	0.70	0.24	0.69	0.67	0.48	0.38	0.44	0.94	0.20	0.45	0.54	0.03	0.21	0.13	0.13	0.14	0.09	0.11	0.10	0.06	0.11	0.06	0.30	0.35
loved	0.26	0.43	0.56	0.84	0.37	0.48	0.45	0.50	0.92	0.43	0.42	0.08	0.19	0.21	0.17	0.14	0.24	0.16	0.10	0.07	0.11	0.08	0.58	0.25
proud	0.35	0.33	0.64	0.55	0.32	0.56	0.20	0.55	0.33	0.95	0.43	0.17	0.22	0.28	0.17	0.20	0.16	0.44	0.08	0.19	0.28	0.06	0.34	0.19
relieved	0.27	0.26	0.42	0.72	0.30	0.11	0.11	0.42	0.07	0.16	0.96	0.32	0.34	0.19	0.23	0.30	0.19	0.10	0.16	0.13	0.10	0.41	0.19	0.51
angry	0.12	0.19	0.19	0.14	0.12	0.13	0.07	0.10	0.09	0.13	0.15	0.96	0.50	0.33	0.16	0.64	0.27	0.33	0.61	0.16	0.26	0.70	0.17	0.22
anxious	0.11	0.26	0.21	0.09	0.28	0.05	0.19	0.04	0.06	0.06	0.09	0.34	0.96	0.31	0.22	0.32	0.24	0.16	0.84	0.24	0.07	0.44	0.19	0.24
ashamed	0.18	0.24	0.31	0.09	0.13	0.17	0.08	0.07	0.13	0.21	0.16	0.43	0.43	0.95	0.19	0.65	0.70	0.19	0.19	0.64	0.11	0.61	0.21	0.17
bored	0.32	0.28	0.59	0.22	0.17	0.19	0.28	0.17	0.06	0.19	0.50	0.30	0.54	0.27	0.96	0.32	0.23	0.22	0.19	0.17	0.12	0.43	0.36	0.42
disgusted	0.22	0.25	0.21	0.10	0.09	0.06	0.07	0.06	0.07	0.13	0.13	0.80	0.48	0.60	0.24	0.95	0.56	0.22	0.60	0.21	0.14	0.43	0.20	0.31
embarrassed	0.24	0.33	0.22	0.37	0.13	0.24	0.05	0.23	0.22	0.37	0.15	0.46	0.46	0.78	0.19	0.33	0.96	0.16	0.19	0.15	0.09	0.22	0.23	0.44
envious	0.20	0.29	0.27	0.18	0.57	0.37	0.40	0.12	0.11	0.18	0.16	0.31	0.39	0.18	0.15	0.19	0.16	0.94	0.09	0.17	0.88	0.30	0.25	0.16
fearful	0.13	0.13	0.20	0.10	0.08	0.10	0.04	0.09	0.01	0.17	0.12	0.62	0.79	0.22	0.18	0.20	0.15	0.17	0.97	0.05	0.13	0.35	0.22	0.28
guilty	0.09	0.17	0.23	0.06	0.05	0.03	0.09	0.09	0.09	0.11	0.10	0.52	0.33	0.92	0.13	0.42	0.80	0.06	0.22	0.86	0.10	0.53	0.24	0.26

Emotion	amu	awe	con	gra	hop	ins	int	joy	lov	pro	rel	ang	anx	ash	bor	dis	emb	env	fea	gui	jea	sad	com	sur
jealous	0.17	0.22	0.20	0.20	0.12	0.15	0.18	0.15	0.31	0.12	0.12	0.45	0.26	0.24	0.27	0.22	0.24	0.81	0.20	0.06	0.96	0.54	0.33	0.21
sad	0.13	0.19	0.16	0.07	0.05	0.09	0.09	0.06	0.11	0.07	0.14	0.80	0.42	0.42	0.22	0.47	0.29	0.15	0.22	0.26	0.16	0.97	0.17	0.37
compassionate	0.15	0.22	0.18	0.34	0.45	0.37	0.48	0.35	0.17	0.32	0.42	0.61	0.36	0.30	0.22	0.37	0.23	0.16	0.28	0.40	0.35	0.83	0.95	0.10
surprised	0.24	0.40	0.13	0.22	0.23	0.17	0.17	0.12	0.11	0.12	0.19	0.53	0.68	0.21	0.20	0.23	0.15	0.09	0.43	0.09	0.00	0.13	0.17	0.98

Note: Greyed cells indicate hit rates for target responses.

Table S7

Full Item Set and Scoring Key for the Core Relational Themes of Emotion (CORE) Test

Item Name	CORE Item	Full Credit (1)	Half Credit (.50)	No Credit 1 (0)	No Credit 2 (0)	No Credit 3 (0)
Amused1	People often feel ______ when they find something humorous.	amused	joyful	grateful	hopeful	inspired
Amused2	People often feel ______ when something is absurd but nonthreatening.	amused	surprised	content	loved	proud
Awed1	People often feel ______ when they are in the presence of something much greater than themselves.	awed	inspired	amused	compassionate	relieved
Awed2	People often feel ______ when what's in front of them is something powerful.	awed	fearful	ashamed	bored	disgusted
Compassionate1	People often feel ______ when someone else is in pain.	compassionate	sad	embarrassed	envious	surprised
Compassionate2	People often feel ______ when someone else needs help or support.	compassionate	anxious	bored	guilty	jealous
Content2	People often feel ______ when they fully accept things as they are.	content	relieved	amused	interested	surprised
Content3	People often feel ______ when things in their life seem complete.	content	grateful	hopeful	inspired	loved
Grateful1	People often feel ______ when someone is generous toward them.	grateful	loved	hopeful	interested	proud
Grateful3	People often feel ______ when they see themselves as fortunate.	grateful	content	amused	compassionate	surprised
Hopeful3	People often feel ______ when they think something might go well.	hopeful	grateful	awed	interested	proud
Hopeful4	People often feel ______ when they think an unfavorable situation can be improved in the future.	hopeful	relieved	amused	compassionate	surprised
Inspired1	People often feel ______ when another person shows extraordinary moral courage.	inspired	awed	interested	loved	relieved
Inspired2	People often feel ______ when someone else is able to overcome extreme hardship.	inspired	awed	amused	interested	content

Joyful2	People often feel ______ when they are the recipient of good news.	joyful	grateful	amused	compassionate	hopeful
Joyful3	People often feel ______ when they are free to engage in play.	joyful	amused	awed	proud	surprised
Loved1	People often feel ______ when someone else fully accepts them for who they are.	loved	grateful	hopeful	interested	surprised
Loved3	People often feel ______ when another person is always there for them.	loved	grateful	amused	hopeful	inspired
Proud2	People often feel ______ when a goal of theirs has been achieved.	Proud	joyful	awed	compassionate	surprised
Proud3	People often feel ______ when they think that they're better than others.	Proud	content	interested	hopeful	loved
Angry1	People often feel ______ when something gets in the way of what they want.	Angry	anxious	guilty	jealous	surprised
Angry2	People often feel ______ when they believe something is unfair.	Angry	disgusted	bored	embarrassed	envious
Anxious1	People often feel ______ when something important is uncertain.	anxious	fearful	ashamed	envious	sad
Anxious3	People often feel ______ when they sense something could be wrong.	anxious	fearful	bored	guilty	jealous
Ashamed1	People often feel ______ when they behave in a way that conflicts with their own code of ethics.	ashamed	guilty	fearful	jealous	surprised
Ashamed3	People often feel ______ when they don't see themselves as an honorable person.	ashamed	sad	anxious	bored	envious
Bored1	People often feel ______ when what they're experiencing hasn't changed for a while.	Bored	angry	embarrassed	guilty	surprised
Bored3	People often feel ______ when what's in front of them doesn't seem relevant to them at all.	Bored	angry	fearful	jealous	sad
Disgusted2	People often feel ______ when what they encounter is gross.	disgusted	surprised	bored	envious	sad
Disgusted5	People often feel ______ when someone else doesn't deserve any respect at all.	disgusted	angry	fearful	guilty	surprised
Embarrassed1	People often feel ______ when they do something in public that is awkward.	embarrassed	ashamed	angry	bored	jealous

Embarrassed3	People often feel ______ when they receive unwanted praise from others.	embarrassed	ashamed	envious	fearful	sad
Envy1	People often feel ______ when they think something should be theirs.	envious	jealous	ashamed	fearful	surprised
Envy2	People often feel ______ when someone else has something valuable.	envious	jealous	angry	bored	guilty
Jealous1	People often feel ______ when someone they care about might like someone else more than them.	jealous	envious	bored	fearful	surprised
Jealous3	People often feel ______ when someone close to them pays a lot of attention to another person.	jealous	envious	anxious	embarrassed	guilty
Sad1	People often feel ______ when something did not turn out the way they wanted.	Sad	angry	bored	fearful	jealous
Sad2	People often feel ______ when something that matters to them is gone.	Sad	angry	embarrassed	envious	guilty

Note: Item numbers were retained from the original item set (see Supplemental Table 1).

Figure S1

Screenshots of the Core Relational Themes of Emotion (CORE) Test with Instructions

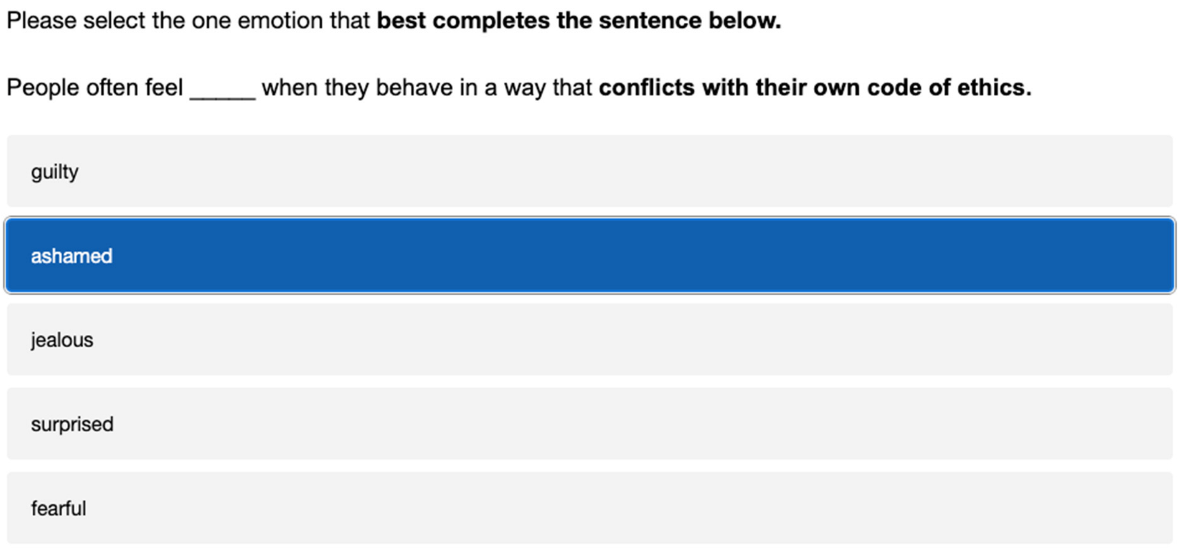

Note. The partial credit answer is “guilty” (see Table S7).

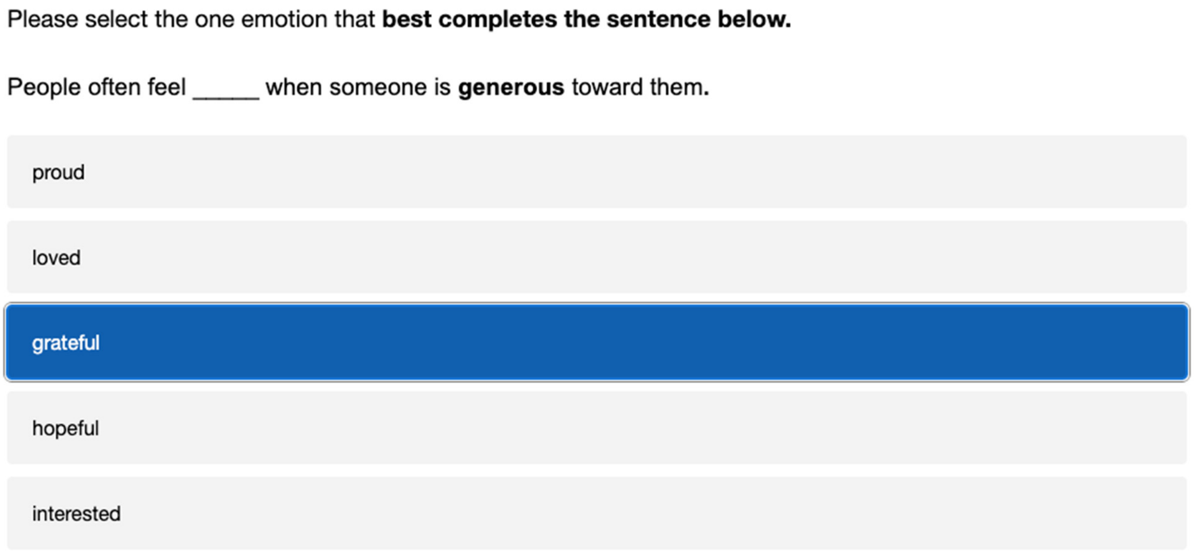

Note. The partial credit answer is “loved” (see Table S7).

Table S8

Study 2: Participant Demographic Characteristics

Demographic Characteristic	% or Mean *(SD)*
**Age**	41.2 *(14.2)*
**Gender**	
Female	50.4
Male	49.6
Non-Binary Identity	0.0
**Race/Ethnicity**	
White	66.2
Latinx	14.8
Black	12.3
Asian	6.7
**Primary Language Spoken**	
English	100
**Education Level**	
High School	27.1
Some College	10.2
Associate Degree	16.5
Bachelor's Degree	26.4
Master's Degree	15.5
Doctoral Degree	4.2
**Job Status**	
Full-time (>30 hours a week)	100.0
**Job Type**	
Other	39.1
Business or Finance	10.9
Construction or Manufacturing	10.6
Service Sector	8.5
Tech Industry	8.5
Managerial	8.1
Education or Research	7.0
Law or Medicine	4.2
Not Currently Employed	3.2

Note. $N = 284$.

Table S9

Study 2: Factor Loadings from a One-Factor Confirmatory Factor Analysis (CFA) of the CORE

Item	Standardized Factor Loading	S.E.	*p*-value
Amused1	0.74	0.04	<.001
Amused2	0.52	0.06	<.001
Awed1	0.80	0.03	<.001
Awed2	0.62	0.05	<.001
Compassionate1	0.70	0.05	<.001
Compassionate2	0.70	0.06	<.001
Content2	0.72	0.04	<.001
Content3	0.68	0.05	<.001
Grateful1	0.67	0.05	<.001
Grateful3	0.56	0.06	<.001
Hopeful3	0.73	0.05	<.001
Hopeful4	0.70	0.05	<.001
Inspired1	0.70	0.05	<.001
Inspired2	0.72	0.05	<.001
Joyful2	0.49	0.06	<.001
Joyful3	0.53	0.06	<.001
Loved1	0.67	0.05	<.001
Loved3	0.40	0.05	<.001
Proud2	0.71	0.05	<.001
Proud3	0.73	0.05	<.001
Angry1	0.57	0.06	<.001
Angry2	0.67	0.06	<.001
Anxious1	0.62	0.05	<.001
Anxious3	0.60	0.05	<.001
Ashamed1	0.44	0.06	<.001
Ashamed3	0.80	0.04	<.001
Bored1	0.71	0.05	<.001
Bored3	0.63	0.06	<.001
Disgusted2	0.87	0.04	<.001
Disgusted5	0.49	0.06	<.001
Embarrassed1	0.76	0.05	<.001
Embarrassed3	0.66	0.06	<.001
Envious1	0.53	0.05	<.001
Envious2	0.50	0.06	<.001
Jealous1	0.68	0.05	<.001
Jealous3	0.62	0.05	<.001
Sad1	0.46	0.06	<.001
Sad2	0.73	0.06	<.001

Note. Item names refer to item numbers from the original set of 78 items (see Table S3).

Table S10

Study 2: Zero-Order Correlations Among Key Study Variables and Covariates (Mean Values)

Variable	M	SD	CORE	MSCEIT	STEU	V-IQ	Relational Conflict
Covariates							
Age	41.18	14.25	.28***	.34***	.32***	.38***	-.24***
Gender (M/F)	.50	.50	.18**	.16	.19*	-.04	-.12
Race/Ethnicity (White/BIPOC)	.34	.47	-.03	-.24**	.05	-.07	.11
Education (<4-year / ≥ 4-year degree)	.46	.50	-.26***	-.17*	-.22*	-.00	.18**
Latent Variables							
CORE	.76	.20	—				
MSCEIT	.48	.16	.80***	—			
STEU	.50	.17	.86***	—	—		
V-IQ	.59	.24	.62***	.66***	.67***	—	
Relational Conflict	2.89	1.20	-.41***	-.26**	-.38***	-.26***	—

*Note. n*s = 140-284. CORE = Core Relational Themes of Emotion (CORE) Test. MSCEIT = Mayer–Salovey–Caruso Emotional Intelligence Test; STEU = Situational Test of Emotion Understanding; V-IQ = Verbal Intelligence. Participants were randomized to receive either the MSCEIT or the STEU. For the CORE, MSCEIT, STEU, and outcomes, we entered mean values into the correlations. The reference group for binary variables is the last group in all cases.

* $p < .05$ ** $p < .01$ *** $p < .001$.

Study 2: Partial Correlation and Regression Results Using Mean Values (vs. Factor Scores)

Adjusting for verbal intelligence, the partial correlations of the CORE with MSCEIT-Understanding ($r = .69, p < .001$) and the STEU ($r = .75, p < .001$) decreased but remained large.

Regarding incremental validity, adding the CORE to a multiple regression model containing demographic covariates and MSCEIT-Understanding resulted in a significant increase in the R^2, $R^2 = .17$, $F(6,131) = 4.38, p < .001$. The R^2 change (131) = .07, $p < .01$, and the total adjusted $R^2 = .13$. Also, after adding the CORE, the MSCEIT-Understanding link with relational conflict became non-significant from a trend (from $\beta = -.18, p = .06$ without to $\beta = .15, p = .26$ with the CORE), while the CORE relationship remained significant ($\beta = -.45, p < .01$).

Adding the CORE to a multiple regression model containing demographics and the STEU resulted in an increase in the R^2, $R^2 = .24$, $F(6,130) = 7.00, p < .001$. The R^2 change (130) = .04, p < .01, and the total adjusted $R^2 = .21$. By adding the CORE to the model, the STEU link with relational conflict became non-significant (from $\beta = -.27, p < .01$ without to $\beta = .07, p = .65$ with the CORE), while the CORE association remained significant ($\beta = -.41, p < .01$).

Finally, we tested whether the CORE was associated with relational conflict, accounting demographic variables and verbal intelligence. Adding the CORE to a multiple regression model containing demographics and verbal intelligence scores produced an increased R^2, $R^2 = .20$, $F(6, 269) = 10.86$ p < .001. The R^2 change (269) = .06, $p < .001$, and the total model adjusted $R^2 = .18$. The CORE remained negatively associated with relational conflict ($\beta = -.35, p < .001$), providing evidence of a test-criterion relationship between the CORE and relational conflict beyond shared variance with demographics and verbal intelligence.

Overall, the results of the analyses using mean values were comparable to the CFA-derived factor score results presented in the main text, and do not substantively alter our conclusions.

Table S11

Study 3: Participant Demographic Characteristics

Demographic Characteristic	% or Mean *(SD)*
**Age**	39.0 *(8.3)*
**Gender**	
Female	70.0
Male	30.0
Non-Binary Identity	0.0
**Race/Ethnicity**	
White	39.3
Latinx	28.9
Black	28.0
Asian	1.5
Multi-Racial/Multi-Ethnic	1.3
Native American/Alaskan Native	0.4
Other Identity	0.4
**Education Level**	
High School	0.4
Some College	4.1
Associate Degree	6.1
Bachelor's Degree	36.1
Master's Degree	45.0
Professional Degree	5.7
Doctoral Degree	2.6
**Percent of Time Employed**	
Less than 0.25	0.9
0.25 to 0.49	3.3
0.50 to 0.74	16.7
0.75 to 0.99	10.0
1.0	69.1
**Extra Work Hours Daily**	
0 hours per day	7.4
1 hour per day	16.7
2 hours per day	30.7
3 hours per day	18.7
4 hours per day	7.6
5 hours per day	7.2
6 hours per day	6.7
7 hours per day	2.0
More than 7 hours per day	3.0
**Years Working in PreK-12**	11.0 *(7.2)*
**School Mode**	
Combination of Remote/In-Person	52.2
Only Remote/Virtual	37.6

Only In-Person	8.9
Other	1.3
**Annual Income (USD)**	
Less than $20,000	0.4
$20,000 to $29,999	1.7
$30,000 to $39,999	5.2
$40,000 to $49,999	10.5
$50,000 to $59,999	20.1
$60,000 to $69,999	17.5
$70,000 to $79,999	13.8
$80,000 to $89,999	9.0
$90,000 to $99,999	7.4
$100,000 to $124,999	6.6
$125,000 to $149,999	3.9
$150,000 or more	3.9
**Roles in PreK-12 School**[a]	
General Education Teacher	46.1
School Counselor	12.6
Instructional Coach	12.6
Other	7.6
Special Ed/Gifted Ed Teacher	6.1
Psychologist	5.4
Social Worker	3.9
School Principal/School Leader	3.7
Behavior Support Professional	3.7
After School Teacher	3.5
Athletic Coach	3.0
Administration Staff	2.8
Technology Specialist	2.4
Paraprofessional	2.2
Librarian	1.3
Nurse	0.9
**COVID-19 Health Impacts**[a]	
I have been diagnosed with COVID-19.	4.1
I am currently ill from COVID-19.	0.2
I have recovered from being ill due to COVID-19.	7.2
Someone I know has been diagnosed with COVID-19.	59.3
Someone I know has gotten ill from COVID-19.	59.6
Someone I know has died from COVID-19.	32.0
Someone I care about has been diagnosed with COVID-19.	39.1
Someone I care about has gotten ill from COVID-19.	35.2

Someone I care about has died from COVID-19.	13.3
None of the Above Apply	17.2
Rather Not Answer	2.6

Note. $N = 461$.

[a] Select all that apply. Percent equals greater than 100.

Study 3: Results from a Two-Factor CFA of the Brief-COPE

The two-factor CFA of the Brief-COPE[1] showed adequate model fit, $X^2(43) = 175.49$, $p < .001$; RMSEA = .07; CFI = .88; SRMR = .05. For adaptive coping, standardized factor loadings ranged from .42 (emotional support) to .64 (problem solving). For maladaptive coping, standardized factor loadings ranged from .38 (self-blame) to .76 (behavioral disengagement). Adaptive and maladaptive coping were uncorrelated ($r = -.09$, $p = .12$). The model was retained.

Study 3: Results from A One-Factor CFA of New Emotional Labor Scale for Educators

As the emotional labor scale contained three items, a one-factor CFA was just-identified, and so fit statistics were not available. However, the standardized factor loadings were high, and ranged from .70 to .73. Based on this information, we retained our model of emotional labor.

Study 3: Results from A One-Factor CFA of the Compassion Satisfaction and Fatigue Scale (CSF) (Abbreviated)

A one-factor CFA of a five-item version of the CSF showed good model fit, $X^2(5) = 2.79$, $p = .73$; RMSEA = .01; CFI = .99; SRMR = .01. Standardized factor loadings were high, and ranged from .72 to .79. These results support the structural validity of the abbreviated CSF.

Study 3: Partial Correlation and Regression Results Using Mean Values (vs. Factor Scores)

The results of the multiple regression analysis, including demographic covariates in the model, indicated that the CORE was positively associated with adaptive coping ($\beta = .18$, $p < .01$), job satisfaction ($\beta = .26$, $p < .001$), meaning and purpose ($\beta = .38$, $p < .001$), and a malleable emotion mindset ($\beta = .41$, $p < .001$) (see Table S12 for zero-order correlations). Also, the CORE was negatively associated with maladaptive coping ($\beta = -.43$, $p < .001$), emotional exhaustion ($\beta = -.13$, $p = .03$), and compassion fatigue ($\beta = -.40$, $p < .001$). Counter to prediction,

[1] Religion, distraction, and venting strategies were not included in the final model due to factor loadings ≤ 0.20.

the CORE was positively related to emotional labor ($\beta = .27, p < .001$, an effect also found using factor scores). The emotional exhaustion effect was not significant with factor scores, but it was with mean values. Given that factor scores more accurately estimate model error and item-level variance (Rdz-Navarro et al., 2019), we retain the factor score results and conclusions.

Also, adding the CORE to a multiple regression with demographics and the STEU-B resulted in a significant increase in R^2 for six of the eight outcomes (see Tables S12 and S13). The CORE effects remained significant in expected directions (with the STEU-B in the model) for job satisfaction, meaning and purpose, a malleable emotion mindset, and maladaptive coping and compassion fatigue, supporting its incremental validity. The effects for emotional exhaustion were not significant for either the CORE or the STEU-B. The emotional labor effect (in the reverse direction) was still significant for the CORE, but no longer significant for the STEU-B. It is worth noting that the STEU-B was significantly associated with five outcomes (absent the CORE), but with the CORE in the model, these effects all became smaller or non-significant.

In summation, as with Study 2, the Study 3 results using mean values were comparable to the factor score results in the main text, and they do not meaningfully change our conclusions.

Table S12

Study 3: Zero-Order Correlations Among Key Study Variables and Covariates (Mean Values)

			EU Ability Measures		Coping		Well-Being Measures					
Variable	M	SD	CORE	STEU-B	Adaptive Coping	Maladaptive Coping	Emotional Exhaustion	Job Satisfaction	Emotional Labor	Compassion Fatigue	Meaning/ Purpose	Emotion Mindset
Covariates												
Age	39.01	8.34	.30***	.39***	-.01	-.30***	.03	.06	.17***	-.11*	.09	.20***
Gender (M/F)	.70	.46	.33***	.41***	.07	-.30***	.11*	.03	.16***	-.21***	.05	.16***
Race/Ethnicity (White/BIPOC)	.61	.49	-.59***	-.71***	-.14**	.41***	-.21***	.01	-.33***	.18***	-.17***	-.30***
Education (< Master's Degree / ≥ Master's)	.53	.50	.41***	.45***	.12**	-.31***	.18***	-.03	.27***	-.03	.09	.22***
Income	6.57	2.40	.06	.07	-.02	-.07	.18***	-.03	.12*	.07	.05	.03
EU Ability Measures												
CORE	.73	.25	—									
STEU-B	.44	.24	.85***	—								
Coping												
Adaptive Coping	3.28	.62	.20***	.20***	—							
Maladaptive Coping	2.45	.84	-.56***	-.59***	-.08	—						
Well-Being Measures												
Emotional Exhaustion	3.57	1.48	.06	.17**	-.04	.16***	—					
Job Satisfaction	4.60	1.04	.15**	.06	.24***	-.12*	-.48***	—				
Emotional Labor	4.00	.75	.39***	.44***	.11*	-.11*	.25***	.12*	—			
Compassion Fatigue	2.97	1.18	-.37***	-.34***	-.01	.52***	.42***	-.17***	.11*	—		
Meaning/Purpose	4.09	.76	.32***	.28***	.35***	-.22***	-.19***	.51***	.29***	-.18***	—	
Emotion Mindset	3.57	.78	.46***	.37***	.30***	-.40***	-.22***	.23***	.17***	-.32***	.41***	—

Note. ns = 306-460. EU = emotion understanding. CORE = Core Relational Themes of Emotion Test. STEU-B = Situational Test of Emotion Understanding-Brief. Emotion Mindset = malleable versus fixed emotion mindset. For the CORE, STEU-B, and outcome variables, we entered mean values into the correlations. The reference group for binary variables is the last group in all cases.

* $p < .05$ ** $p < .01$ *** $p < .001$.

Table S13

Study 3: Multiple Regression Analyses Testing Incremental Validity of the CORE Above Demographics and the STEU-B with Outcomes Measuring Adaptive Functioning (Mean Values)

	DV: Adaptive Coping			DV: Job Satisfaction			DV: Meaning and Purpose			DV: Emotion Mindset		
Step	*β*	*t*	SE	*β*	*t*	SE	*β*	*t*	SE	*β*	*t*	SE
Step 1												
(Constant)		14.65	.21		9.83	.39		12.71	.28		12.24	.27
Age	-.03	-.54	.01	.08	1.15	.01	-.02	-.28	.01	.01	.10	.01
Gender	.07	1.11	.08	.04	.61	.14	-.05	-.74	.10	.02	.29	.10
Race	.09	1.10	.10	.09	1.13	.18	.06	.79	.13	-.07	-.95	.13
Education	.10	1.50	.08	-.11	-1.61	.14	-.02	-.27	.10	.02	.28	.10
Income	-.11	-1.78	.02	-.02	-.38	.03	.03	.44	.02	-.08	-1.39	.02
STEU-B	.20*	2.33	.21	.13	1.50	.38	.35***	4.17	.27	.30***	3.63	.26
R^2		.06			.02			.08			.14	
Step 2												
(Constant)		13.37	.23		8.27	.41		10.74	.29		10.16	.27
Age	-.03	-.53	.01	.08	1.16	.01	-.02	-.25	.01	.01	.15	.01
Gender	.07	1.14	.08	.05	.80	.14	-.04	-.62	.10	.03	.46	.09
Race	.10	1.20	.10	.12	1.41	.18	.10	1.27	.13	-.03	-.44	.12
Education	.09	1.40	.08	-.13	-1.88	.14	-.04	-.66	.10	-.01	-.15	.09
Income	-.10	-1.71	.02	-.01	-.12	.03	.04	.71	.02	-.06	-1.14	.02
STEU-B	.11	.94	.29	-.12	-.93	.54	.00	.01	.37	-.10	-.90	.35
CORE	.11	1.07	.12	.31**	2.82	.22	.45***	4.45	.15	.51***	5.22	.14
R^2/ R^2 change		.06/.00			.05/.03**			.14/.06***			.21/.07***	
	F (7, 297) = 2.85**			F (7, 293) = 2.16*			F (7, 297) = 6.92***			F (7, 297) = 11.47***		

Note. STEU-B = Situational Test of Emotional Understanding-Brief; CORE = Core Relational Themes of Emotion Test. Emotion Mindset = malleable versus fixed emotion mindset. For the CORE, STEU-B, and outcome variables, we entered mean values into the regression models. A separate regression model was conducted for each outcome given the intercorrelations between variables. Gender (male = 0, female = 1); race (White = 0, BIPOC = 1); and education (less than four-year college degree = 0, 1 = four-year college degree or higher). The reference group for binary variables is the last group in all cases.
* $p < .05$ ** $p < .01$ *** $p < .001$.

Table S14

Study 3: Multiple Regression Testing Incremental Validity of the CORE Above Demographics and the STEU-B with Outcomes Measuring Maladaptive Functioning (Mean Values)

	DV: Maladaptive Coping			DV: Emotional Exhaustion			DV: Emotional Labor			DV: Compassion Fatigue		
Step	*β*	*t*	SE	*β*	*t*	SE	*β*	*t*	SE	*β*	*t*	SE
Step 1												
(Constant)		15.11	.24		6.78	.51		13.53	.25		8.77	.40
Age	-.04	-.83	.01	$-.15^{*}$	-2.31	.01	-.04	-.61	.01	-.02	-.38	.01
Gender	$-.17^{**}$	-3.35	.09	.05	.85	.19	.00	.03	.09	-.12	-1.96	.15
Race	.01	.09	.12	-.08	-.98	.24	.00	.04	.12	.08	1.00	.19
Education	-.09	-1.71	.09	.07	1.07	.19	.09	1.45	.09	.11	1.73	.15
Income	-.01	-.12	.02	$.22^{***}$	3.69	.04	.05	.97	.02	.08	1.29	.03
STEU-B	$-.46^{***}$	-6.67	.24	.10	1.13	.50	$.41^{***}$	5.15	.24	$-.28^{**}$	-3.41	.39
R^2		.39			.09			.20			.15	
Step 2												
(Constant)		15.72	.25		6.82	.55		11.59	.26		9.74	.42
Age	-.04	-.88	.01	$-.15^{*}$	-2.32	.01	-.04	-.62	.01	-.02	-.39	.01
Gender	$-.17^{***}$	-3.52	.09	.05	.76	.19	.02	.26	.09	$-.13^{*}$	-2.25	.15
Race	-.02	-.29	.11	-.09	-1.11	.24	.03	.38	.12	.05	.65	.19
Education	-.08	-1.42	.09	.08	1.19	.19	.07	1.15	.09	$.13^{*}$	2.10	.15
Income	-.02	-.33	.02	$.21^{***}$	3.55	.04	.07	1.31	.02	.06	.96	.03
STEU-B	$-.22^{*}$	-2.37	.32	.21	1.76	.71	.13	1.16	.34	.03	.25	.55
CORE	$-.31^{***}$	-3.65	.13	-.15	-1.36	.28	$.35^{***}$	3.57	.14	$-.39^{***}$	-3.80	.22
R^2/ R^2 change		$.41/.03^{***}$			.09/.01			$.24/.03^{***}$			$.19/.04^{***}$	
	F (7, 297) = 29.80^{***}			F (7, 293) = 4.35^{***}			F (7, 293) = 12.90^{***}			F (7, 293) = 9.53^{***}		

Note. STEU-B = Situational Test of Emotional Understanding-Brief; CORE = Core Relational Themes of Emotion Test. For the CORE, STEU-B, and outcome variables, we entered mean values into the regression models. A separate regression model was conducted for each outcome given the intercorrelations between the variables. Gender (male = 0, female = 1); race (White = 0, BIPOC = 1); and education (less than four-year college degree = 0, 1 = four-year college degree or higher). The reference group for binary variables is the last group in all cases.
$p < .05$ $^{**}p < .01$ $^{***}p < .001$.

Study 3: Regression Analyses Probing the Race–CORE Association

As in prior EI research (Joseph and Newman, 2010; Mayer et al., 2008ab), in Study 3, age, female gender, and education level were moderately positively associated with CORE performance. That said, in Study 3, BIPOC identity was negatively related to age, female gender, and education level, which may have contributed to a race–CORE association. Also, BIPOC participants were more likely to work part-time, but engage in a greater number of 'extra hours' of (unpaid) work, as well as to report severe impacts from COVID-19 (i.e., they contracted the virus and/or a loved one fell sick and/or died from it). These additional stressors may have increased time pressure, sleep problems, or other variables that impact test performance (Pascoe et al., 2020; Shields et al., 2016). To explore this possibility, we conducted a multiple regression model (controlling for age, gender, education, work hours, extra work hours, and COVID-19 impact). The inverse association decreased but remained significant (from $\beta = -.64$ to $\beta = -.42$, ps $< .001$). This effect size is similar to the few prior studies reporting links between race and EU ability (Chan et al., 2014; Fiori and Antonakis, 2011; Joseph and Newman, 2010). The STEU-B showed a similar-sized negative association with BIPOC identity in Study 3 as well, a relationship that was not found in Study 2 (similar to the CORE). To test whether the CORE was uniquely related to race, we re-ran the same multiple regression with the same demographic covariates including the STEU-B in the model, and then BIPOC identity only showed a small inverse association with the CORE ($\beta = -.12$, $p < .01$). It appears the link between race and the CORE may be a more general link between EU ability tests and race (e.g., Joseph and Newman, 2010).
